# Causal association of type 2 diabetes with amyotrophic lateral sclerosis: new evidence from Mendelian randomization using GWAS summary statistics

**DOI:** 10.1186/s12916-019-1448-9

**Published:** 2019-12-04

**Authors:** Ping Zeng, Ting Wang, Junnian Zheng, Xiang Zhou

**Affiliations:** 1 Department of Epidemiology and Biostatistics, School of Public Health, Xuzhou Medical University, Xuzhou, 221004 Jiangsu China; 2 Cancer Institute, Xuzhou Medical University, Xuzhou, 221004 Jiangsu China; 3 Center of Clinical Oncology, Affiliated Hospital of Xuzhou Medical University, Xuzhou, 221004 China; 4 Jiangsu Center for the Collaboration and Innovation of Cancer Biotherapy, Cancer Institute, Xuzhou Medical University, Xuzhou, 221004 Jiangsu China; 5 Department of Biostatistics, University of Michigan, Ann Arbor, MI 48109 USA; 6 Center for Statistical Genetics, University of Michigan, Ann Arbor, MI 48109 USA

**Keywords:** Amyotrophic lateral sclerosis, Type 2 diabetes, Two-sample Mendelian randomization, Instrumental variable, Genome-wide association studies, East Asian, European

## Abstract

**Background:**

Associations between type 2 diabetes (T2D) and amyotrophic lateral sclerosis (ALS) were discovered in observational studies in both European and East Asian populations. However, whether such associations are causal remains largely unknown.

**Methods:**

We employed a two-sample Mendelian randomization approach to evaluate the causal relationship of T2D with the risk of ALS in both European and East Asian populations. Our analysis was implemented using summary statistics obtained from large-scale genome-wide association studies with ~660,000 individuals for T2D and ~81,000 individuals for ALS in the European population, and ~191,000 individuals for T2D and ~4100 individuals for ALS in the East Asian population. The causal relationship between T2D and ALS in both populations was estimated using the inverse-variance-weighted methods and was further validated through extensive complementary and sensitivity analyses.

**Results:**

Using multiple instruments that were strongly associated with T2D, a negative association between T2D and ALS was identified in the European population with the odds ratio (OR) estimated to be 0.93 (95% CI 0.88–0.99, *p* = 0.023), while a positive association between T2D and ALS was observed in the East Asian population with OR = 1.28 (95% CI 0.99–1.62, *p* = 0.058). These results were robust against instrument selection, various modeling misspecifications, and estimation biases, with the Egger regression and MR-PRESSO ruling out the possibility of horizontal pleiotropic effects of instruments. However, no causal association was found between T2D-related exposures (including glycemic traits) and ALS in the European population.

**Conclusion:**

Our results provide new evidence supporting the causal neuroprotective role of T2D on ALS in the European population and provide empirically suggestive evidence of increasing risk of T2D on ALS in the East Asian population. Our results have an important implication on ALS pathology, paving ways for developing therapeutic strategies across multiple populations.

## Background

Amyotrophic lateral sclerosis (ALS) is an adult-onset neurodegenerative disease characterized by rapid motor neuron degeneration and subsequent respiratory failure [1]. ALS is relatively rare worldwide: the standardized disease incidence is about 1.89 and 0.83 per 100,000 person-years of follow-up in the European and East Asian populations, respectively [2]. However, the burden of ALS is substantial. Clinically, ALS patients often suffer from loss of independence due to progressive functional impairments of upper and lower motor neurons. In addition, ALS patients only have a median survival time of 2–4 years starting from disease onset and less than 10% patients can survive beyond 10 years [3]. Economically, the annual cost per ALS patient is considerable: it ranges from ~$11,000 in Denmark to ~$70,000 in the USA [4]. Subsequently, the population-wide national economic burden of ALS is estimated to be ~$1.023 billion in the USA, far greater than that of the other two common neuromuscular diseases (Duchenne muscular dystrophy and myotonic dystrophy) [5]. Importantly, ALS incidence is expected to increase due to population aging worldwide, further aggravating the socioeconomic burden associated with ALS in the coming years [6]. Therefore, it becomes critically important to understand the etiology of ALS and identify risk factors that can causally influence ALS. Identifying causal risk factors for ALS can potentially lead to the discovery of new pathogenic pathways underlying ALS, guide the development of effective medical treatment and patient care, and facilitate healthcare policy making and healthcare resource allocation.

While the causes and pathogenesis of ALS remain largely unknown, several genetic and environmental risk factors have been identified to be associated with the development of ALS [7–9]. Among these identified risk factors, the association between antecedent diseases and ALS is of particular interest. Specifically, previous observational epidemiological studies have found that ALS patients tend to have less antecedent diseases (e.g., liver/lung/thyroid disease, diabetes, hypertension, hyperlipidemia, and arthritis) as compared to the general age/gender/geography-matched control patients [10–13]. In addition, the presence of antecedent diseases appears to lead to a substantial delay in the onset age of ALS patients [10–13]. The evidence suggests that antecedent diseases may be markers of causal risk factors for ALS or may themselves be involved in the pathogenesis of ALS. Importantly, several pieces of evidence have recently emerged to support a potentially pathological role of one antecedent metabolic disease, type 2 diabetes (T2D), in ALS. The association between T2D and ALS was first discovered a decade ago [12]. Follow-up observational studies and hospital disease registries have provided additional empirical evidence supporting the association between T2D and ALS (Table 1). This association evidence, when further paired with the observations that high energy consumption often accompanies ALS progression [23, 24] and the observations that glucose intolerance and insulin resistance have been linked to ALS [25, 26], leads to a hypothesis that T2D may mechanically and causally affect ALS.

**Table 1:** Estimated effect sizes of T2D on ALS in previous observational studies

First author	Year	Nation	Effect size (95% CI)	Ref
DOvidio	2018	Italy	0.30 (0.19–0.45)	[14]
Visser	2017	Netherlands	0.77 (0.33–1.21)	[15]
Hollinger	2016	USA	0.80 (0.53–1.21)	[10]
Mitchell	2015	USA	0.47 (0.38–0.58)	[11]
Mariosa	2015	Sweden	0.79 (0.68–0.91)	[16]
Seelen	2014	Netherlands	0.72 (0.51–1.01)	[17]
Turner	2013	England	0.98 (0.85–1.13)	[18]
Kioumourtzoglou	2015	Denmark	0.61 (0.46–0.80)	[19]
Moglia	2017	Italy	1.05 (0.78–1.42)	[20]
Korner	2012	Germany	1.11 (0.76–1.60)	[21]
Armon	1991	USA	1.00 (0.29–3.50)	[13]
Sun	2015	China	1.35 (1.10–1.67)	[22]
Pool 1			0.73 (0.59–0.90)	
Pool 2			0.77 (0.62–0.96)	

Pool 1: the effect size estimated without the study of Sun [22] as it was performed on the East Asian population; pool 2: the effect size estimated with all the studies; both estimations were generated by random-effects meta-analysis models

Unfortunately, establishing a causal relationship between T2D and ALS has been challenging so far. Almost all previous studies on T2D and ALS are observational in nature, and observational studies have inherent drawbacks that make it hard for them to reach a causal conclusion using standard statistical tools. In particular, while these previous observational studies have attempted to adjust for the effects of many confounding factors (e.g., income, education, marital status, age, gender, and race) (Additional file 1: Table S1), it is not possible to control for all confounding factors there. Unadjusted confounding factors can potentially bias the association evidence between T2D and ALS. Moving beyond observational studies is not straightforward either. For example, because ALS is rare in the population, it becomes difficult to collect large samples to carry out longitudinal studies for examining the influence of T2D on ALS [27]. In addition, due to ethical considerations, it is almost impossible to validate the causal association between T2D and ALS directly by performing randomized controlled trails. Therefore, it remains unclear whether the association between T2D and ALS observed in previous studies is causal or not: is T2D protective against ALS or is the absence of T2D just an early manifestation of ALS [10, 11, 14, 19, 28, 29]?

Besides a lack of evidence on the causal relationship between T2D and ALS, likely also due to the observational nature of previous studies, there is a lack of consensus on whether T2D is protective for ALS in all human populations. For example, in the European population, despite minor conflicting results, most observational studies found that T2D is associated with decreased susceptibility to ALS (Additional file 1: Figure S1), suggesting a possible neuroprotection role of T2D on ALS. In contrast, however, in the East Asian population, it was found that T2D can increase the risk of ALS [22].

Mendelian randomization (MR) is an advanced statistical method that can help establish a causal relationship between an exposure of interest (e.g., T2D in the present study) and an outcome of interest in observational studies by employing single-nucleotide polymorphisms (SNPs) as instrumental variables for the exposure [30–36]. MR relies on the idea that SNPs associated with T2D would also be associated with the risk of ALS through the path of T2D, if T2D is causally associated with ALS. Therefore, even though SNPs that are selected as instruments are not causal for T2D but are only associated with T2D, MR can still help establish the causal association between T2D and ALS [37]. Large-scale genome-wide association studies (GWASs) on T2D performed in the recent years have identified many SNPs associated with T2D, making it feasible to choose appropriate SNPs to serve as valid instruments for T2D [38].

To ensure the validity of the causal conclusion from MR, each selected instrumental variable needs to satisfy three MR modeling assumptions (Additional file 1: Figure S2A and Additional file 2) [39–41]: (i) it should be strongly associated with T2D; this is referred to as the relevance assumption; (ii) it should not be associated with any other confounders that may be associated with both T2D and ALS; this is referred to as the independence assumption; (iii) it influences ALS only through the path of T2D and does not have horizontal pleiotropic effects; this is referred to as the exclusion restriction assumption. Note that the first assumption (i.e., the relevance assumption) can be directly tested based on the observed data while the last two assumptions (i.e., the independence and exclusion restriction assumptions) are difficult to validate in practice. We will later examine the validity of the last two assumptions through various sensitivity analyses.

In the present study, our main objective is to investigate the causal relationship between T2D and ALS in both the European and East Asian populations. To achieve this objective, we conducted the largest two-sample MR analysis to date based on summary statistics publicly available from large-scale GWASs with ~63,000 cases for T2D and ~42,000 cases for ALS in the European population, and with ~191,000 individuals for T2D and ~4100 individuals for ALS in the East Asian population.

## Methods

### GWAS data sources and instrument selection

**Fig. 1:**
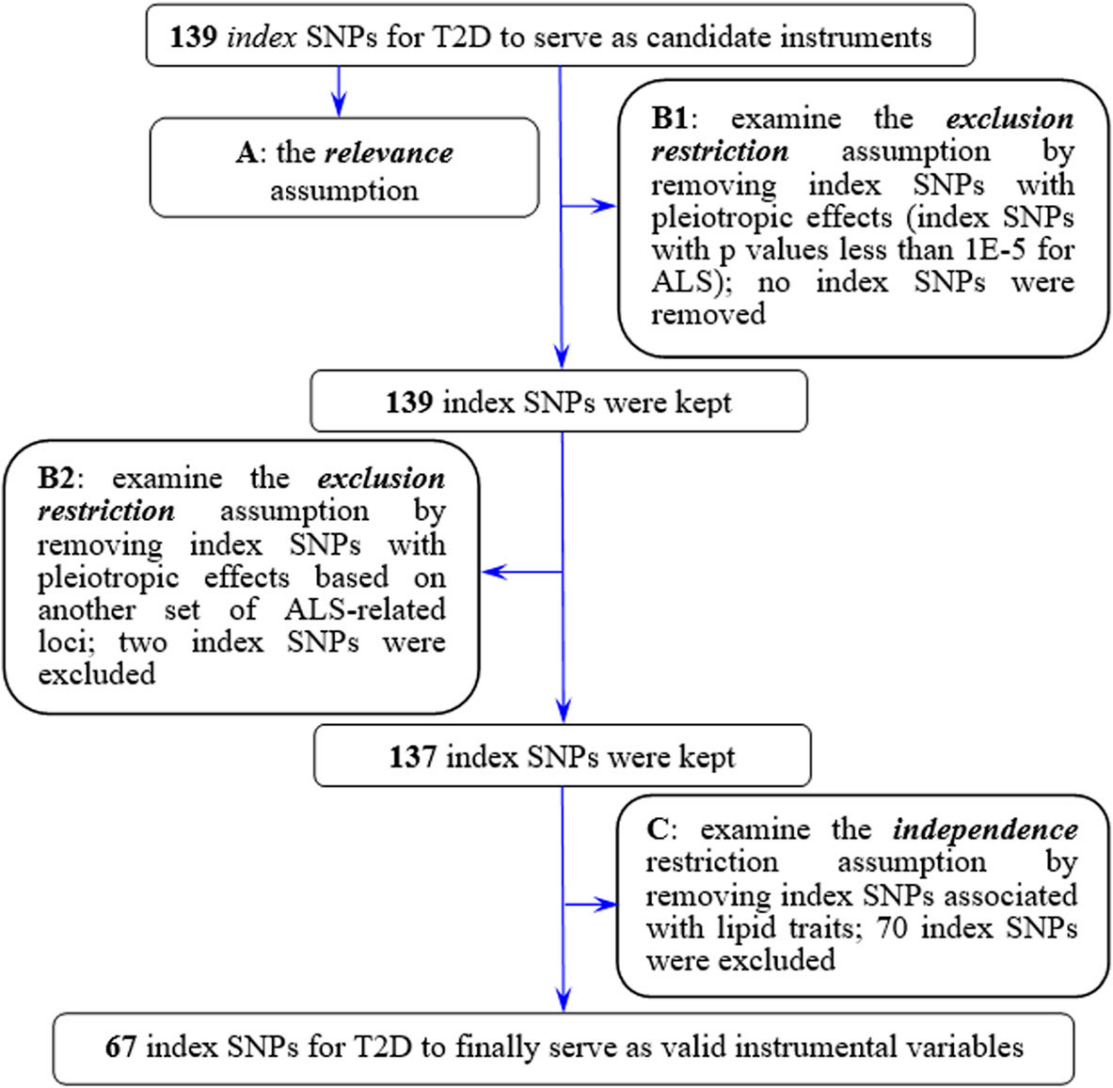
Flowchart displays the selection process for instrumental variables of T2D to investigate the causal effect of T2D on ALS in the Mendelian randomization analysis. A: a set of index SNPs (*p* < 5.00E−8) were selected to ensure the relevance assumption was satisfied; we further used the *F* statistic [46, 47] to examine the strength of those index SNPs. B1: following previous studies [48], we attempted to exclude pleiotropic associations by removing index SNPs which were likely associated with ALS with a marginal *p* value less than 1.00E−5 (the genome-wide suggestive significance level); no index SNPs were removed by this strategy in our analysis; B2: we removed index SNPs which may potentially be in linkage disequilibrium with ALS-associated loci (identified in [49], Additional file 1: Table S7), if the index SNP position was within 1 Mb of an ALS-associated locus. C: similar to B2, based on previous GWAS results of lipid traits [50, 51], we removed index SNPs that were associated with lipids, since dyslipidemia may be linked to both T2D and ALS [45, 52–54]

A crucial step of MR is to choose appropriate genetic variants to serve as valid instrumental variables for T2D. To do so, we obtained association results from one of the largest T2D GWASs to date [38], which was a genome-wide meta-analysis on three previous T2D studies: DIAbetes Genetics Replication and Meta-analysis (DIAGRAM) [42], Genetic Epidemiology Research on Adult Health and Aging (GERA) [43], and UK Biobank cohort [44]. Together, the T2D GWAS contained a total of ~16 million genotyped and imputed SNPs for 659,316 (62,892 T2D cases and 596,424 controls) individuals of European ancestry. Based on this GWAS data set, we followed other previous MR studies (e.g., [41, 45]) with a stringent selection procedure as described in Fig. 1. Specifically, we obtained a total of 139 index SNPs for T2D as candidate instrumental variables. We removed SNPs that have potential pleiotropic associations with ALS (defined by an ALS association *p* value below the genome-wide suggestive significance level of 1.00E−5). We also excluded lipid-associated index SNPs as blood lipid levels have been shown to be associated with both T2D and ALS [45, 52]. Note that the removal of pleiotropic SNPs is a conservative strategy and doing so can often ensure the validity of Mendelian randomization analysis with more confidence [33, 34, 45, 55–57]. However, to avoid the concern of excluding too many SNPs with potentially vertical pleiotropic effects [58, 59], we also performed sensitivity analysis with all 139 index SNPs included. Finally, we kept a total of 67 independent index SNPs (*p* < 5.00E−8) to serve as instrumental variables for T2D (Table 2). We obtained summary statistics (e.g., marginal effect sizes, their standard error, and effect allele) of these index SNPs for T2D from http://cnsgenomics.com/data.html. In addition, we also obtained association results from the largest ALS GWAS to date: a case–control study that analyzed ~10 million genotyped and imputed SNPs on 80,610 individuals (20,806 ALS cases and 59,804 controls) of European ancestry [49]. We obtained the summary statistics of ALS for the same set of instrument variables from the ALS Variant Server [49].

**Table 2:** GWAS data sets used in the present MR analysis

Traits	Pop	*k*_1_/*k*_0_	Sample size (case/control)	Data source
ALS	EUR		20,806/59,804	AVS [49]
T2D	EUR	67/139	62,892/596,424	PCTG [38]
BMI	EUR	91/95	339,224	GIANT [60]
Fasting glucose	EUR	35/35	133,010	MAGIC [61]
Fasting insulin	EUR	14/14	108,557	MAGIC [61]
HbA1c	EUR	36/37	123,665	MAGIC [62]
ALS	EAS		1234/2850	Benyamin [63]
T2D	EAS	34/72	36,614/155,150	JENGER [64]

Here *k*_1_ is the final number of instruments employed in the analysis while *k*_0_ is the number of candidate instruments. For T2D, we conducted stringent procedures (Fig. 1) to carefully choose instruments. For the remaining exposures, we performed the standard B1 and B2 selection procedures shown in Fig. 1 to select the final set of instrumental variables

*T2D* type 2 diabetes, *BMI* body mass index, *ALS* amyotrophic lateral sclerosis, *HbA1c* hemoglobin A1c, *GIANT* Genetic Investigation of ANthropometric Traits Consortium, *MAGIC* Meta-Analyses of Glucose and Insulin-related traits Consortium, *JENGER* Japanese ENcyclopedia of GEnetic associations by Riken, *CHN* China Health and Nutrition Survey, *AVS* ALS Variant Server, *PCTG* Program in Complex Trait Genomics, *EUR* European, *EAS* East Asian

While we primarily focused on examining the causal association between T2D and ALS, we also attempted to perform MR to estimate the causal effects of multiple T2D-related glycemic/anthropometric traits on ALS in the European population. These traits include fasting glucose [61], fasting insulin [61], hemoglobin A1c (HbA1c) [62], and body mass index (BMI) [60]. Using a selection procedure similar to that shown above, we obtained the SNP instrumental variables (Table 2) from the corresponding GWASs (Additional file 1: Tables S2–S6). The GWAS genetic data sets employed in the present study are summarized in Table 2, with more details shown in Additional file 3.

### Complementary and statistical analysis

For each index SNP that was selected as an instrument variable, we first quantified whether it was strongly associated with the exposure (e.g., T2D) or not. To do so, we calculated the phenotypic variance for the exposure variable explained (in the observed scale) by an individual instrument following the method in [65] and then computed the *F* statistic [46, 47]. Afterwards, we carried out the two-sample MR to estimate the causal effect of T2D on ALS by applying both fixed-effects and random-effects inverse-variance-weighted (IVW) methods (see Additional file 2 for more descriptions on MR estimation methods) [47, 66–68]. We also created informative plots (e.g., SNP effect scatter and funnel plots) to illustrate the results.

Besides the above main analysis, we evaluated for potential violations of the model assumptions in the MR analysis by performing a number of complementary sensitivity analyses: (i) leave-one-out (LOO) cross-validation analysis [48] and Mendelian Randomization Pleiotropy RESidual Sum and Outlier (MR-PRESSO) analysis [69] for outlier instrument detection; (ii) weighted median-based method for examining result robustness when some instruments may be potentially invalid [70]; (iii) MR-Egger regression, where its intercept was used to evaluate the directional pleiotropy of instruments [67, 71]. To further exclude bias due to horizontal pleiotropy, we also searched PhenoScanner [72] and the NHGRI-EBI GWAS Catalog to examine whether some of the selected instruments may have association with other diseases or traits. We found that 17 instrumental variables were associated with some diseases or traits (e.g., blood pressure and sugar levels) in these databases (Additional file 1: Table S7). To avoid estimation bias due to these pleiotropic associations, we excluded these 17 instruments and carried out another IVW analysis to estimate the causal effect of T2D on ALS: (iv) reverse causal inference on T2D using ALS instrument variables to explore whether ALS has a causal impact on T2D (Additional file 1: Table S8); (v) IVW methods for estimating the causal effect of T2D [64] on ALS [63] in East Asians, where both the T2D and ALS GWAS summary statistics were obtained from East Asian individuals (Table 2 and Additional file 1: Table S9).

We matched the effect/alternative allele of instrument variables between exposures (e.g., T2D) and ALS during the analysis. The main statistical analyses were conducted within the R (version 3.5.2) environment for statistical computing. The statistical significance level was set to 0.05 throughout our study. Note that participants had given informed consent for data sharing as described in each of the original GWASs employed in the present manuscript. Therefore, additional ethical review was not needed for our study.

## Results

### Causal effect of T2D on ALS

A total of 67 instrumental variables of T2D were carefully selected (Fig. 1). All the selected instruments together explain about 0.61% phenotypic variation of T2D at the observed scale (Additional file 1: Table S2). For these instrumental variables, all the *F* statistics are above 10 (ranging from 29.8 to 251.0) with an average *F* statistic of 59.8 and an overall *F* statistic of 60.2 (Additional file 1: Table S2), indicating that they satisfy the strong relevance assumption of MR and that the weak instrument bias would not substantially influence the estimations of causal effects.

**Fig. 2:**
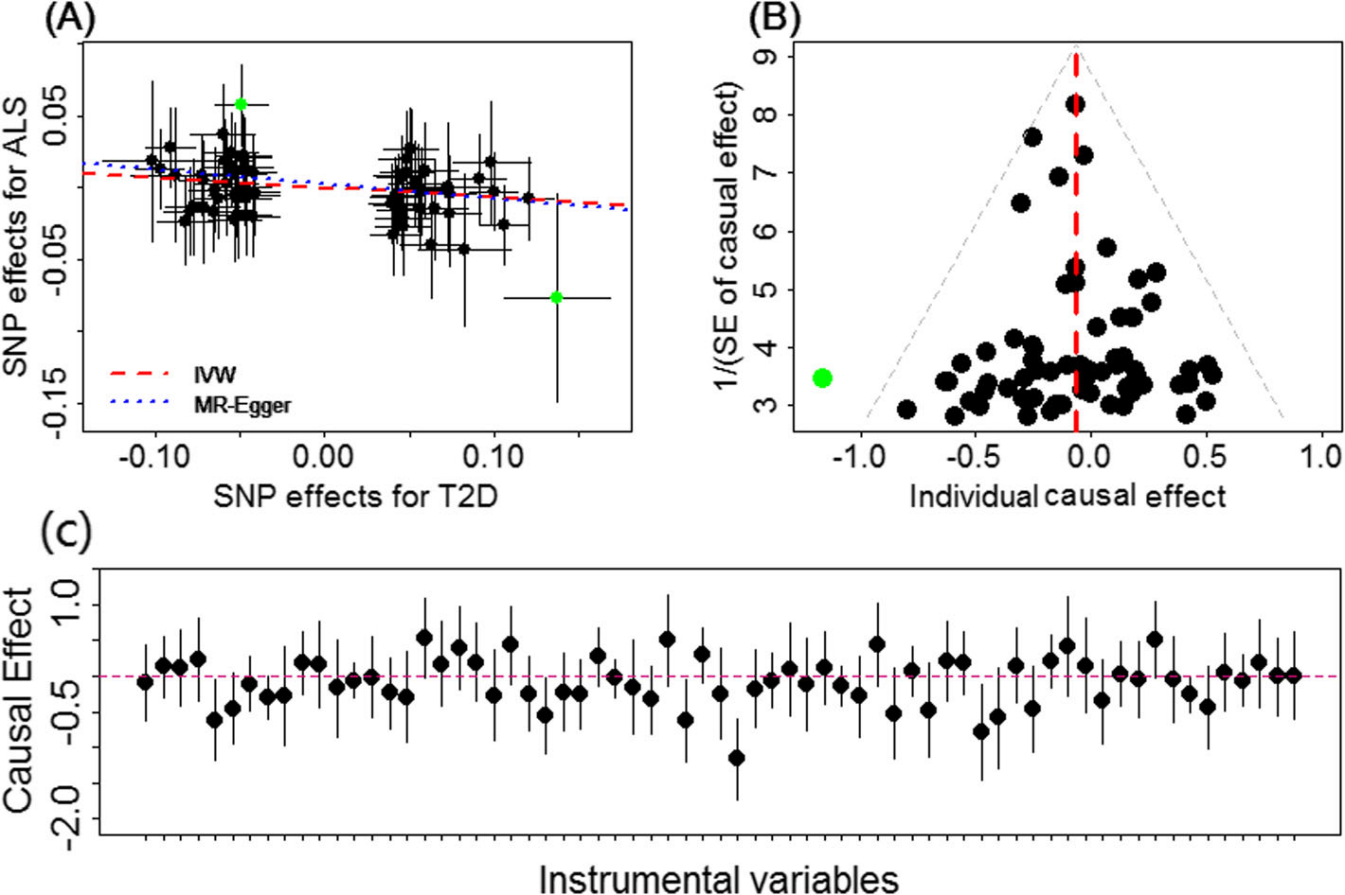
**a** Relationship between the effect size estimates on T2D (*x*-axis) and the effect size estimates on ALS (*y*-axis) for all SNPs that served as instrumental variables for T2D in the European population. Here, a total of 67 T2D instrumental variables were employed. The 95% confidence intervals for the estimated SNP effect sizes on ALS are shown as vertical black lines, while the 95% confidence intervals for the estimated SNP effect sizes on T2D are shown as horizontal black lines. The slope of fitted lines represents the estimated causal effect of T2D on ALS obtained using either the IVW method (red lines) or the MR-Egger regression (blue lines). Two possible SNP outliers (i.e., rs7729395 and rs1758632) are highlighted in green. **b** Funnel plot displays individual causal effect estimates for T2D on ALS in the European population. The dots represent the estimated causal effect for each instrumental variable. The vertical dotted red line represents the estimated causal effect obtained using all instrumental variables. A possible outlier (i.e., rs1758632) is highlighted in green. **c** Forest plot for individual causal effect estimate

Combining all the instruments together, we found that T2D is negatively associated with ALS. Specifically, in terms of the fixed-effects IVW method, the odds ratio (OR) of T2D on ALS is estimated to be 0.93 (95% CI 0.88–0.99, *p* = 0.023), suggesting that being in T2D status can lead to an average of 6.57% (95% CI 0.01–11.8%) reduction in the risk of ALS (Fig. 2a). This inverse association suggests that T2D may play a causal neuroprotective role on the development of ALS. The random-effects IVW method yields similar results, with a slightly conservative confidence interval (OR = 0.93, 95% CI 0.87–1.00, *p* = 0.055) due to the higher estimation variation resulting from considering the instrumental heterogeneity into model fitting. Using all the original 139 T2D instrumental variables, we obtained a similar negative causal effect of T2D on ALS using the random-effects IVW method (OR = 0.96, 95% CI 0.92–1.00, *p* = 0.046), supporting the robustness of the main results. This inverse association can also be observed when another set of 90 T2D instruments obtained from a separate study was used (description of the data is provided in Additional file 3; results are shown in Additional file 1: Table S10), further supporting our results.

Besides estimating the causal effect of T2D on ALS, we also evaluated the causal effects of four T2D-related glycemic/anthropometric traits (BMI, fasting glucose, fasting insulin, and HbA1c) on ALS in the European population using IVW methods. However, no statistically significant causal associations are identified between each of those exposures and ALS. Specifically, in terms of the random-effects IVW method, the ORs for a unit change of BMI (where one unit equals ~4.8 kg/m^2^), fasting glucose (mmol/L), fasting insulin (pmol/L), and HbA1c (%) on ALS are estimated to 1.00 (95% CI 0.87–1.16, *p* = 0.931), 1.09 (95% CI 0.90–1.31, *p* = 0.370), 0.78 (95% CI 0.49–1.24, *p* = 0.286), and 0.97 (95% CI 0.67–1.39, *p* = 0.861), respectively. Details of the unit are further provided in Additional file 3.

### Sensitivity analyses to validate the estimated causal effect of T2D on ALS

We performed extensive sensitivity analyses to validate the causal association between T2D and ALS. Due to the nonsignificant associations between T2D-related glycemic/anthropometric traits and ALS, we only summarized these results in Table 3 and Additional file 1: Figure S3, but did not pursue any of these T2D-related traits further. In addition, as explained in the previous section, in addition to using the final set of 67 T2D instrumental variables, we also performed sensitivity analyses using all 139 T2D instrument variables (results shown in Additional file 1: Figure S4). The MR-PRESSO analysis shows that one SNP seems to be an instrumental outlier at the nominal significance level of 0.05 (rs1758632, which was located within gene *UBAP2* and showed a large effect size on ALS with beta = 0.057 and se = 0.014). Removing this instrumental variable does not lead to a substantial change of the causal effect (OR = 0.95, 95% CI 0.89–1.00, *p* = 0.060). The LOO results suggest that no single instrumental variable can influence the estimated causal effect (Additional file 1: Table S11). For example, after removing rs7729395 (located within gene *PAM*), which showed the greatest effect size on both T2D (beta = 0.137, se = 0.016) and ALS (beta = −0.077, se = 0.037) among all instruments, the OR of T2D on ALS is estimated to be 0.94 (95% CI 0.89–1.00, *p* = 0.039). Removing both rs7729395 and rs1758632 leads to a slightly conservative estimate with the OR of T2D on ALS estimated to be 0.95 (95% CI 0.90–1.01, *p* = 0.098). Nevertheless, all these estimates are consistent with the causal effect estimated in the previous section using all the available instrumental variables.

**Table 3:** Summary of the causal effects of T2D and T2D-related glycemic/anthropometric traits on ALS with various MR methods

Exposures (unit)	Random-effects IVW	Weighted median	MR-Egger	
			Odds ratio	Intercept (*p* value)
T2D	0.93 (0.87–1.00)	0.94 (0.86–1.03)	0.90 (0.72–1.14)	0.002 (0.786)
BMI (4.8 kg/m^2^)	1.00 (0.87–1.16)	0.93 (0.77–1.13)	0.80 (0.56–1.15)	0.007 (0.170)
Fasting glucose (mmol/L)	1.09 (0.90–1.31)	1.08 (0.98–1.43)	1.10 (0.97–1.57)	0.001 (0.852)
Fasting insulin (pmol/L)	0.78 (0.49–1.24)	0.66 (0.33–1.31)	1.91 (0.06–61.7)	−0.015 (0.615)
HbA1c (%)	0.97 (0.67–1.39)	0.85 (0.55–1.32)	0.95 (0.47–1.91)	0.001 (0.875)

The weighted median method also yielded a similar point estimate of causal effect of T2D on ALS (OR = 0.94, 95% CI 0.86–1.03, *p* = 0.159). With MR-Egger regression, the OR of T2D on ALS is estimated to be 0.90 (95% CI 0.72–1.14, *p* = 0.390), which is consistent with the main results. Importantly, the intercept of MR-Egger is not significantly deviated from zero (0.002, 95% CI −0.012–0.017, *p* = 0.768), suggesting that no apparent horizontal pleiotropy was detected. In addition, the funnel plot also displays symmetric pattern of effect size variation around the point estimate (Fig. 2b), again indicating no apparent horizontal pleiotropy. To alleviate further concerns with horizontal pleiotropy, we excluded all the 17 instruments that have possible pleiotropic effects (Additional file 1: Table S7) and found that the resulted causal effect estimate remains largely unchanged (OR = 0.93, 95% CI 0.87–0.99, *p* = 0.047). When we excluded ten instruments that were associated with BMI and BMI-related traits (e.g., birth weight, height, waist circumference, waist–hip ratio, and weight), we also obtained a similar causal effect estimate (OR = 0.93, 95% CI 0.88–1.00, *p* = 0.036). Together, these results suggest that horizontal pleiotropy would unlikely bias the estimated causal effect of T2D on ALS. In addition, the causal effect of ALS on T2D is not statistically significant (OR = 1.03, 95% CI 0.94–1.13, *p* = 0.505), ruling out the probability of reverse causation.

**Fig. 3:**
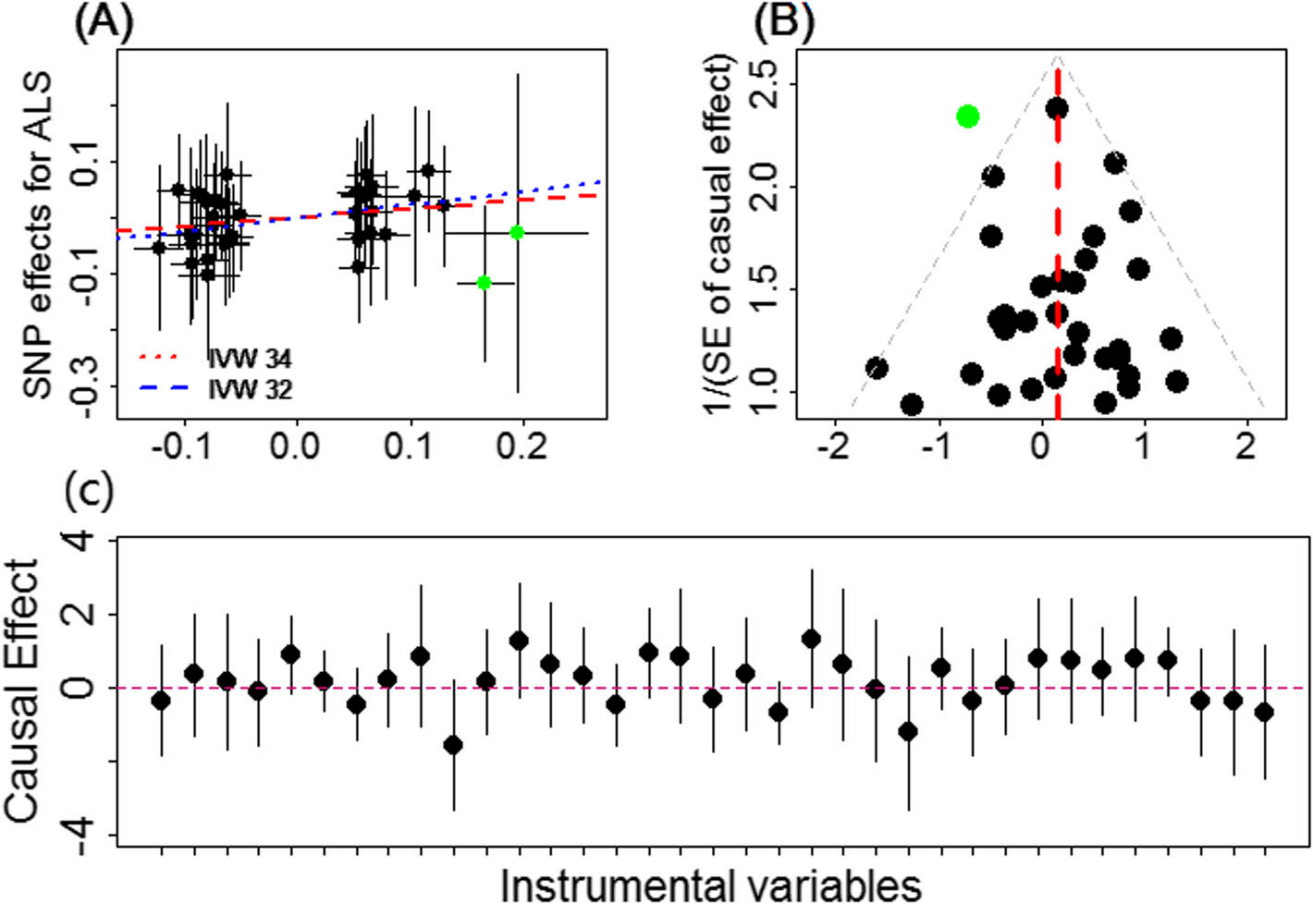
**a** Relationship between the effect size estimates on T2D (*x*-axis) and the effect size estimates on ALS (*y*-axis) for all SNPs that served as instrumental variables for T2D in the East Asian population. The 95% confidence intervals for the estimated SNP effect sizes on ALS are shown as vertical black lines, while the 95% confidence intervals for the estimated SNP effect sizes on T2D are shown as horizontal black lines. The slope of fitted lines represents the estimated the causal effect of T2D on ALS obtained with all the 34 instrumental variables (red lines) or with only 32 instrumental variables (blue lines; two potential outliers, rs12219514 and rs75536691, are highlighted in green and are removed). **b** Funnel plot displays individual causal effect estimates for T2D on ALS in the East Asian population. The dots represent the estimated causal effect for each instrumental variable. The vertical red line represents the estimated causal effect obtained using all instrumental variables. A possible outlier (i.e., rs12219514) is highlighted in green. **c** Forest plot for individual causal effect estimate

Finally, we investigated the causal relationship between T2D and ALS in the East Asian population. Due to the much smaller sample size of ALS (Table 1), the estimated causal effect of T2D on ALS in the East Asian population is not statistically significant at the level of 0.05. Specifically, the OR of T2D on ALS is estimated to be 1.17 (95% CI 0.93–1.47, *p* = 0.190). After removing two potential outliers (i.e., rs12219514 and rs75536691), however, the causal effect of T2D on ALS becomes stronger and marginally statistically significant (OR = 1.28, 95% CI 0.99–1.62, *p* = 0.058) (Fig. 3a), suggesting that T2D may increase the risk of ALS in the East Asian population. Note that the positive causal effect of T2D on ALS in the East Asian population is in contrast with the negative causal effect of T2D on ALS estimated in the European population. Our sensitivity analyses further support the conclusion in the East Asian population. For example, the funnel plot displays a symmetric pattern of effect size variation around the point estimate (Fig. 3b), and the result of MR-Egger regression also rules out the possible influence of horizontal pleiotropy in our analysis (e.g., the MR-Egger intercept is estimated to be 0.021, with 95% CI −0.039–0.081, *p* = 0.488).

## Discussion

### Summary of the results of the present study

It has long been controversial whether T2D is causally protective against ALS or whether ALS is causally protective against T2D [10, 11]. Here, we have carried out a comprehensive two-sample MR analysis to clarify this controversial issue. To the best of our knowledge, our study is the first exploration that attempts to illuminate the directional causal relationship between T2D and ALS using a genetic approach by leveraging summary statistics from large-scale GWASs. Our results support a neuroprotective role of T2D on ALS in the European population.

As the validity of the MR analysis depends on strong model assumptions, we have carefully selected instrumental variables for T2D in order to satisfy those assumptions. We have conducted extensive sensitivity analyses in the present study to guard against various possible model misspecifications. Given the pervasive pleiotropy among complex traits [73], we attempted to remove instrumental variables with potential pleiotropic effects (Fig. 1 and Additional file 1: Table S7). To quantify the genetic covariance between T2D and ALS, we applied the linkage disequilibrium score regression (LDSC) [74]—a novel approach that can make full use of the genome-wide pleiotropy. With LDSC, consistent with what has been observed in a recent study [52], we found no genetic correlation between T2D and ALS in the European population (*R*_g_ = −0.011, se = 0.020, *p* = 0.581) or in the East Asian population (*R*_g_ = 0.051, se = 0.071, *p* = 0.469). The LDSC results suggest a lack of polygenic pleiotropy between T2D and ALS, supporting the issue that pleiotropic instrumental variables unlikely exist in our MR study. These observations are further supported by the distinct pattern of Manhattan plots for T2D and ALS in the two populations (Additional file 1: Figure S5). Therefore, we believe that the employed index SNPs of T2D in our analyses are valid instrumental variables that adequately satisfy the model assumptions required by MR.

One of the advantages of our study is the large-scale GWASs we used, where the large sample sizes allow us to fully establish a credible causal relationship between T2D and ALS in the European population. Importantly, the inferred causal relationship between T2D and ALS is robust with respect to the choice of statistical methods and is carefully validated through various sensitivity analyses and multiple sets of instruments. Overall, our study provides new evidence supporting the causal role of T2D on decreasing the risk of ALS in the European population. Therefore, given that little has been understood about the risk factors for ALS to date [8], our study is an important addition to the line of ALS research and has an important implication for public health. Our results shed light on the pathology of ALS and have the potential to pave the way towards new therapeutic strategies for ALS [75].

### Various associations between T2D and ALS in the East Asian and European populations

Our causal association results are consistent with the association results obtained in several previous observational epidemiological studies (Table 1 and Additional file 1: Table S1). For example, negative associations between T2D and the risk of ALS were consistently observed in the European population [11, 14, 16, 19]. However, we note that the neuroprotective association between T2D and ALS in the European population does not appear to generalize to the East Asian population. Indeed, our MR analysis provides empirically suggestive evidence that T2D might instead increase the risk of ALS in the East Asian population (OR = 1.17 or 1.28), which is in line with a previous East Asian study (Table 1 and Additional file 1: Table S1) [22].

**Fig. 4:**
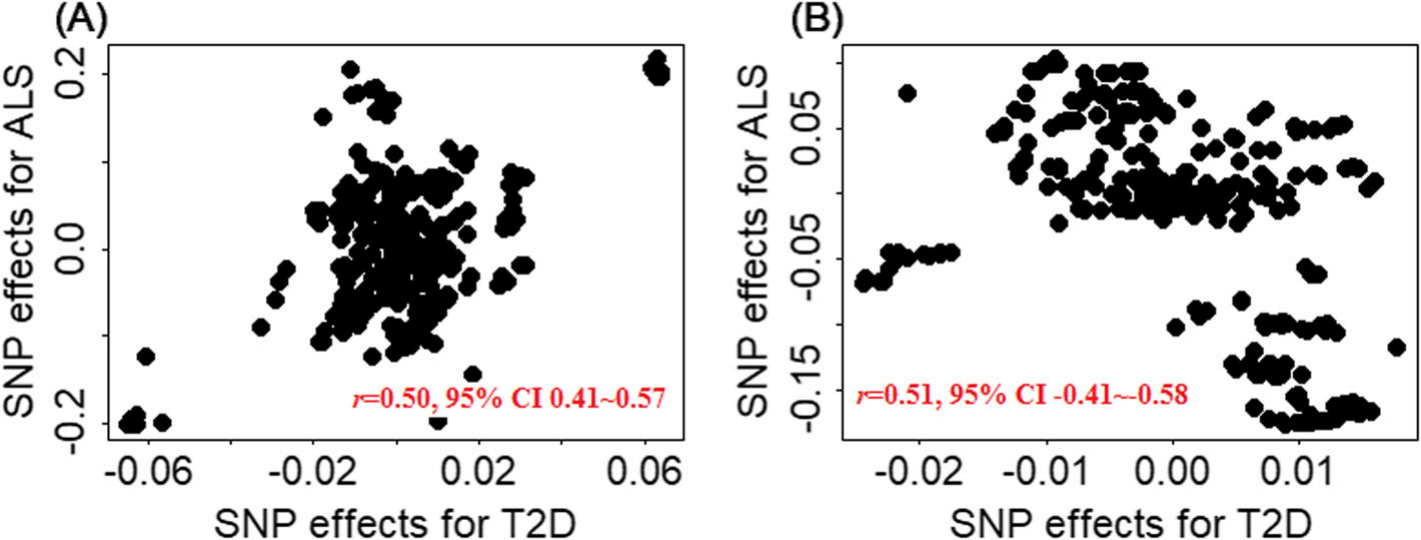
Pearson’s product-moment correlation between the effect size estimates on T2D (*x*-axis) and the effect size estimates on ALS (*y*-axis) for all SNPs that were located in gene *C9orf72* in the East Asian (**a**) and European (**b**) populations. Defined by GENCODE (version 19) [80], a total of 339 SNPs located within *C9orf72* (Chr 9: 27,446,544~27,673,864) were included in the two correlation plots

Given the substantial difference of ALS in terms of clinical features and potential molecular mechanisms between European and East Asian populations, our finding does not come as a surprise. First, the pathophysiology of ALS differs in patients of different ethnicity [27, 63]. It has been well characterized that the risk of developing ALS in East Asia is much lower than that in Europe [76]; the mean age of ALS onset in East Asia is also significantly earlier than that in other countries [77]. Therefore, ALS in the two populations may be heterogenous and influenced by different biological pathways. It is thus possible that T2D affects different biological pathways underlying ALS in different pupations, leading to different observed effects on ALS in the two populations. Second, the genetic architecture underlying ALS may be distinct in the two populations [63, 78, 79]. For example, the expansion of *C9orf72*—the most common ALS associated gene in the European population—has a much lower frequency in the East Asian ALS patients (0.30% vs. 7.00%). In addition, we found that SNPs located in *C9orf72* of T2D patients are significantly positively associated with ALS in the East Asian population (Fig. 4a: *r* = 0.50, 95% CI 0.41–0.57, *p* = 5.41E−26) but are significantly negatively associated with ALS in the European population (Fig. 4b: *r* = 0.51, 95% CI −0.41–−0.58, *p* = 5.99E−27). Subsequently, T2D may influence a genetic pathway that has the opposite effects on ALS in the two populations [63, 81, 82]. Indeed, we found that the genetic covariances between T2D and ALS estimated through LDSC analysis also have opposite directions in the two populations: T2D and ALS are negatively genetically correlated in the European population (*R*_g_ = −0.011) but positively genetically correlated in the East Asian population (*R*_g_ = 0.051). Finally, different genetic architecture, different environment exposure, and ALS disease heterogeneity may interact with each other to lead to different T2D effects on ALS in the European and East Asian populations [14]. Therefore, the biological mechanism underlying the different effects of T2D on ALS across populations can be multifactorial and warrant future investigations.

Certainly, we cannot exclude the possibility that the nonsignificant positive association between T2D and ALS in the East Asian population may be simply due to a lack of power there, as the sample size of ALS in the East Asian population is much smaller compared with that in the European population (1234/2850 vs. 20,806/59,804) (Table 2). We calculated power using the analytic method developed in [83] (https://cnsgenomics.shinyapps.io/mRnd/), by assuming that the true causal OR of T2D on ALS is 1.20 (or equivalently 0.80) (approximately equal to that estimated in the East Asian population), PVE = 0.01 (approximately equal to that estimated in terms of the used instrumental variables), the significance level *α* is 0.05, and the proportion of ALS cases is the same as that in the respective ALS GWASs. With this analytic approach, we estimated the statistical power of the two-sample MR detecting such causal effect to be only 9% in the East Asian population; this is in contrast to an estimated statistical power of 72% in the European population.

Nevertheless, our findings suggest that a different mechanism may underlie the association between T2D and ALS in the two populations, which is important for understanding the ALS pathology and developing its clinical treatment in different countries. For example, some drug interventions that are effective in the European ALS patients may not necessarily work for East Asian individuals or vice versa. Further investigations on this important issue are warranted in the future.

### Other new contributions from the present study

Besides revealing the neuroprotective role of T2D for ALS in the European population and providing suggestive evidence of increasing the risk of ALS in the East Asian population, our study also, at least in part, solves several previously unanswered questions [75]. First, we demonstrated that, except for T2D itself, no common T2D-related exposures investigated in the present study showed a causal relationship with ALS. These results suggest that those T2D-related exposures might not mediate the influence of T2D on ALS and that T2D is causally protective against ALS via some other unknown pathways. Therefore, intervening fasting glucose and insulin in T2D patients might not impact the risk of ALS or slow its disease progression. Furthermore, previous studies have showed that T2D is positively related to Parkinson’s disease [84, 85] but is not associated with Alzheimer’s disease [57, 86]. Therefore, the lack of association between T2D and Alzheimer’s disease, together with the positive association between T2D and Parkinson’s disease, imply that the effect of T2D on the motor neurons may be selectively protective and is special for ALS.

### Mechanisms underlying the causal associations between T2D and ALS

We note that the causal relationship between T2D and ALS identified in the present study does not necessarily imply that T2D itself directly affects ALS. Instead, pathways associated with T2D may affect the risk of ALS, or, alternatively, T2D may affect ALS through indirectly pathways. Indeed, the mechanisms underlying the causal associations between T2D and ALS may be considerably complex. Several explanations for such causal association exist. For example, it is well known that T2D is associated with higher blood lipids [87], which could resist against the increased energy consumption and hypermetabolism of ALS patients. Therefore, T2D may act through blood lipids to reduce the hypermetabolic damage on the motor neuron system [88], potentially delaying ALS onset and increasing the survival time of ALS patients [12, 89–92]. As another explanation, T2D was recently reported to be associated with higher concentrations of progranulin [93], which could mediate fat-induced insulin resistance and revert mutant TDP-43 (TAR DNA-binding protein 43)-induced axonopathy [75, 94]. Therefore, T2D may act through TDP-43, which is a well-known risk factor of ALS [8, 95, 96]. As a third explanation, medical treatments and reverted lifestyle (e.g., smoking cessation) for T2D patients may be also beneficial to reduce the risk of ALS.

### Some limitations

Some limitations of this study should be considered. First, similar to other MR studies, we acknowledge that the validity of our MR relies on some crucial modeling assumptions (Additional file 1: Figure S2A) [30, 31, 40], some of which cannot be fully tested for in the framework of two-sample MR. In addition, we also acknowledge that MR cannot fully rule out all the confounders even though MR is less susceptible to reverse causation and confounders compared with other study designs [97]. For example, we cannot completely rule out the indirect role of BMI in the causal relation between T2D and ALS, as it was shown that the nutritional intervention for ALS patients to raise BMI can prolong the survival time and result in the delay of disease progression [90–92] and increasing BMI is directly related to the risk of T2D. Thus, we emphasize that the results generated in the present study should be interpreted with caution, even though we have been extremely careful in selecting instruments to satisfy various model assumptions and have conducted extensive sensitivity analyses to guard against model assumption misspecifications.

Second, as in other previous MR studies, we assumed a linear effect relationship between T2D and ALS in the MR model. While linearity is a first-order approximation of any nonlinear relationship, a simple linearity assumption may not always be reasonable in practice. Indeed, we cannot fully rule out the possibility of nonlinear association between T2D and ALS. In addition, no data on the severity and duration of T2D are available. Therefore, similar to other MR analyses, we cannot assess the dose–response relationship between T2D and ALS, which is an important aspect of causal inference.

Third, previous studies have shown that the impact of T2D on ALS can be age (and/or gender) dependent [16, 19, 22]. For example, some studies have identified a protective effect of T2D on ALS in old people while identified zero effect or adverse effect in younger ones. As another example, some studies have shown that men may have a higher risk than women in East Asian individuals [98, 99]. Unfortunately, because it is almost impossible to obtain individual-level data in GWAS due to privacy concerns, we cannot directly estimate the causal effect between T2D and ALS stratified by gender or age groups.

Fourth, the clinical heterogeneity of ALS was recognized in previous studies [100] and some studies have implied that T2D may have a stronger protective effect in bulbar ALS compared with spinal ALS [14]. In the present study, unfortunately, we are unable to further investigate the effect of T2D on sub-phenotypes of ALS due to lack of individual-level information.

Fifth, likely due to the relatively small sample size for ALS cases and the relatively weak exposure effects on the outcome, the statistical power of our MR analysis is limited for certain exposures such as glycemic traits. For example, when the true causal OR is 0.90 (approximately equal to the estimates in the present study), the statistical power to detect a causal effect of fasting insulin (or hemoglobin A1c) on ALS is only 14% (or 43%) with an analytic method proposed in [83]. Note that the calculated power is smaller than that for T2D in the European population because a weaker true OR is assumed here (0.90 vs. 0.80). Certainly, besides the lack of power and the absence of true causal effect, other possible explanations, such as potential unmodeled nonlinear effect, unidentified reverse causality, shared genetic contribution, and uncontrolled confounding effect, may all contribute to the potential failure to detect the association between glycemic traits and ALS. Therefore, larger longitudinal studies with individual-level data in the future are needed to fully establish the causal relationship between those glycemic traits and ALS. These future studies can help elucidate the potential mediating role of glycemic traits in the T2D effects on ALS.

## Conclusion

In conclusion, based on the MR results obtained from large-scale GWAS summary statistics, our study provides new evidence on the causal neuroprotective role of T2D on the risk of ALS in the European population as well as empirically suggestive evidence of increasing risk of T2D on ALS in the East Asian population.

## Supplementary information

**Additional file 1.** Supplementary Figures and Tables.

**Additional file 2.** An Overview of the Mendelian Randomization Method.

**Additional file 3.** Description of GWAS Data Sets.

## Data Availability

All data generated or analysed during this study are included in this published article and its supplementary information files.

## Abbreviations

ALS: Amyotrophic lateral sclerosis
GWAS: Genome-wide association study
IVW: Inverse-variance weighted
LOO: Leave-one-out
MR: Mendelian randomization
MR-PRESSO: Mendelian Randomization Pleiotropy RESidual Sum and Outlier
OR: Odds ratio
PVE: Phenotypic variance explained
SNP: Single-nucleotide polymorphism
T2D: Type 2 diabetes
