# Supplementary material for: Causal association of type 2 diabetes with amyotrophic lateral sclerosis: new evidence from Mendelian randomization using GWAS summary statistics
Source: BMC Med. 2019 Dec 4;17:225. doi: 10.1186/s12916-019-1448-9 (PMC6892209; doi:10.1186/s12916-019-1448-9)
Supplement: Supplementary file 1 — Additional file 1. Supplementary Figures and Tables. [file 12916_2019_1448_MOESM1_ESM.doc]

**Additional file 1**

# Supplementary Figures and Tables

(A)


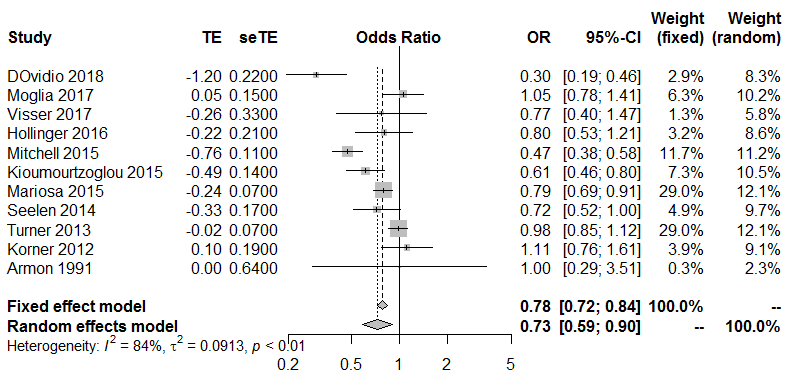


(B)


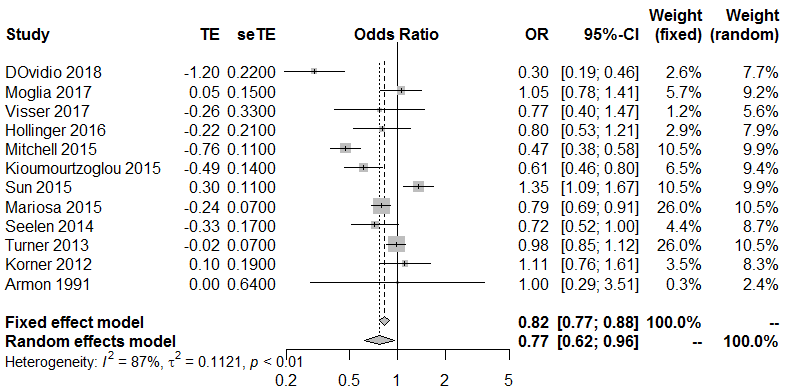


Figure S1 Combined OR of T2D on ALS in the meta-analysis (A) without the study of [Sun, et al.](#_ENREF_16) and (B) with all the studies available. OR: odds ratio; T2D: type 2 diabetes; ALS: amyotrophic lateral sclerosis.

(A)


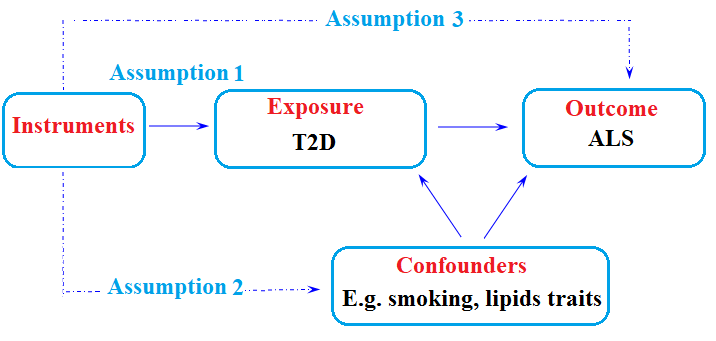


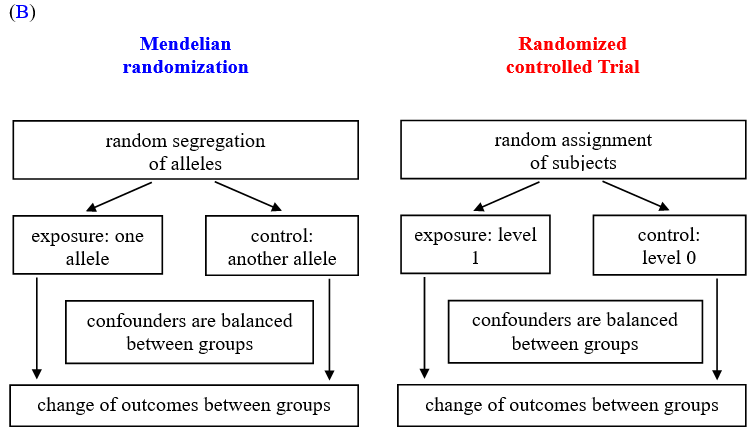


Figure S2 (A) Graphical illustration of Mendelian randomization analysis shows three key model assumptions (i.e. the relevance assumption, the independence assumption and the exclusion restriction assumption). In the plot solid arrows or dotted arrows denote the presence or absence of directional associations. (B) Comparison between Mendelian randomization and randomized controlled trial. T2D: type 2 diabetes; ALS: amyotrophic lateral sclerosis.


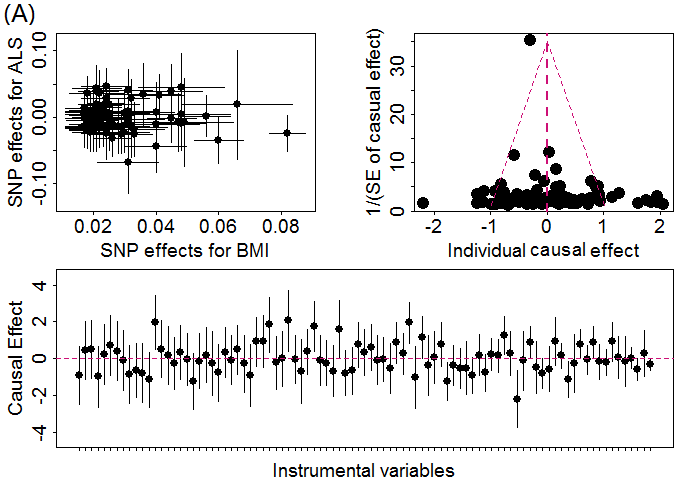


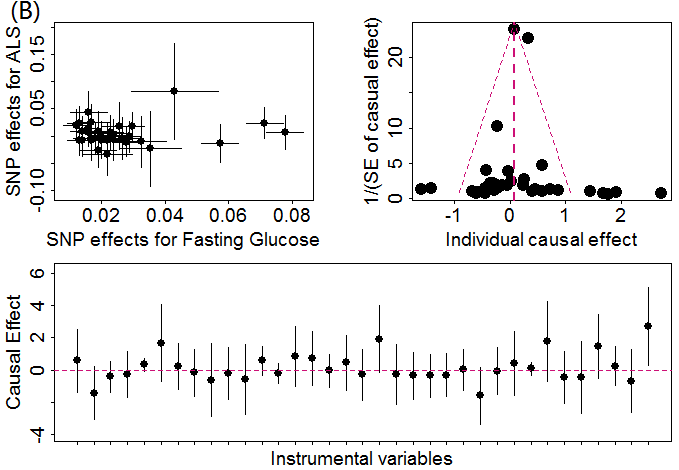


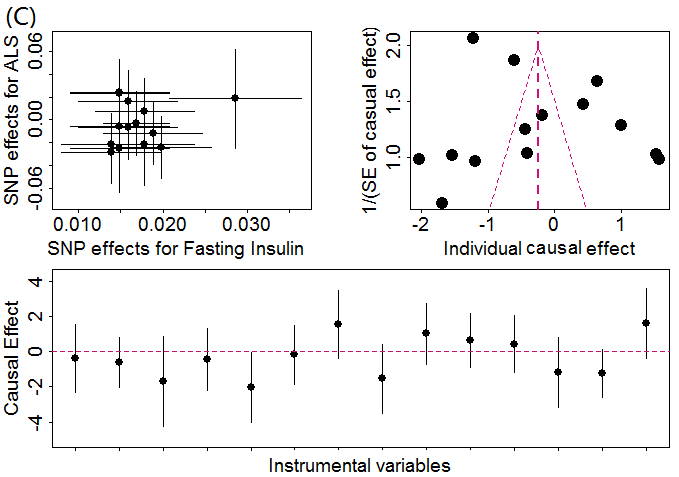


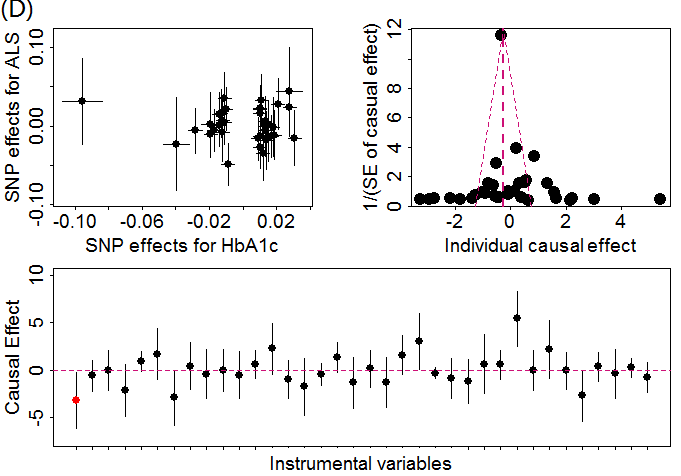


Figure S3 Sensitivity results for the relationship between four T2D-related exposures (A: body mass index, BMI; B: fasting glucose; C: fasting insulin; D: HbA1c) and ALS. In each panel, the first one is the relationship between the effect size estimates on the exposure and the effect size estimates on ALS (y-axis) for all SNPs that are served as instrumental variables in the European population. The 95% confidence intervals for the estimated SNP effect sizes on ALS are shown as vertical black lines, while the 95% confidence intervals for the estimated SNP effect sizes on the exposure are shown as horizontal black lines. The second and the third are the funnel plot and the forest plot for individual causal effect estimate for an exposure on ALS. For BMI, one instrument (i.e. rs1558902) can be considered outlier; removing this instrument cannot substantially change the estimated causal effect (OR = 1.05, 95% CI 0.90 - 1.22, *p* = 0.528). For fasting glucose, removing four potential instrument outliers (i.e. rs560887, rs730497, rs16913693 and rs10830963) does not lead to substantially change the estimated causal effect (OR = 1.00, 95% CI 0.77 - 1.32, *p* = 0.958). For fasting insulin, removing one potential instrument outlier (i.e. rs983309) does not result in substantially change the estimated causal effect (OR = 0.71, 95% CI 0.39 - 1.29, *p* = 0.259). For HbA1c, removing one potential instrument outlier (i.e. rs4745982) cannot substantially alter the estimated causal effect (OR = 1.07, 95% CI 0.70 - 1.62, *p* = 0.762). ALS: amyotrophic lateral sclerosis; HbA1c: hemoglobin A1c; OR: odds ratio.


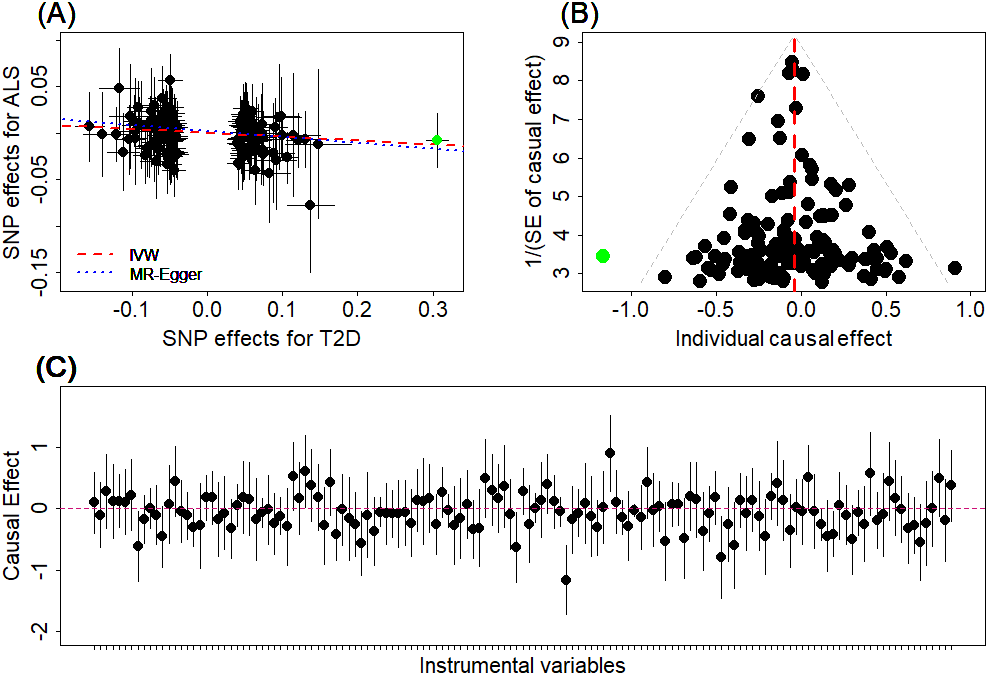


Figure S4 (A) Relationship between the effect size estimates on T2D (x-axis) and the effect size estimates on ALS (y-axis) for all SNPs that are served as instrumental variables for T2D in the European population. Here, a total of 139 T2D instrumental variables were employed. The 95% confidence intervals for the estimated SNP effect sizes on ALS are shown as vertical black lines, while the 95% confidence intervals for the estimated SNP effect sizes on T2D are shown as horizontal black lines. The slope of fitted lines represents the estimated causal effect of T2D on ALS obtained using either the IVW method (red lines) or the MR-Egger regression (blue lines). One possible SNP outlier (i.e. rs7903146) is highlighted in green. (B) Funnel plot displays individual causal effect estimates for T2D on ALS in the European population. The dots represent the estimated causal effect for each instrumental variable. The vertical dotted red line represents the estimated causal effect obtained using all instrumental variables. A possible outlier (i.e. rs1758632) is highlighted in green. (C) Forest plot for individual causal effect estimate. The causal effect of T2D on ALS is estimated to be 0.96 (95% CI 0.92 - 1.00, *p* = 0.046). With MR-Egger regression, the OR of T2D on ALS is estimated to be 0.94 (95% CI 0.86 - 1.03, *p* = 0.174). The intercept of MR-Egger is not significantly deviated from zero (0.002, 95% CI -0.005 - 0.008, *p* = 0.584). The removal of one outlier (i.e. rs7903146) leads to a similar causal effect estimate (OR = 0.96, 95% CI 0.92 - 1.00, *p* = 0.050). T2D: type 2 diabetes; ALS: amyotrophic lateral sclerosis.


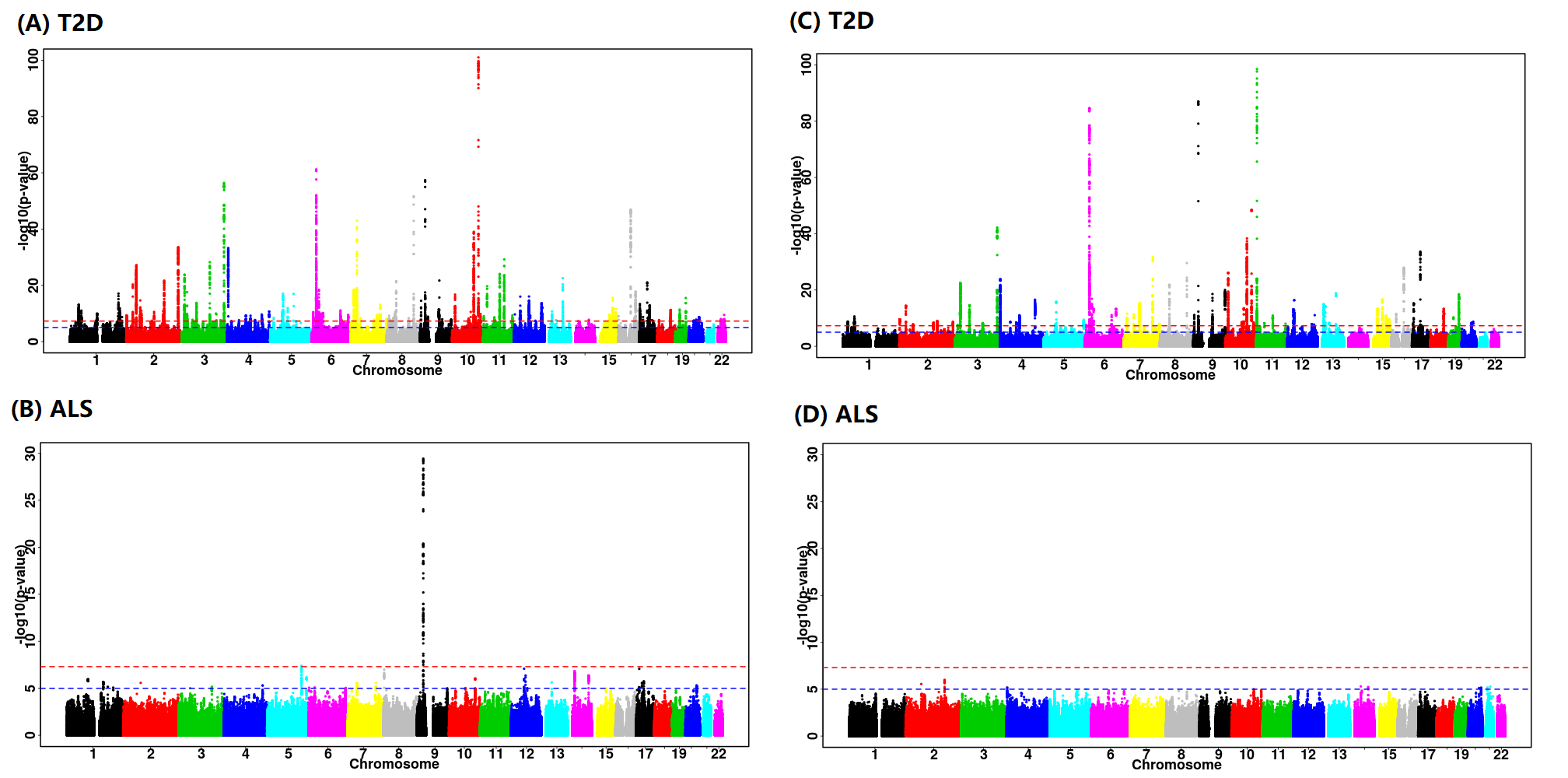


Figure S5 Manhattan plots for T2D and ALS in in the European (A and B) and East Asian (C and D) populations. GWAS summary statistics results for T2D were publicly available at <http://cnsgenomics.com/data.html> and http://jenger.riken.jp/en/result; GWAS summary statistics results for ALS were publicly available at <http://als.umassmed.edu/> and <http://cnsgenomics.com/data/benyamin_et_al_2017_nc>. ALS: amyotrophic lateral sclerosis; T2D: type 2 diabetes.

Table S1 Detailed summary of the estimated effect sizes of T2D on ALS in previous observational studies

| OR (95% CI) | Sub-group | Age | Follow-up | Statistical methods | Covariates | Cases | Stratified analysis | Nations | N | Design | Ref |
| --- | --- | --- | --- | --- | --- | --- | --- | --- | --- | --- | --- |
| 0.30 (0.19-0.45) | Ever exposure to diabetes, adjusted for gender, age, educational level and marital status | Over 14 years at 1996 | 1998~2014 | Cox PH model | Age, gender, education and marital status | 397 | Gender/age or ALS phenotype | Turin in Italy | 727,977 | Retrospective cohort |  |
| 0.34 (0.20-0.55) | Male, ever exposure to diabetes, adjusted for gender, age, educational level and marital status |  |  |  |  |  |  |  |  |  |  |
| 0.23 (0.11-0.50) | Female, ever exposure to diabetes, adjusted for gender, age, educational level and marital status |  |  |  |  |  |  |  |  |  |  |
| 0.30 (0.14-0.64) | 50~64 years, ever exposure to diabetes, adjusted for gender, age, educational level and marital status |  |  |  |  |  |  |  |  |  |  |
| 0.30 (0.18-0.50) | >65 years, ever exposure to diabetes, adjusted for gender, age, educational level and marital status |  |  |  |  |  |  |  |  |  |  |
| 0.29 (0.19-0.43) | ever exposure to diabetes, adjusted for gender and age |  |  |  |  |  |  |  |  |  |  |
| 0.32 (0.20-0.53) | Male, ever exposure to diabetes, adjusted for gender and age |  |  |  |  |  |  |  |  |  |  |
| 0.23 (0.11-0.49) | Female, ever exposure to diabetes, adjusted for gender and age |  |  |  |  |  |  |  |  |  |  |
| 0.29 (0.13-0.62) | 50~64 years, ever exposure to diabetes, adjusted for gender and age |  |  |  |  |  |  |  |  |  |  |
| 0.30 (0.18-0.49) | >65 years, ever exposure to diabetes, adjusted for gender and age |  |  |  |  |  |  |  |  |  |  |
| 0.19 (0.07-0.46) | bulbar |  |  |  |  |  |  |  |  |  |  |
| 0.35 (0.22-0.56) | spinal |  |  |  |  |  |  |  |  |  |  |
| 0.26 (0.17-0.40) | <4 after diabetes diagnosis |  |  |  |  |  |  |  |  |  |  |
| 0.26 (0.17-0.41) | 5~11 after diabetes diagnosis |  |  |  |  |  |  |  |  |  |  |
| 0.29 (0.19-0.45) | >12 after diabetes diagnosis |  |  |  |  |  |  |  |  |  |  |
| 0.29 (0.16-0.54) | Shorter diagnostic delay |  |  |  |  |  |  |  |  |  |  |
| 0.30 (0.17-0.53) | Long diagnostic delay |  |  |  |  |  |  |  |  |  |  |
| 1.02 (0.88-1.17) | Unadjusted |  |  |  |  |  |  |  |  |  |  |
| 1.05 (0.91-1.22) | Age-adjusted |  |  |  |  |  |  |  |  |  |  |
| 1.05 (0.78-1.42) | Multivariable adjusted |  |  |  |  |  |  |  |  |  |  |
| 0.77 (0.33-1.21) |  |  |  |  |  |  |  | Netherlands | 1,425 |  |  |
| 0.80 (0.53-1.21) | Age of onset | 60.1±12.5 |  | Ordinal logistic regression | Age, gender, race and other antecedent diseases except diabetes | 1,439 |  | USA | 1,439 | history surveys |  |
| 1.18 (0.70-2.00) | Disease duration |  |  |  |  |  |  |  | 787 | history surveys |  |
| 0.47 (0.38-0.58) | diabetes | >20 years |  | standard odds ratio (OR) statistical test | Age, gender, geography | 1,288 |  | UAS | 8,849 | Case-control |  |
| 0.61 (0.46-0.80) | Adjusted for socioeconomic status, prior chronic obstructive pulmonary disease, marital status, and residence at amyotrophic lateral sclerosis diagnosis, with diabetes and obesity simultaneously included in the model. | 65.4±11.6 |  | Conditional logistic regression | COPD, marital status, and region of residence, gender and age, socioeconomic status | 3,650 | Interaction between diabetes and gender or age; early and late first hospital discharge codes | Danish | 368650 | Case-control |  |
| 0.59 (0.45-0.77) | Unadjusted |  |  |  |  |  |  |  | 368650 | Case-control |  |
| 0.59 (0.45-0.77) | Adjusted for socioeconomic status, prior chronic obstructive pulmonary disease, marital status, and residence at amyotrophic lateral sclerosis diagnosis. |  |  |  |  |  |  |  | 368650 | Case-control |  |
| 1.66 (0.85-3.21) | Age < 40 years |  |  |  |  |  |  |  |  |  |  |
| 0.52 (0.39-0.70) | Age > 40 years |  |  |  |  |  |  |  |  |  |  |
| 0.66 (0.50-0.88) | Exclude diabetes in the five years prior to the index data |  |  |  |  |  |  |  |  |  |  |
| 0.69 (0.50-0.96) | Exclude diabetes in the seven years prior to the index data |  |  |  |  |  |  |  |  |  |  |
| 0.61 (0.46-0.80) | Adjusted by obesity |  |  |  |  |  |  |  |  |  |  |
| 1.17 (0.84-1.64) | women, adjusted |  |  |  |  |  |  |  |  |  |  |
| 1.35 (1.10-1.67) | overall, adjusted | 60.10±12.73 for case, 60.0±12.84 for control | 2000-2008 | Cox PH | sex, age, geographic area, urbanization status, Charlson Comorbidity Index, frequency of medical visit, and histories of hypertension, hyperlipidemia, and chronic obstructive pulmonary disease | 456 | Age and gender | China | 12,303,27 | cohort |  |
| 1.70 (1.26-2.30) | Men, ≤65, crude |  |  |  |  |  |  |  |  |  |  |
| 1.13 (0.77-1.65) | Men, >65, crude |  |  |  |  |  |  |  |  |  |  |
| 1.45 (1.15-1.84) | Men, crude |  |  |  |  |  |  |  |  |  |  |
| 1.55 (1.04-2.32) | women, ≤65, crude |  |  |  |  |  |  |  |  |  |  |
| 0.93 (0.59-1.47) | women, >65, crude |  |  |  |  |  |  |  |  |  |  |
| 1.24 (0.92-1.67) | women, crude |  |  |  |  |  |  |  |  |  |  |
| 1.37 (1.14-1.64) | overall, crude |  |  |  |  |  |  |  |  |  |  |
| 0.77 (0.66-0.89) | adjusted for age, sex and area of residence |  |  |  |  |  |  |  |  | Case-control |  |
| 2.90 (1.31-6.44) | <50 years, adjusted for age, sex and area of residence |  |  |  |  |  |  |  |  | Case-control |  |
| 1.20 (0.74-1.94) | 50~59 years, adjusted for age, sex and area of residence |  |  |  |  |  |  |  |  | Case-control |  |
| 0.96 (0.72-1.27) | 60~69 years, adjusted for age, sex and area of residence |  |  |  |  |  |  |  |  | Case-control |  |
| 0.71 (0.57-0.89) | 70~79 years, adjusted for age, sex and area of residence |  |  |  |  |  |  |  |  | Case-control |  |
| 0.52 (0.37-0.72) | >80 years, adjusted for age, sex and area of residence |  |  |  |  |  |  |  |  | Case-control |  |
| 0.79 (0.66-0.95) | Men, adjusted for age, sex and area of residence |  |  |  |  |  |  |  |  | Case-control |  |
| 0.73 (0.57-0.93) | women, adjusted for age, sex and area of residence |  |  |  |  |  |  |  |  | Case-control |  |
| 0.73 (0.55-0.97) | 1991-2000, adjusted for age, sex and area of residence |  |  |  |  |  |  |  |  | Case-control |  |
| 0.78 (0.66-0.92) | 2001-2010, adjusted for age, sex and area of residence |  |  |  |  |  |  |  |  | Case-control |  |
| 0.79 (0.68-0.91) | adjusted for age, sex, area of residence, education and socioeconomic status | 68.2±12.2 |  | Conditional logistic regression | age, sex, area of residence, education and socioeconomic status | 5,108 | Age, gender and calendar period | Sweden | 30,648 | Nested case-control |  |
| 3.15 (1.40-7.08) | <50 years, adjusted for age, sex, area of residence, education and socioeconomic status |  |  |  |  |  |  |  |  | Case-control |  |
| 1.20 (0.74-1.95) | 50~59 years, adjusted for age, sex, area of residence, education and socioeconomic status |  |  |  |  |  |  |  |  | Case-control |  |
| 0.95 (0.71-1.27) | 60~69 years, adjusted for age, sex, area of residence, education and socioeconomic status |  |  |  |  |  |  |  |  | Case-control |  |
| 0.71 (0.57-0.89) | 70~79 years, adjusted for age, sex, area of residence, education and socioeconomic status |  |  |  |  |  |  |  |  | Case-control |  |
| 0.56 (0.40-0.78) | >80 years, adjusted for age, sex, area of residence, education and socioeconomic status |  |  |  |  |  |  |  |  | Case-control |  |
| 0.80 (0.67-0.96) | Men, adjusted for age, sex, area of residence, education and socioeconomic status |  |  |  |  |  |  |  |  | Case-control |  |
| 0.77 (0.60-0.98) | women, adjusted for age, sex, area of residence, education and socioeconomic status |  |  |  |  |  |  |  |  | Case-control |  |
| 0.77 (0.58-1.04) | 1991-2000, adjusted for age, sex, area of residence, education and socioeconomic status |  |  |  |  |  |  |  |  | Case-control |  |
| 0.79 (0.67-0.93) | 2001-2010, adjusted for age, sex, area of residence, education and socioeconomic status |  |  |  |  |  |  |  |  | Case-control |  |
| 0.83 (0.60-1.15) | insulin-dependent diabetes |  |  |  |  |  |  |  |  | Case-control |  |
| 5.38 (1.87-15.51) | <50 years and insulin-dependent diabetes |  |  |  |  |  |  |  |  | Case-control |  |
| 1.16 (0.54-2.51) | 50~59 years and insulin-dependent diabetes |  |  |  |  |  |  |  |  | Case-control |  |
| 1.19 (0.65-2.19) | 60~69 years and insulin-dependent diabetes |  |  |  |  |  |  |  |  | Case-control |  |
| 0.45 (0.25-0.82) | 70~79 years and insulin-dependent diabetes |  |  |  |  |  |  |  |  | Case-control |  |
| 0.46 (0.14-1.51) | >80 years and insulin-dependent diabetes |  |  |  |  |  |  |  |  | Case-control |  |
| 0.66 (0.53-0.81) | non-insulin-dependent diabetes |  |  |  |  |  |  |  |  | Case-control |  |
| 2.12 (0.37-12.10) | <50 years and non-insulin-dependent diabetes |  |  |  |  |  |  |  |  | Case-control |  |
| 0.77 (0.32-1.83) | 50~59 years and non-insulin-dependent diabetes |  |  |  |  |  |  |  |  | Case-control |  |
| 0.74 (0.49-1.12) | 60~69 years and non-insulin-dependent diabetes |  |  |  |  |  |  |  |  | Case-control |  |
| 0.67 (0.49-0.91) | 70~79 years and non-insulin-dependent diabetes |  |  |  |  |  |  |  |  | Case-control |  |
| 0.51 (0.33-0.80) | >80 years and non-insulin-dependent diabetes |  |  |  |  |  |  |  |  | Case-control |  |
| 0.70 (0.57-0.86) | Index dates during 2006~2010 |  |  |  |  |  |  |  |  |  |  |
| 0.72 (0.55-0.94) | Index dates during 2006~2010, non-insulin-dependent diabetes |  |  |  |  |  |  |  |  |  |  |
| 1.36 (0.78-2.37) | Index dates during 2006~2010, insulin-dependent diabetes |  |  |  |  |  |  |  |  |  |  |
| 3.25 (1.61-6.53) | Diabetes ascertained at age <30 |  |  |  |  |  |  |  |  |  |  |
| 0.74 (0.63-0.85) | Diabetes ascertained at age >30 years |  |  |  |  |  |  |  |  |  |  |
| 0.66 (0.54-0.80) | diabetes diagnosed 3 or more years before the index date |  |  |  |  |  |  |  |  |  |  |
| 0.72 (0.51-1.01) |  | 62.8 (56.7-69.6) for cases and 63.1 (57.3-69.9) for controls |  | logistic regression | Age, gender, education, current smoking and alcohol consumption | 722 | With or without C9orf72 | Netherlands | 2,990 | Case-control |  |
| 0.98 (0.85-1.13) | Insulin-dependent diabetes for all age groups |  |  |  |  | 216 |  | England |  | Cohort |  |
| 3.94 (1.84-7.50) | Insulin-dependent diabetes aged less than 30 years |  |  |  |  |  |  |  |  |  |  |
| 1.11 (0.76-1.60) | survival | 58.8 |  | Cox PH | comorbidities | 514 |  | Germany | 514 | Cohort |  |
| 1.00 (0.29-3.50) | diabetes | 68 (49-89) |  | logistic regression | sex, year of birth, period of observation and residence |  |  | USA· | 135 | Case-control |  |
| 1.07 (0.74-1.57) | progression |  |  |  |  |  |  |  | 516 | Cohort |  |

Table S2 Summary information for instrumental variables of type 2 diabetes in the European population

| Chr | SNP | Position | Gene | ALLELE | BETA | SE | *P* | *N* | *PVE* | *F* |
| --- | --- | --- | --- | --- | --- | --- | --- | --- | --- | --- |
| 9 | rs17791483 | 81,898,980 | *CHCHD2P9* | G/A | -0.102 | 0.015 | 3.42E-12 | 659,316 | 7.30E-05 | 48.1 |
| 10 | rs7923866 | 94,482,076 | *Y_RNA* | T/C | -0.097 | 0.007 | 9.34E-40 | 659,316 | 2.62E-04 | 172.5 |
| 2 | rs2972144 | 227,101,411 | *AC068138.1* | A/G | -0.091 | 0.008 | 2.55E-34 | 659,316 | 2.25E-04 | 148.2 |
| 1 | rs12088739 | 51,506,886 | *Y_RNA* | G/A | -0.088 | 0.013 | 9.79E-12 | 659,316 | 7.01E-05 | 46.2 |
| 8 | rs516946 | 41,519,248 | *RP11-930P14.1* | T/C | -0.082 | 0.009 | 3.16E-22 | 659,316 | 1.43E-04 | 94.0 |
| 13 | rs1359790 | 80,717,156 | *SPRY2* | A/G | -0.080 | 0.008 | 2.80E-23 | 659,316 | 1.50E-04 | 99.0 |
| 3 | rs1496653 | 23,454,790 | *UBE2E2* | G/A | -0.077 | 0.009 | 2.57E-18 | 659,316 | 1.16E-04 | 76.4 |
| 2 | rs13389219 | 165,528,876 | *COBLL1* | T/C | -0.072 | 0.007 | 2.11E-22 | 659,316 | 1.44E-04 | 95.2 |
| 9 | rs2796441 | 84,308,948 | *RP11-154D17.1* | A/G | -0.072 | 0.007 | 1.96E-22 | 659,316 | 1.45E-04 | 95.9 |
| 4 | rs735949 | 185,716,232 | *ACSL1* | C/T | -0.071 | 0.011 | 1.95E-11 | 659,316 | 6.82E-05 | 45.0 |
| 7 | rs2191348 | 15,064,255 | *AC006045.3* | G/T | -0.065 | 0.007 | 3.44E-19 | 659,316 | 1.21E-04 | 79.8 |
| 15 | rs7177055 | 77,832,762 | *RP11-307C19.1* | G/A | -0.065 | 0.008 | 2.75E-16 | 659,316 | 1.02E-04 | 67.1 |
| 1 | rs340874 | 214,159,256 | *PROX1* | T/C | -0.063 | 0.007 | 8.41E-18 | 659,316 | 1.12E-04 | 73.5 |
| 2 | rs2867125 | 622,827 | *TMEM18* | T/C | -0.060 | 0.010 | 4.32E-10 | 659,316 | 5.94E-05 | 39.2 |
| 7 | rs13234269 | 130,429,186 | *KLF14* | A/T | -0.058 | 0.008 | 6.98E-14 | 659,316 | 8.47E-05 | 55.9 |
| 16 | rs9940149 | 300,641 | *ITFG3* | A/G | -0.058 | 0.010 | 9.29E-10 | 659,316 | 5.65E-05 | 37.3 |
| 3 | rs6808574 | 187,740,523 | *RP11-132N15.2* | T/C | -0.055 | 0.008 | 4.38E-13 | 659,316 | 8.00E-05 | 52.8 |
| 2 | rs1009358 | 65,276,452 | *AC007386.4* | C/T | -0.055 | 0.008 | 9.81E-12 | 659,316 | 7.04E-05 | 46.4 |
| 3 | rs11925227 | 170,766,618 | *SLC2A2* | A/G | -0.053 | 0.010 | 2.25E-08 | 659,316 | 4.79E-05 | 31.6 |
| 5 | rs4865796 | 53,272,664 | *ARL15* | G/A | -0.053 | 0.008 | 1.33E-11 | 659,316 | 7.00E-05 | 46.2 |
| 10 | rs753270 | 80,964,975 | *ZMIZ1* | T/C | -0.053 | 0.008 | 2.70E-11 | 659,316 | 6.77E-05 | 44.7 |
| 12 | rs825476 | 124,568,456 | *RP11-522N14.1* | C/T | -0.052 | 0.007 | 6.80E-13 | 659,316 | 7.81E-05 | 51.5 |
| 1 | rs2820426 | 219,660,535 | *RP11-95P13.1* | A/G | -0.052 | 0.007 | 1.30E-12 | 659,316 | 7.73E-05 | 50.9 |
| 4 | rs7685296 | 153,254,121 | *FBXW7* | T/C | -0.051 | 0.008 | 2.32E-10 | 659,316 | 6.04E-05 | 39.8 |
| 9 | rs1758632 | 34,025,640 | *UBAP2* | C/G | -0.049 | 0.008 | 1.36E-09 | 659,316 | 5.57E-05 | 36.7 |
| 13 | rs963740 | 51,096,095 | *RP11-175B12.2* | T/A | -0.048 | 0.009 | 2.23E-08 | 659,316 | 4.71E-05 | 31.0 |
| 10 | rs11591741 | 101,976,501 | *CHUK* | C/G | -0.048 | 0.008 | 1.23E-09 | 659,316 | 5.62E-05 | 37.1 |
| 3 | rs3887925 | 186,665,645 | *ST6GAL1* | C/T | -0.047 | 0.008 | 2.47E-09 | 659,316 | 5.46E-05 | 36.0 |
| 4 | rs17086692 | 53,134,293 | *RP11-588F10.1* | T/G | -0.047 | 0.008 | 2.48E-08 | 659,316 | 4.69E-05 | 30.9 |
| 16 | rs244415 | 69,666,683 | *NFAT5* | A/G | -0.047 | 0.008 | 3.88E-09 | 659,316 | 5.30E-05 | 34.9 |
| 4 | rs7674212 | 103,988,899 | *SLC9B2* | T/G | -0.047 | 0.008 | 6.18E-10 | 659,316 | 5.83E-05 | 38.4 |
| 10 | rs2421016 | 124,167,512 | *PLEKHA1* | T/C | -0.046 | 0.007 | 1.48E-10 | 659,316 | 6.31E-05 | 41.6 |
| 8 | rs7845219 | 95,937,502 | *C8orf38* | C/T | -0.042 | 0.007 | 4.54E-09 | 659,316 | 5.21E-05 | 34.4 |
| 8 | rs7841082 | 8,168,987 | *PRAGMIN* | T/C | -0.042 | 0.008 | 4.94E-08 | 659,316 | 4.51E-05 | 29.8 |
| 15 | rs4502156 | 62,383,155 | *RP11-643M14.3* | C/T | -0.041 | 0.007 | 1.66E-08 | 659,316 | 4.81E-05 | 31.7 |
| 9 | rs10114341 | 96,919,182 | *MIRLET7A1* | C/T | -0.041 | 0.007 | 1.15E-08 | 659,316 | 4.89E-05 | 32.3 |
| 2 | rs7561798 | 228,973,660 | *SPHKAP* | G/A | 0.040 | 0.007 | 2.79E-08 | 659,316 | 4.68E-05 | 30.9 |
| 12 | rs1480474 | 66,326,943 | *HMGA2* | G/A | 0.041 | 0.007 | 1.68E-08 | 659,316 | 4.81E-05 | 31.7 |
| 7 | rs2299383 | 103,418,846 | *RELN* | T/C | 0.041 | 0.007 | 1.49E-08 | 659,316 | 4.83E-05 | 31.9 |
| 5 | rs1061813 | 14,847,331 | *ANKH* | G/A | 0.043 | 0.007 | 3.37E-09 | 659,316 | 5.24E-05 | 34.5 |
| 9 | rs687621 | 136,137,065 | *ABO* | G/A | 0.043 | 0.008 | 1.35E-08 | 659,316 | 4.92E-05 | 32.5 |
| 18 | rs7240767 | 7,070,642 | *LAMA1* | C/T | 0.045 | 0.008 | 2.16E-08 | 659,316 | 4.70E-05 | 31.0 |
| 3 | rs4472028 | 152,053,250 | *MBNL1* | T/C | 0.045 | 0.007 | 2.08E-10 | 659,316 | 6.17E-05 | 40.7 |
| 15 | rs982077 | 63,823,301 | *USP3* | A/G | 0.045 | 0.007 | 2.58E-10 | 659,316 | 6.00E-05 | 39.6 |
| 11 | rs7931302 | 128,236,058 | *ETS1* | C/A | 0.046 | 0.008 | 7.65E-09 | 659,316 | 5.04E-05 | 33.2 |
| 12 | rs11107116 | 93,978,504 | *SOCS2* | T/G | 0.047 | 0.009 | 3.75E-08 | 659,316 | 4.58E-05 | 30.2 |
| 14 | rs7144011 | 79,940,383 | *NRXN3* | T/G | 0.048 | 0.009 | 1.64E-08 | 659,316 | 4.88E-05 | 32.2 |
| 1 | rs348330 | 229,672,955 | *ABCB10* | G/A | 0.049 | 0.008 | 1.86E-09 | 659,316 | 5.48E-05 | 36.1 |
| 4 | rs993380 | 83,584,496 | *SCD5* | A/G | 0.051 | 0.008 | 4.59E-10 | 659,316 | 5.94E-05 | 39.2 |
| 15 | rs12910825 | 91,511,260 | *AC068831.8* | G/A | 0.052 | 0.007 | 2.16E-12 | 659,316 | 7.40E-05 | 48.8 |
| 16 | rs2925979 | 81,534,790 | *CMIP* | T/C | 0.053 | 0.008 | 9.06E-12 | 659,316 | 7.11E-05 | 46.9 |
| 18 | rs12970134 | 57,884,750 | *U4* | A/G | 0.056 | 0.008 | 5.31E-12 | 659,316 | 7.30E-05 | 48.1 |
| 5 | rs6878122 | 76,427,311 | *CTC-564N23.3* | G/A | 0.056 | 0.008 | 1.19E-12 | 659,316 | 7.73E-05 | 51.0 |
| 15 | rs4932143 | 90,372,067 | *ANPEP* | G/C | 0.057 | 0.009 | 5.51E-11 | 659,316 | 6.46E-05 | 42.6 |
| 11 | rs67232546 | 128,398,938 | *RP11-1007G5.2* | T/C | 0.060 | 0.010 | 4.66E-10 | 659,316 | 5.85E-05 | 38.5 |
| 8 | rs12681990 | 36,859,186 | *AC090453.1* | C/T | 0.063 | 0.010 | 3.62E-11 | 659,316 | 6.61E-05 | 43.6 |
| 6 | rs72892910 | 50,816,887 | *RPS17P5* | T/G | 0.065 | 0.010 | 6.43E-11 | 659,316 | 6.50E-05 | 42.8 |
| 20 | rs4810426 | 43,001,721 | *HNF4A* | T/C | 0.073 | 0.013 | 2.15E-08 | 659,316 | 4.73E-05 | 31.2 |
| 17 | rs11651755 | 36,099,840 | *HNF1B* | C/T | 0.074 | 0.008 | 8.98E-22 | 659,316 | 1.40E-04 | 92.6 |
| 7 | rs17168486 | 14,898,282 | *DGKB* | T/C | 0.074 | 0.009 | 2.18E-15 | 659,316 | 9.45E-05 | 62.3 |
| 11 | rs7929543 | 49,351,026 | *CTD-2026G22.1* | C/A | 0.083 | 0.014 | 2.20E-09 | 659,316 | 5.46E-05 | 36.0 |
| 11 | rs10830963 | 92,708,710 | *MTNR1B* | G/C | 0.091 | 0.008 | 5.85E-30 | 659,316 | 1.96E-04 | 129.1 |
| 12 | rs2261181 | 66,212,318 | *RPSAP52* | T/C | 0.099 | 0.012 | 9.18E-17 | 659,316 | 1.06E-04 | 69.7 |
| 7 | rs849135 | 28,196,413 | *JAZF1* | G/A | 0.100 | 0.007 | 1.04E-43 | 659,316 | 2.92E-04 | 192.5 |
| 16 | rs7185735 | 53,822,651 | *FTO* | G/A | 0.106 | 0.007 | 1.59E-47 | 659,316 | 3.17E-04 | 209.3 |
| 3 | rs7651090 | 185,513,392 | *IGF2BP2* | G/A | 0.120 | 0.008 | 3.85E-57 | 659,316 | 3.81E-04 | 251.0 |
| 5 | rs7729395 | 102,100,576 | *PAM* | T/C | 0.137 | 0.016 | 1.10E-17 | 659,316 | 1.12E-04 | 73.6 |

Chr: chromosome; SNP: the label of single-nucleotide polymorphism; Allele: effect allele and alternative allele; BETA: SNP effect size, SE: standard error of the SNP effect size; PVE: proportion of variance explained by the SNP; *P*, *N*, and *F* represent p value, sample size, and *F* statistic, respectively. All the selected instruments together explain about 0.61% phenotypic variation of type 2 diabetes at the observed scale. For these instrumental variables, all the *F* statistics are above ten (ranging from 29.8 to 251.0) with an average *F* statistic of 59.8 and an overall *F* statistic of 60.2.

Table S3 Summary information for instrumental variables of body mass index in the European population

| Chr | SNP | Position | Gene | ALLELE | BETA | SE | *P* | *N* | *PVE* | *F* |
| --- | --- | --- | --- | --- | --- | --- | --- | --- | --- | --- |
| 18 | rs1808579 | 21,104,888 | *C18orf8* | C/T | 0.0170 | 0.0030 | 4.17E-08 | 322,032 | 9.97E-05 | 32.1 |
| 9 | rs10733682 | 129,460,914 | *LMX1B* | A/G | 0.0170 | 0.0030 | 1.83E-08 | 320,727 | 1.00E-04 | 32.1 |
| 2 | rs11688816 | 63,053,048 | *EHBP1* | G/A | 0.0170 | 0.0030 | 1.89E-08 | 322,051 | 9.97E-05 | 32.1 |
| 9 | rs6477694 | 111,932,342 | *FRRS1L* | C/T | 0.0170 | 0.0030 | 2.67E-08 | 322,048 | 9.97E-05 | 32.1 |
| 16 | rs2080454 | 49,062,590 | *RP11-98C8.1* | C/A | 0.0170 | 0.0030 | 8.60E-09 | 322,099 | 9.97E-05 | 32.1 |
| 21 | rs2836754 | 40,291,740 | *AF064858.6* | C/T | 0.0170 | 0.0030 | 1.61E-08 | 320,231 | 1.00E-04 | 32.1 |
| 1 | rs977747 | 47,684,677 | *TAL1* | T/G | 0.0170 | 0.0030 | 2.18E-08 | 322,086 | 9.97E-05 | 32.1 |
| 13 | rs1441264 | 79,580,919 | *NIPA2P5* | A/G | 0.0170 | 0.0030 | 2.96E-08 | 310,286 | 1.03E-04 | 32.1 |
| 5 | rs7715256 | 153,537,893 | *MFAP3* | G/T | 0.0170 | 0.0030 | 8.85E-09 | 322,084 | 9.97E-05 | 32.1 |
| 17 | rs12940622 | 78,615,571 | *RPTOR* | G/A | 0.0180 | 0.0030 | 2.49E-09 | 322,032 | 1.12E-04 | 36.0 |
| 19 | rs29941 | 34,309,532 | *KCTD15* | G/A | 0.0180 | 0.0030 | 2.41E-08 | 321,970 | 1.12E-04 | 36.0 |
| 9 | rs4740619 | 15,634,326 | *C9orf93* | T/C | 0.0180 | 0.0030 | 4.56E-09 | 321,887 | 1.12E-04 | 36.0 |
| 15 | rs3736485 | 51,748,610 | *RP11-707P17.1* | A/G | 0.0180 | 0.0030 | 7.41E-09 | 321,398 | 1.12E-04 | 36.0 |
| 2 | rs1528435 | 181,550,962 | *AC009478.1* | T/C | 0.0180 | 0.0030 | 1.20E-08 | 321,924 | 1.12E-04 | 36.0 |
| 1 | rs11583200 | 50,559,820 | *ELAVL4* | C/T | 0.0180 | 0.0030 | 1.48E-08 | 322,095 | 1.12E-04 | 36.0 |
| 9 | rs1928295 | 120,378,483 | *RP11-500B12.1* | T/C | 0.0190 | 0.0030 | 7.91E-10 | 321,979 | 1.25E-04 | 40.1 |
| 16 | rs9925964 | 31,129,895 | *RP11-196G11.4* | A/G | 0.0190 | 0.0030 | 8.11E-10 | 318,385 | 1.26E-04 | 40.1 |
| 3 | rs6804842 | 25,106,437 | *AC133680.1* | G/A | 0.0190 | 0.0030 | 2.48E-09 | 321,463 | 1.25E-04 | 40.1 |
| 17 | rs1000940 | 5,283,252 | *NUP88* | G/A | 0.0190 | 0.0030 | 1.28E-08 | 321,836 | 1.25E-04 | 40.1 |
| 6 | rs9400239 | 108,977,663 | *FOXO3* | C/T | 0.0190 | 0.0030 | 1.61E-08 | 321,988 | 1.25E-04 | 40.1 |
| 3 | rs3849570 | 81,792,112 | *GBE1* | A/C | 0.0190 | 0.0030 | 2.60E-08 | 284,339 | 1.41E-04 | 40.1 |
| 8 | rs2033732 | 85,079,709 | *RP11-120I21.3* | C/T | 0.0190 | 0.0040 | 4.89E-08 | 321,406 | 7.02E-05 | 22.6 |
| 15 | rs7164727 | 73,093,991 | *RP11-361M10.5* | T/C | 0.0190 | 0.0030 | 3.92E-09 | 321,312 | 1.25E-04 | 40.1 |
| 3 | rs2365389 | 61,236,462 | *FHIT* | C/T | 0.0200 | 0.0030 | 1.63E-10 | 316,768 | 1.40E-04 | 44.4 |
| 1 | rs2820292 | 201,784,287 | *IPO9-AS1* | C/A | 0.0200 | 0.0030 | 1.83E-10 | 321,707 | 1.38E-04 | 44.4 |
| 7 | rs1167827 | 75,163,169 | *HIP1* | G/A | 0.0200 | 0.0030 | 6.33E-10 | 306,238 | 1.45E-04 | 44.4 |
| 11 | rs2176598 | 43,864,278 | *RP11-613D13.5* | T/C | 0.0200 | 0.0040 | 2.97E-08 | 316,848 | 7.89E-05 | 25.0 |
| 17 | rs9914578 | 2,005,136 | *SMG6* | G/C | 0.0200 | 0.0040 | 2.07E-08 | 321,126 | 7.78E-05 | 25.0 |
| 11 | rs4256980 | 8,673,939 | *TRIM66* | G/C | 0.0210 | 0.0030 | 2.90E-11 | 320,028 | 1.53E-04 | 49.0 |
| 1 | rs12401738 | 78,446,761 | *DNAJB4* | A/G | 0.0210 | 0.0030 | 1.15E-10 | 322,070 | 1.52E-04 | 49.0 |
| 14 | rs12885454 | 29,736,838 | *RP11-562L8.1* | C/A | 0.0210 | 0.0030 | 1.94E-10 | 320,823 | 1.53E-04 | 49.0 |
| 2 | rs11126666 | 26,928,811 | *KCNK3* | A/G | 0.0210 | 0.0030 | 1.33E-09 | 321,979 | 1.52E-04 | 49.0 |
| 16 | rs2650492 | 28,333,411 | *SBK1* | A/G | 0.0210 | 0.0040 | 1.92E-09 | 319,464 | 8.63E-05 | 27.6 |
| 2 | rs17203016 | 208,255,518 | *AC007879.5* | G/A | 0.0210 | 0.0040 | 3.41E-08 | 316,466 | 8.71E-05 | 27.6 |
| 13 | rs9540493 | 66,205,704 | *HNRNPA3P5* | A/G | 0.0210 | 0.0040 | 4.97E-08 | 318,961 | 8.64E-05 | 27.6 |
| 2 | rs1460676 | 164,567,689 | *FIGN* | C/T | 0.0210 | 0.0040 | 4.98E-08 | 322,089 | 8.56E-05 | 27.6 |
| 1 | rs11165643 | 96,924,097 | *EEF1A1P11* | T/C | 0.0220 | 0.0030 | 2.07E-12 | 320,730 | 1.68E-04 | 53.8 |
| 8 | rs17405819 | 76,806,584 | *snoU13* | T/C | 0.0220 | 0.0030 | 2.07E-11 | 322,085 | 1.67E-04 | 53.8 |
| 6 | rs205262 | 34,563,164 | *C6orf106* | G/A | 0.0220 | 0.0040 | 1.75E-10 | 315,542 | 9.59E-05 | 30.2 |
| 11 | rs12286929 | 115,022,404 | *ACA59* | G/A | 0.0220 | 0.0030 | 1.31E-12 | 321,903 | 1.67E-04 | 53.8 |
| 2 | rs7599312 | 213,413,231 | *MIR548F2* | G/A | 0.0220 | 0.0030 | 1.17E-10 | 322,024 | 1.67E-04 | 53.8 |
| 18 | rs7243357 | 56,883,319 | *GRP* | T/G | 0.0220 | 0.0040 | 3.86E-08 | 322,107 | 9.39E-05 | 30.2 |
| 16 | rs4787491 | 30,015,337 | *INO80E* | G/A | 0.0220 | 0.0040 | 2.70E-08 | 267,491 | 1.13E-04 | 30.2 |
| 2 | rs1016287 | 59,305,625 | *AC007131.3* | T/C | 0.0230 | 0.0030 | 2.25E-11 | 321,969 | 1.83E-04 | 58.8 |
| 1 | rs657452 | 49,589,847 | *RP11-141A19.1* | A/G | 0.0230 | 0.0030 | 5.48E-13 | 313,651 | 1.87E-04 | 58.8 |
| 10 | rs7903146 | 114,758,349 | *TCF7L2* | C/T | 0.0230 | 0.0030 | 1.11E-11 | 322,130 | 1.82E-04 | 58.8 |
| 14 | rs10132280 | 25,928,179 | *RP11-388G3.1* | C/A | 0.0230 | 0.0030 | 1.14E-11 | 321,797 | 1.83E-04 | 58.8 |
| 16 | rs758747 | 3,627,358 | *NLRC3* | T/C | 0.0230 | 0.0040 | 7.47E-10 | 308,688 | 1.07E-04 | 33.1 |
| 18 | rs7239883 | 40,147,671 | *RN5S454* | G/A | 0.0230 | 0.0040 | 1.51E-08 | 321,909 | 1.03E-04 | 33.1 |
| 6 | rs9374842 | 120,185,665 | *AL606830.1* | T/C | 0.0230 | 0.0040 | 2.67E-08 | 322,008 | 1.03E-04 | 33.1 |
| 1 | rs12566985 | 75,002,193 | *FPGT-TNNI3K* | G/A | 0.0240 | 0.0030 | 3.28E-15 | 319,282 | 2.00E-04 | 64.0 |
| 14 | rs7141420 | 79,899,454 | *NRXN3* | T/C | 0.0240 | 0.0030 | 1.23E-14 | 321,970 | 1.99E-04 | 64.0 |
| 2 | rs492400 | 219,349,752 | *USP37* | C/T | 0.0240 | 0.0040 | 6.78E-09 | 321,090 | 1.12E-04 | 36.0 |
| 6 | rs13201877 | 137,675,541 | *OLIG3* | G/A | 0.0240 | 0.0040 | 4.29E-08 | 322,095 | 1.12E-04 | 36.0 |
| 2 | rs2176040 | 227,092,802 | *AC068138.1* | A/G | 0.0240 | 0.0040 | 9.99E-09 | 321,972 | 1.12E-04 | 36.0 |
| 2 | rs2121279 | 143,043,285 | *AC016706.1* | T/C | 0.0250 | 0.0040 | 2.31E-08 | 322,065 | 1.21E-04 | 39.1 |
| 10 | rs17094222 | 102,395,440 | *HIF1AN* | C/T | 0.0250 | 0.0040 | 5.94E-11 | 321,770 | 1.21E-04 | 39.1 |
| 7 | rs6465468 | 95,169,514 | *ASB4* | T/G | 0.0250 | 0.0050 | 4.98E-08 | 307,937 | 8.12E-05 | 25.0 |
| 11 | rs3817334 | 47,650,993 | *MTCH2* | T/C | 0.0260 | 0.0030 | 5.15E-17 | 321,959 | 2.33E-04 | 75.1 |
| 5 | rs2112347 | 75,015,242 | *U6* | T/G | 0.0260 | 0.0030 | 6.19E-17 | 322,019 | 2.33E-04 | 75.1 |
| 19 | rs3810291 | 47,569,003 | *ZC3H4* | A/G | 0.0280 | 0.0040 | 4.81E-15 | 296,261 | 1.65E-04 | 49.0 |
| 6 | rs13191362 | 163,033,350 | *PARK2* | A/G | 0.0280 | 0.0050 | 7.34E-09 | 321,902 | 9.74E-05 | 31.4 |
| 7 | rs9641123 | 93,197,732 | *CALCR* | C/G | 0.0290 | 0.0050 | 2.08E-10 | 233,515 | 1.44E-04 | 33.6 |
| 3 | rs13078960 | 85,807,590 | *CADM2* | G/T | 0.0300 | 0.0040 | 1.74E-14 | 322,135 | 1.75E-04 | 56.2 |
| 20 | rs6091540 | 51,087,862 | *RP4-723E3.1* | C/T | 0.0300 | 0.0040 | 2.15E-11 | 321,975 | 1.75E-04 | 56.2 |
| 2 | rs10182181 | 25,150,296 | *DNAJC27* | G/A | 0.0310 | 0.0030 | 8.78E-24 | 321,759 | 3.32E-04 | 106.8 |
| 16 | rs3888190 | 28,889,486 | *RP11-22P6.3* | A/C | 0.0310 | 0.0030 | 3.14E-23 | 321,930 | 3.32E-04 | 106.8 |
| 15 | rs16951275 | 68,077,168 | *MAP2K5* | T/C | 0.0310 | 0.0040 | 1.91E-17 | 322,098 | 1.86E-04 | 60.1 |
| 4 | rs17001654 | 77,129,568 | *SCARB2* | G/C | 0.0310 | 0.0050 | 7.76E-09 | 233,722 | 1.64E-04 | 38.4 |
| 10 | rs11191560 | 104,869,038 | *NT5C2* | C/T | 0.0310 | 0.0050 | 8.45E-09 | 321,893 | 1.19E-04 | 38.4 |
| 12 | rs11057405 | 122,781,897 | *CLIP1* | G/A | 0.0310 | 0.0060 | 2.02E-08 | 314,111 | 8.50E-05 | 26.7 |
| 12 | rs7138803 | 50,247,468 | *RP11-70F11.2* | A/G | 0.0320 | 0.0030 | 8.15E-24 | 322,092 | 3.53E-04 | 113.8 |
| 7 | rs2245368 | 76,608,143 | *AC114737.4* | C/T | 0.0320 | 0.0060 | 3.19E-08 | 205,675 | 1.38E-04 | 28.4 |
| 1 | rs3101336 | 72,751,185 | *GDI2P2* | C/T | 0.0330 | 0.0030 | 2.66E-26 | 316,872 | 3.82E-04 | 121.0 |
| 13 | rs12429545 | 54,102,206 | *AL450423.1* | A/G | 0.0330 | 0.0050 | 1.09E-12 | 312,934 | 1.39E-04 | 43.6 |
| 4 | rs11727676 | 145,659,064 | *HHIP* | T/C | 0.0360 | 0.0060 | 2.55E-08 | 296,401 | 1.21E-04 | 36.0 |
| 4 | rs10938397 | 45,182,527 | *PRKRIRP9* | G/A | 0.0400 | 0.0030 | 3.21E-38 | 320,955 | 5.54E-04 | 177.8 |
| 16 | rs12446632 | 19,935,389 | *GPRC5B* | G/A | 0.0400 | 0.0050 | 1.48E-18 | 316,758 | 2.02E-04 | 64.0 |
| 10 | rs7899106 | 87,410,904 | *GRID1* | G/A | 0.0400 | 0.0070 | 2.96E-08 | 321,770 | 1.01E-04 | 32.7 |
| 11 | rs11030104 | 27,684,517 | *BDNF* | A/G | 0.0410 | 0.0040 | 5.56E-28 | 322,103 | 3.26E-04 | 105.1 |
| 6 | rs2207139 | 50,845,490 | *RP4-753D5.3* | G/A | 0.0450 | 0.0040 | 4.13E-29 | 322,019 | 3.93E-04 | 126.6 |
| 3 | rs1516725 | 185,824,004 | *ETV5* | C/T | 0.0450 | 0.0050 | 1.89E-22 | 320,644 | 2.53E-04 | 81.0 |
| 8 | rs16907751 | 81,375,457 | *Y_RNA* | C/T | 0.0470 | 0.0090 | 3.89E-08 | 307,752 | 8.86E-05 | 27.3 |
| 1 | rs543874 | 177,889,480 | *RP4-798P15.2* | G/A | 0.0480 | 0.0040 | 2.62E-35 | 322,008 | 4.47E-04 | 144.0 |
| 4 | rs13107325 | 103,188,709 | *SLC39A8* | T/C | 0.0480 | 0.0070 | 1.83E-12 | 321,461 | 1.46E-04 | 47.0 |
| 3 | rs16851483 | 141,275,436 | *RASA2* | T/G | 0.0480 | 0.0080 | 3.55E-10 | 233,929 | 1.54E-04 | 36.0 |
| 14 | rs11847697 | 30,515,112 | *CTD-2251F13.1* | T/C | 0.0490 | 0.0080 | 3.99E-09 | 306,243 | 1.22E-04 | 37.5 |
| 18 | rs6567160 | 57,829,135 | *U4* | C/T | 0.0560 | 0.0040 | 3.93E-53 | 321,958 | 6.08E-04 | 196.0 |
| 2 | rs13021737 | 632,348 | *TMEM18* | G/A | 0.0600 | 0.0040 | 1.11E-50 | 318,287 | 7.06E-04 | 225.0 |
| 1 | rs17024393 | 110,154,688 | *GNAT2* | C/T | 0.0660 | 0.0090 | 7.03E-14 | 297,874 | 1.81E-04 | 53.8 |
| 16 | rs1558902 | 53,803,574 | *FTO* | A/T | 0.0820 | 0.0030 | 0.00E+00 | 320,073 | 2.33E-03 | 747.1 |

Chr: chromosome; SNP: the label of single-nucleotide polymorphism; Allele: effect allele and alternative allele; BETA: SNP effect size, SE: standard error of the SNP effect size; PVE: proportion of variance explained by the SNP; *P*, *N*, and *F* represent p value, sample size, and *F* statistic, respectively. All the selected instruments together explain about 1.73% phenotypic variation of body mass index. For these instrumental variables, all the *F* statistics are above ten (ranging from 22.6 to 747.1) with an average *F* statistic of 60.2 and an overall *F* statistic of 60.7.

Table S4 Summary information for instrumental variables of fasting glucose in the European population

| Chr | SNP | Position | Gene | ALLELE | BETA | SE | *P* | *N* | *PVE* | *F* |
| --- | --- | --- | --- | --- | --- | --- | --- | --- | --- | --- |
| 1 | rs340874 | 214,159,256 | *PROX1* | C/T | 0.0139 | 2.00E-03 | 4.08E-10 | 133,010 | 3.63E-04 | 48.3 |
| 2 | rs1371614 | 27,152,874 | *DPYSL5* | T/C | 0.0188 | 3.00E-03 | 3.15E-11 | 133,010 | 2.96E-04 | 39.4 |
| 2 | rs780094 | 27,741,237 | *GCKR* | C/T | 0.0276 | 2.00E-03 | 2.58E-37 | 133,010 | 1.43E-03 | 190.6 |
| 2 | rs3736594 | 27,995,781 | *MRPL33* | A/C | 0.0218 | 3.00E-03 | 1.56E-16 | 133,010 | 3.95E-04 | 52.6 |
| 2 | rs560887 | 169,763,148 | *G6PC2* | C/T | 0.0714 | 3.00E-03 | 0.00E+00 | 133,010 | 4.24E-03 | 566.3 |
| 3 | rs11715915 | 49,455,330 | *AMT* | C/T | 0.0119 | 2.00E-03 | 4.90E-08 | 133,010 | 2.67E-04 | 35.6 |
| 3 | rs11708067 | 123,065,778 | *ADCY5* | A/G | 0.0227 | 3.00E-03 | 1.30E-18 | 133,010 | 4.32E-04 | 57.5 |
| 3 | rs1280 | 170,713,290 | *U1* | T/C | 0.0266 | 3.00E-03 | 8.56E-18 | 133,010 | 5.93E-04 | 78.9 |
| 3 | rs7651090 | 185,513,392 | *IGF2BP2* | G/A | 0.0129 | 2.00E-03 | 1.75E-08 | 133,010 | 3.13E-04 | 41.7 |
| 5 | rs4869272 | 95,539,448 | *RP11-254I22.1* | T/C | 0.0178 | 2.00E-03 | 1.02E-15 | 133,010 | 5.98E-04 | 79.6 |
| 6 | rs9368222 | 20,686,996 | *CDKAL1* | A/C | 0.0139 | 2.00E-03 | 1.00E-09 | 133,010 | 3.63E-04 | 48.3 |
| 7 | rs2191349 | 15,064,309 | *AC006045.3* | T/G | 0.0296 | 2.00E-03 | 1.28E-42 | 133,010 | 1.64E-03 | 218.4 |
| 7 | rs730497 | 44,223,721 | *GCK* | A/G | 0.0573 | 3.00E-03 | 3.70E-87 | 133,010 | 2.74E-03 | 365.1 |
| 7 | rs6943153 | 50,791,579 | *GRB10* | T/C | 0.0159 | 2.00E-03 | 1.63E-12 | 133,010 | 4.73E-04 | 63.0 |
| 8 | rs983309 | 9,177,732 | *RP11-115J16.1* | T/G | 0.0257 | 3.00E-03 | 6.29E-15 | 133,010 | 5.50E-04 | 73.2 |
| 8 | rs11558471 | 118,185,733 | *SLC30A8* | A/G | 0.0286 | 2.00E-03 | 7.80E-37 | 133,010 | 1.53E-03 | 204.3 |
| 9 | rs10814916 | 4,293,150 | *GLIS3* | C/A | 0.0159 | 2.00E-03 | 2.26E-13 | 133,010 | 4.73E-04 | 63.0 |
| 9 | rs10811661 | 22,134,094 | *RP11-408N14.1* | T/C | 0.0237 | 3.00E-03 | 5.65E-18 | 133,010 | 4.70E-04 | 62.5 |
| 9 | rs16913693 | 111,680,359 | *IKBKAP* | T/G | 0.0431 | 7.00E-03 | 3.51E-11 | 133,010 | 2.84E-04 | 37.8 |
| 9 | rs3829109 | 139,256,766 | *DNLZ* | G/A | 0.0169 | 3.00E-03 | 1.13E-10 | 133,010 | 2.37E-04 | 31.6 |
| 10 | rs11195502 | 113,039,667 | *RP11-381K7.2* | C/T | 0.0325 | 4.00E-03 | 1.97E-18 | 133,010 | 4.95E-04 | 65.9 |
| 10 | rs7903146 | 114,758,349 | *TCF7L2* | T/C | 0.0218 | 2.00E-03 | 2.71E-20 | 133,010 | 8.89E-04 | 118.4 |
| 11 | rs11605924 | 45,873,091 | *CRY2* | A/C | 0.0198 | 2.00E-03 | 3.93E-19 | 133,010 | 7.37E-04 | 98.0 |
| 11 | rs11039182 | 47,346,723 | *MADD* | T/C | 0.0237 | 2.00E-03 | 4.82E-22 | 133,010 | 1.06E-03 | 140.6 |
| 11 | rs1483121 | 48,333,360 | *OR4S1* | G/A | 0.0218 | 4.00E-03 | 1.57E-09 | 133,010 | 2.22E-04 | 29.6 |
| 11 | rs174576 | 61,603,510 | *FADS1* | C/A | 0.0198 | 2.00E-03 | 1.18E-18 | 133,010 | 7.37E-04 | 98.0 |
| 11 | rs11603334 | 72,432,985 | *ARAP1* | G/A | 0.0188 | 3.00E-03 | 1.12E-11 | 133,010 | 2.96E-04 | 39.4 |
| 11 | rs10830963 | 92,708,710 | *MTNR1B* | G/C | 0.0779 | 3.00E-03 | 0.00E+00 | 133,010 | 5.04E-03 | 674.0 |
| 12 | rs10747083 | 133,041,618 | *RP11-503G7.2* | A/G | 0.0129 | 2.00E-03 | 7.57E-09 | 133,010 | 3.13E-04 | 41.7 |
| 13 | rs11619319 | 28,487,599 | *PDX1-AS1* | G/A | 0.0198 | 2.00E-03 | 1.33E-15 | 133,010 | 7.37E-04 | 98.0 |
| 13 | rs576674 | 33,554,302 | *TOMM22P3* | G/A | 0.0169 | 3.00E-03 | 2.26E-08 | 133,010 | 2.37E-04 | 31.6 |
| 14 | rs3783347 | 100,839,261 | *WARS* | G/T | 0.0169 | 3.00E-03 | 1.32E-10 | 133,010 | 2.37E-04 | 31.6 |
| 15 | rs4502156 | 62,383,155 | *RP11-643M14.3* | T/C | 0.0227 | 2.00E-03 | 1.38E-25 | 133,010 | 9.71E-04 | 129.3 |
| 20 | rs6113722 | 22,557,099 | *LINC00261* | G/A | 0.0354 | 5.00E-03 | 2.49E-11 | 133,010 | 3.76E-04 | 50.0 |
| 20 | rs6072275 | 39,743,905 | *RP1-1J6.2* | A/G | 0.0159 | 3.00E-03 | 1.66E-08 | 133,010 | 2.10E-04 | 28.0 |

Chr: chromosome; SNP: the label of single-nucleotide polymorphism; Allele: effect allele and alternative allele; BETA: SNP effect size, SE: standard error of the SNP effect size; PVE: proportion of variance explained by the SNP; *P*, *N*, and *F* represent p value, sample size, and *F* statistic, respectively. All the selected instruments together explain about 3.02% phenotypic variation of fasting glucose. For these instrumental variables, all the *F* statistics are above ten (ranging from 28.0 to 674.0) with an average *F* statistic of 115.2 and an overall *F* statistic of 118.3.

Table S5 Summary information for instrumental variables of fasting insulin in the European population

| Chr | SNP | Position | Gene | ALLELE | BETA | SE | *P* | *N* | *PVE* | *F* |
| --- | --- | --- | --- | --- | --- | --- | --- | --- | --- | --- |
| 1 | rs2820436 | 219,640,680 | *RP11-95P13.1* | C/A | 0.0149 | 0.0030 | 4.36E-09 | 108,557 | 2.27E-04 | 24.6 |
| 2 | rs780094 | 27,741,237 | *GCKR* | C/T | 0.0188 | 0.0030 | 7.06E-14 | 108,557 | 3.62E-04 | 39.4 |
| 2 | rs1530559 | 135,755,629 | *YSK4* | A/G | 0.0149 | 0.0030 | 3.37E-08 | 108,557 | 2.27E-04 | 24.6 |
| 2 | rs10195252 | 165,513,091 | *COBLL1* | T/C | 0.0159 | 0.0030 | 4.87E-10 | 108,557 | 2.58E-04 | 28.0 |
| 2 | rs2972143 | 227,116,365 | *AC068138.1* | G/A | 0.0139 | 0.0030 | 3.15E-08 | 108,557 | 1.98E-04 | 21.5 |
| 4 | rs9884482 | 106,081,636 | *TET2* | C/T | 0.0169 | 0.0020 | 1.40E-11 | 108,557 | 6.54E-04 | 71.0 |
| 5 | rs4865796 | 53,272,664 | *ARL15* | A/G | 0.0149 | 0.0030 | 2.09E-08 | 108,557 | 2.27E-04 | 24.6 |
| 6 | rs2745353 | 127,452,935 | *RSPO3* | T/C | 0.0139 | 0.0030 | 5.48E-09 | 108,557 | 1.98E-04 | 21.5 |
| 7 | rs1167800 | 75,176,196 | *HIP1* | A/G | 0.0159 | 0.0030 | 2.61E-09 | 108,557 | 2.58E-04 | 28.0 |
| 8 | rs983309 | 9,177,732 | *RP11-115J16.1* | T/G | 0.0286 | 0.0040 | 3.81E-14 | 108,557 | 4.70E-04 | 51.1 |
| 10 | rs7903146 | 114,758,349 | *TCF7L2* | C/T | 0.0178 | 0.0030 | 6.13E-11 | 108,557 | 3.26E-04 | 35.4 |
| 12 | rs860598 | 102,898,446 | *RP11-210L7.1* | A/G | 0.0178 | 0.0030 | 1.64E-08 | 108,557 | 3.26E-04 | 35.4 |
| 16 | rs1421085 | 53,800,954 | *FTO* | C/T | 0.0198 | 0.0030 | 1.87E-15 | 108,557 | 4.01E-04 | 43.6 |
| 19 | rs731839 | 33,899,065 | *PEPD* | G/A | 0.0149 | 0.0030 | 1.72E-08 | 108,557 | 2.27E-04 | 24.6 |

Chr: chromosome; SNP: the label of single-nucleotide polymorphism; Allele: effect allele and alternative allele; BETA: SNP effect size, SE: standard error of the SNP effect size; PVE: proportion of variance explained by the SNP; *P*, *N*, and *F* represent p value, sample size, and *F* statistic, respectively. All the selected instruments together explain about 0.44% phenotypic variation of fasting insulin. For these instrumental variables, all the *F* statistics are above ten (ranging from 21.5 to 71.0) with an average *F* statistic of 33.8 and an overall *F* statistic of 34.3.

Table S6 Summary information for instrumental variables of hemoglobin A1c in the European population

| Chr | SNP | Position | Gene | ALLELE | BETA | SE | *P* | *N* | *PVE* | *F* |
| --- | --- | --- | --- | --- | --- | --- | --- | --- | --- | --- |
| 1 | rs267738 | 150,940,625 | *CERS2* | G/T | -0.0111 | 0.0020 | 2.59E-09 | 123,665 | 2.47E-04 | 30.6 |
| 1 | rs2246434 | 158,618,455 | *SPTA1* | A/G | 0.0188 | 0.0020 | 1.99E-27 | 123,665 | 7.16E-04 | 88.6 |
| 2 | rs17509001 | 24,021,231 | *ATAD2B* | C/T | 0.0178 | 0.0020 | 1.94E-15 | 123,665 | 6.43E-04 | 79.6 |
| 2 | rs12621844 | 48,414,735 | *AC079807.4* | C/T | -0.0101 | 0.0020 | 1.87E-08 | 123,665 | 2.04E-04 | 25.3 |
| 2 | rs560887 | 169,763,148 | *G6PC2* | C/T | 0.0276 | 0.0020 | 1.48E-58 | 123,665 | 1.54E-03 | 190.6 |
| 3 | rs7616006 | 12,267,648 | *GSTM5P1* | A/G | 0.0100 | 0.0020 | 5.07E-10 | 123,665 | 2.00E-04 | 24.8 |
| 3 | rs9818758 | 49,382,925 | *GPX1* | A/G | 0.0119 | 0.0020 | 7.74E-10 | 123,665 | 2.88E-04 | 35.6 |
| 3 | rs11708067 | 123,065,778 | *ADCY5* | A/G | 0.0129 | 0.0020 | 1.42E-12 | 123,665 | 3.37E-04 | 41.7 |
| 3 | rs8192675 | 170,724,883 | *SLC2A2* | C/T | -0.0111 | 0.0020 | 1.38E-11 | 123,665 | 2.47E-04 | 30.6 |
| 4 | rs13134327 | 144,659,795 | *RP13-578N3.3* | A/G | 0.0129 | 0.0020 | 2.64E-15 | 123,665 | 3.37E-04 | 41.7 |
| 6 | rs7756992 | 20,679,709 | *CDKAL1* | A/G | -0.0121 | 0.0020 | 2.80E-12 | 123,665 | 2.95E-04 | 36.4 |
| 6 | rs1800562 | 26,093,141 | *HFE* | A/G | -0.0398 | 0.0040 | 4.67E-28 | 123,665 | 7.99E-04 | 98.9 |
| 6 | rs11964178 | 109,562,035 | *C6orf183* | A/G | 0.0100 | 0.0020 | 6.38E-10 | 123,665 | 2.00E-04 | 24.8 |
| 6 | rs1547247 | 135,390,836 | *HBS1L* | A/G | -0.0141 | 0.0020 | 1.73E-17 | 123,665 | 4.02E-04 | 49.7 |
| 6 | rs592423 | 139,840,693 | *RP11-12A2.3* | A/C | 0.0090 | 0.0020 | 3.96E-08 | 123,665 | 1.62E-04 | 20.1 |
| 7 | rs4607517 | 44,235,668 | *GCK* | A/G | 0.0305 | 0.0020 | 8.76E-38 | 123,665 | 1.88E-03 | 233.0 |
| 8 | rs4737009 | 41,630,405 | *ANK1* | A/G | 0.0208 | 0.0020 | 4.48E-27 | 123,665 | 8.72E-04 | 108.0 |
| 8 | rs6980507 | 42,383,084 | *SLC20A2* | A/G | 0.0100 | 0.0020 | 3.58E-08 | 123,665 | 2.00E-04 | 24.8 |
| 8 | rs13266634 | 118,184,783 | *SLC30A8* | C/T | 0.0149 | 0.0020 | 4.53E-20 | 123,665 | 4.48E-04 | 55.4 |
| 9 | rs2383208 | 22,132,076 | *CDKN2B-AS1* | A/G | 0.0139 | 0.0020 | 7.04E-12 | 123,665 | 3.91E-04 | 48.3 |
| 9 | rs7040409 | 91,503,236 | *PCNPP2* | C/G | 0.0276 | 0.0040 | 2.56E-14 | 123,665 | 3.85E-04 | 47.7 |
| 9 | rs579459 | 136,154,168 | *ABO* | C/T | 0.0109 | 0.0020 | 9.42E-09 | 123,665 | 2.42E-04 | 29.9 |
| 10 | rs4745982 | 71,089,843 | *HK1* | G/T | -0.0954 | 0.0060 | 2.87E-65 | 123,665 | 2.04E-03 | 252.9 |
| 10 | rs17747324 | 114,752,503 | *TCF7L2* | C/T | 0.0149 | 0.0020 | 6.12E-11 | 123,665 | 4.48E-04 | 55.4 |
| 11 | rs3782123 | 205,198 | *RP11-304M2.5* | A/C | -0.0131 | 0.0020 | 1.51E-10 | 123,665 | 3.46E-04 | 42.8 |
| 11 | rs11603334 | 72,432,985 | *ARAP1* | A/G | -0.0121 | 0.0020 | 6.85E-09 | 123,665 | 2.95E-04 | 36.4 |
| 11 | rs1387153 | 92,673,828 | *RP11-676F20.3* | C/T | -0.0192 | 0.0020 | 2.11E-24 | 123,665 | 7.43E-04 | 92.0 |
| 12 | rs10774625 | 111,910,219 | *ATXN2* | A/G | -0.0090 | 0.0020 | 1.46E-08 | 123,665 | 1.65E-04 | 20.4 |
| 13 | rs423117 | 113,346,107 | *ATP11A* | C/T | -0.0192 | 0.0030 | 1.30E-12 | 123,665 | 3.31E-04 | 40.9 |
| 13 | rs7994900 | 114,553,134 | *GAS6* | A/G | 0.0100 | 0.0020 | 4.88E-09 | 123,665 | 2.00E-04 | 24.8 |
| 16 | rs11248914 | 293,562 | *ITFG3* | C/T | -0.0141 | 0.0020 | 2.56E-14 | 123,665 | 4.02E-04 | 49.7 |
| 16 | rs9935401 | 53,816,838 | *FTO* | A/G | 0.0100 | 0.0020 | 1.87E-08 | 123,665 | 2.00E-04 | 24.8 |
| 16 | rs837763 | 88,853,729 | *CDT1* | C/T | -0.0171 | 0.0020 | 1.68E-28 | 123,665 | 5.94E-04 | 73.5 |
| 17 | rs9914988 | 27,183,104 | *ERAL1* | A/G | 0.0129 | 0.0020 | 2.77E-11 | 123,665 | 3.37E-04 | 41.7 |
| 17 | rs1046896 | 80,685,533 | *FN3KRP* | C/T | -0.0284 | 0.0020 | 4.46E-64 | 123,665 | 1.63E-03 | 201.6 |
| 22 | rs855791 | 37,462,936 | *TMPRSS6* | A/G | 0.0169 | 0.0020 | 3.44E-28 | 123,665 | 5.74E-04 | 71.0 |

Chr: chromosome; SNP: the label of single-nucleotide polymorphism; Allele: effect allele and alternative allele; BETA: SNP effect size, SE: standard error of the SNP effect size; PVE: proportion of variance explained by the SNP; *P*, *N*, and *F* represent p value, sample size, and *F* statistic, respectively. All the selected instruments together explain about 1.93% phenotypic variation of hemoglobin A1c. For these instrumental variables, all the *F* statistics are above ten (ranging from 20.1 to 252.9) with an average *F* statistic of 66.5 and an overall *F* statistic of 67.6.

Table S7 Summary information for T2D instrumental variables that were associated with other traits or diseases

| Instrumental  variable | Disease/trait | Nearest gene | Reference |
| --- | --- | --- | --- |
| rs10830963 | Corrected insulin response | *MTNR1B* |  |
| rs10830963 | Corrected insulin response adjusted for insulin sensitivity index | *MTNR1B* |  |
| rs10830963 | Incremental insulin | *MTNR1B* |  |
| rs10830963 | Insulin disposition index | *MTNR1B* |  |
| rs10830963 | Insulin levels | *MTNR1B* |  |
| rs10830963 | Insulin levels adjusted for BMI | *MTNR1B* |  |
| rs340874 | Fasting blood glucose | *PROX1* |  |
| rs340874 | Fasting blood glucose (BMI interaction) | *PROX1* |  |
| rs10830963 | Fasting blood glucose | *MTNR1B* |  |
| rs10830963 | Fasting blood glucose (BMI interaction) | *MTNR1B* |  |
| rs687621 | Venous thromboembolism | *ABO* |  |
| rs7651090 | Acute insulin response | *IGF2BP2* |  |
| rs10830963 | Acute insulin response | *MTNR1B* |  |
| rs10830963 | Insulin disposition index | *MTNR1B* |  |
| rs10830963 | Insulin secretion rate | *MTNR1B* |  |
| rs10830963 | Peak insulin response | *MTNR1B* |  |
| rs4865796 | Body mass index | *ARL15* |  |
| rs7144011 | Body mass index | *NRXN3* |  |
| rs2925979 | Adiponectin levels | *CMIP* |  |
| rs2867125 | Body mass index | *TMEM18* |  |
| rs2925979 | HDL cholesterol | *CMIP* |  |
| rs2925979 | HDL cholesterol | *CMIP* |  |
| rs12970134 | Waist circumference and related phenotypes | *MC4R* |  |
| rs2925979 | HDL cholesterol | *CMIP* |  |
| rs10830963 | Glycemic traits (multi-trait analysis) | *MTNR1B* |  |
| rs10830963 | Fasting plasma glucose | *MTNR1B* |  |
| rs10830963 | Pulse pressure | *MTNR1B* |  |
| rs17168486 | Blood sugar levels | *DGKB* |  |
| rs10830963 | Hemoglobin A1c levels | *MTNR1B* |  |
| rs2925979 | High density lipoprotein cholesterol levels | *CMIP* |  |
| rs687621 | Monocyte count | *ABO* |  |
| rs687621 | Activated partial thromboplastin time | *LOC653163, SURF2,*  *SURF4, ADAMTS13,*  *C9orf7, ABO* |  |
| rs687621 | D-dimer levels | *ABO* |  |
| rs10830963 | Glucose homeostasis traits | *FAT3, MTNR1B* |  |
| rs10830963 | Glucose homeostasis traits | *MTNR1B* |  |
| rs2925979 | HDL cholesterol | *CMIP* |  |
| rs2925979 | Triglycerides | *CMIP* |  |
| rs11107116 | Height | *SOCS2* |  |
| rs4502156 | Proinsulin levels | *C2CD4B, C2CD4A,*  *VPS13C* |  |
| rs10830963 | Metabolite levels | *MTNR1B* |  |
| rs10830963 | Offspring birth weight | *MTNR1B* |  |
| rs2796441 | Schizophrenia | *TLE1* |  |
| rs10830963 | Fasting blood glucose | *MTNR1B* |  |
| rs12970134 | Body mass index | *MC4R* |  |
| rs12970134 | Weight | *MC4R* |  |
| rs2421016 | Birth weight | *PLEKHA1* |  |
| rs10830963 | Birth weight | *MTNR1B* |  |
| rs13389219 | Waist-hip ratio | *COBLL1* |  |
| rs7185735 | Obesity | *FTO* |  |
| rs2925979 | Waist-to-hip ratio adjusted for BMI (adjusted for smoking behavior) | *CMIP* |  |
| rs2925979 | Waist-to-hip ratio adjusted for BMI (adjusted for smoking behavior) | *CMIP* |  |
| rs2867125 | Body mass index | *TMEM18* |  |
| rs2867125 | Body mass index | *TMEM18* |  |
| rs2867125 | Body mass index | *TMEM18* |  |
| rs2867125 | Body mass index | *TMEM18* |  |
| rs2867125 | Body mass index | *TMEM18* |  |
| rs2867125 | Body mass index (joint analysis main effects and physical activity interaction) | *TMEM18* |  |
| rs2867125 | Body mass index (joint analysis main effects and physical activity interaction) | *TMEM18* |  |
| rs2867125 | Body mass index (joint analysis main effects and physical activity interaction) | *TMEM18* |  |
| rs2867125 | Body mass index (joint analysis main effects and physical activity interaction) | *TMEM18* |  |
| rs2867125 | Body mass index (joint analysis main effects and physical activity interaction) | *TMEM18* |  |
| rs2867125 | Body mass index in physically active individuals | *TMEM18* |  |
| rs2867125 | Body mass index in physically active individuals | *TMEM18* |  |
| rs2867125 | Body mass index in physically active individuals | *TMEM18* |  |
| rs2867125 | Body mass index in physically active individuals | *TMEM18* |  |
| rs2867125 | Body mass index in physically active individuals | *TMEM18* |  |
| rs2867125 | Body mass index in physically active individuals | *TMEM18* |  |
| rs2867125 | Body mass index in physically inactive individuals | *TMEM18* |  |
| rs2867125 | Body mass index in physically inactive individuals | *TMEM18* |  |
| rs2867125 | Body mass index in physically inactive individuals | *TMEM18* |  |
| rs2867125 | Body mass index in physically inactive individuals | *TMEM18* |  |
| rs2925979 | Waist-to-hip ratio adjusted for BMI (joint analysis for main effect and physical activity interaction) | *CMIP* |  |
| rs2925979 | Waist-to-hip ratio adjusted for BMI (joint analysis for main effect and physical activity interaction) | *CMIP* |  |
| rs2925979 | Waist-to-hip ratio adjusted for body mass index | *CMIP* |  |
| rs687621 | Blood protein levels | *ABO* |  |
| rs687621 | Blood protein levels | *ABO* |  |
| rs11107116 | Height | *SOCS2* |  |
| rs11651755 | Epithelial ovarian cancer | *HNF1B* |  |
| rs11651755 | High-grade serous ovarian cancer | *HNF1B* |  |
| rs11651755 | Low-grade serous and serous borderline ovarian cancer | *HNF1B* |  |
| rs11651755 | Mucinous ovarian carcinoma | *HNF1B* |  |
| rs11651755 | Ovarian clear cell cancer | *HNF1B* |  |
| rs11651755 | Serous borderline ovarian cancer | *HNF1B* |  |
| rs11651755 | Serous invasive ovarian cancer | *HNF1B* |  |
| rs10830963 | Glycated hemoglobin levels | *MTNR1B* |  |
| rs10830963 | Glycated hemoglobin levels | *MTNR1B* |  |
| rs11107116 | Height | *SOCS2* |  |
| rs7185735 | Subcutaneous adipose tissue | *FTO* |  |
| rs340874 | Fasting blood glucose | *PROX1* |  |
| rs340874 | Homeostasis model assessment of beta-cell function | *PROX1* |  |
| rs10830963 | Fasting blood glucose | *MTNR1B* |  |
| rs10830963 | Homeostasis model assessment of beta-cell function | *MTNR1B* |  |
| rs7144011 | Hip circumference | *NRXN3* |  |
| rs7144011 | Hip circumference | *NRXN3* |  |
| rs7144011 | Waist circumference | *NRXN3* |  |
| rs7144011 | Waist circumference | *NRXN3* |  |
| rs7144011 | Waist circumference | *NRXN3* |  |
| rs2925979 | Waist-to-hip ratio adjusted for body mass index | *CMIP* |  |
| rs2925979 | Waist-to-hip ratio adjusted for body mass index | *CMIP* |  |
| rs2925979 | Waist-to-hip ratio adjusted for body mass index | *CMIP* |  |
| rs2925979 | Waist-to-hip ratio adjusted for body mass index | *CMIP* |  |
| rs687621 | WF levels | *ABO* |  |
| rs7185735 | Pulse pressure x alcohol consumption interaction | *FTO* |  |
| rs10830963 | Obesity-related traits | *MTNR1B, LOC642791* |  |
| rs2925979 | Adiponectin levels | *CMIP* |  |
| rs13389219 | High light scatter reticulocyte count | *COBLL1* |  |
| rs2867125 | Body mass index | *TMEM18, ACP1,*  *FAM150B, SH3YL1,*  *SNTG2, LOC339822* |  |
| rs2867125 | Body mass index (age <50) | *TMEM18* |  |
| rs2867125 | Body mass index (age>50) | *TMEM18, ACP1,*  *FAM150B, SH3YL1,*  *SNTG2, LOC339822* |  |
| rs2867125 | Body mass index x age interaction | *TMEM18, ACP1,*  *FAM150B, SH3YL1,*  *SNTG2, LOC339822* |  |
| rs2867125 | Body mass index x sex x age interaction | *TMEM18, ACP1,*  *FAM150B, SH3YL1,*  *SNTG2, LOC339822* |  |
| rs2925979 | Waist-to-hip ratio adjusted for BMI x sex interaction | *CMIP* |  |
| rs2925979 | Waist-to-hip ratio adjusted for BMI x sex x age interaction | *CMIP* |  |
| rs687621 | Diastolic blood pressure | *ABO* |  |
| rs10830963 | Fasting plasma glucose | *MTNR1B* |  |
| rs10077431 | Blood lipids | *MSR1* |  |
| rs10087241 | Blood lipids | *WFS1* |  |
| rs10100265 | Blood lipids | *CTLA4* |  |
| rs10169613 | Blood lipids | *RAD54L* |  |
| rs10401969 | Blood lipids | *IL12B* |  |
| rs1050226 | Blood lipids | *ERCC3* |  |
| rs1063192 | Blood lipids | *PEX10* |  |
| rs1063355 | Blood lipids | *KCNN2* |  |
| rs10811661 | Blood lipids | *MTMR14* |  |
| rs10974438 | Blood lipids | *POLE4* |  |
| rs11048456 | Blood lipids | *CD14* |  |
| rs11098676 | Blood lipids | *NOS1AP* |  |
| rs11257655 | Blood lipids | *PECR* |  |
| rs1127655 | Blood lipids | *TSHR* |  |
| rs11708067 | Blood lipids | *AKR1A1* |  |
| rs11774915 | Blood lipids | *CALCRL* |  |
| rs11926707 | Blood lipids | *NBL1* |  |
| rs12299509 | Blood lipids | *TNFRSF18* |  |
| rs12617659 | Blood lipids | *INS* |  |
| rs12945601 | Blood lipids | *SHFM1* |  |
| rs13239186 | Blood lipids | *CYP2E1* |  |
| rs1552224 | Blood lipids | *TRIP12* |  |
| rs16988333 | Blood lipids | *ARHGAP10* |  |
| rs17334919 | Blood lipids | *KCNMB2* |  |
| rs17405722 | Blood lipids | *EIF2B3* |  |
| rs17411031 | Blood lipids | *UBE2J2* |  |
| rs17631783 | Blood lipids | *PER2* |  |
| rs1801214 | Blood lipids | *PRKCZ* |  |
| rs1899951 | Blood lipids | *RANBP2* |  |
| rs2071479 | Blood lipids | *ALDH7A1* |  |
| rs2237892 | Blood lipids | *RFC1* |  |
| rs2246012 | Blood lipids | *C4BPB* |  |
| rs2292662 | Blood lipids | *SSR1* |  |
| rs2294120 | Blood lipids | *ABCG5* |  |
| rs2296173 | Blood lipids | *SF3B5* |  |
| rs2307111 | Blood lipids | *RBM22* |  |
| rs243019 | Blood lipids | *PLRG1* |  |
| rs2493394 | Blood lipids | *NOTCH2* |  |
| rs2616132 | Blood lipids | *CLPS* |  |
| rs2633310 | Blood lipids | *MYB* |  |
| rs2857605 | Blood lipids | *NDUFS6* |  |
| rs2908282 | Blood lipids | *PRDM2* |  |
| rs302864 | Blood lipids | *AGFG1* |  |
| rs3802177 | Blood lipids | *AKR1C4* |  |
| rs3900856 | Blood lipids | *ALB* |  |
| rs459193 | Blood lipids | *NPFFR2* |  |
| rs4823182 | Blood lipids | *ID3* |  |
| rs4918796 | Blood lipids | *IL2RA* |  |
| rs5215 | Blood lipids | *PRDM2* |  |
| rs576674 | Blood lipids | *SCN7A* |  |
| rs6059662 | Blood lipids | *FBXO2* |  |
| rs6066138 | Blood lipids | *PROM1* |  |
| rs61953351 | Blood lipids | *AP4B1* |  |
| rs622217 | Blood lipids | *SH3GLB1* |  |
| rs6515236 | Blood lipids | *CCNG1* |  |
| rs6795735 | Blood lipids | *GBA* |  |
| rs7138300 | Blood lipids | *UPF2* |  |
| rs7572970 | Blood lipids | *DNAJC3* |  |
| rs77258096 | Blood lipids | *TOLLIP* |  |
| rs7756992 | Blood lipids | *GHRL* |  |
| rs7786095 | Blood lipids | *MIR625* |  |
| rs780094 | Blood lipids | *RAMP1* |  |
| rs7903146 | Blood lipids | *IL15RA* |  |
| rs8068804 | Blood lipids | *SRF* |  |
| rs853974 | Blood lipids | *GAL* |  |
| rs9369425 | Blood lipids | *TNFAIP3* |  |
| rs940904 | Blood lipids | *SETD8* |  |
| rs9844972 | Blood lipids | *SIPA1* |  |
| rs9894220 | Blood lipids | *SIRT1* |  |
| rs9911983 | Blood lipids | *STATH* |  |

T2D: type 2 diabetes; SNP: the label of single-nucleotide polymorphism. We searched the PhenoScanner and the GWAS catalog (<https://www.ebi.ac.uk/gwas>; until 12/12/2018) to check if there existed instrumental variables which showed any associations with other traits or diseases. Finally, we identified that a total of seventeen instrumental variables were previously reported to be associated with other traits or diseases. Here, we also included 70 index SNPs within 1Mb of a lipid-associated locus as blood lipid levels were previously shown to be associated with both T2D and ALS .

Table S8 Summary information for index SNPs of amyotrophic lateral sclerosis in the European population

| Chr | SNP | Position | Gene | ALLELE | BETA | SE | *P* | *N* |
| --- | --- | --- | --- | --- | --- | --- | --- | --- |
| 5 | rs10463311 | 150,410,835 | *TNIP1* | T/C | -0.0854 | 0.0156 | 4.00E-08 | 80610 |
| 9 | rs3849943 | 27,543,382 | *C9orf72* | T/C | -0.1764 | 0.0155 | 3.77E-30 | 80610 |
| 12 | rs117027576 | 57,316,603 | *SDR9C7* | T/G | -0.3164 | 0.0566 | 2.28E-08 | 80610 |
| 12 | rs118082508 | 57,318,819 | *SDR9C7* | T/C | 0.3175 | 0.0565 | 1.97E-08 | 80610 |
| 12 | rs113247976 | 57,975,700 | *KIF5A* | T/C | 0.3221 | 0.0521 | 6.43E-10 | 80610 |
| 12 | rs116900480 | 58,656,105 | *RPL21P103* | T/C | 0.3169 | 0.0513 | 6.60E-10 | 80610 |
| 12 | rs142321490 | 58,676,132 | *RPL21P103* | C/G | 0.3172 | 0.0513 | 6.15E-10 | 80610 |
| 12 | rs74654358 | 64,881,967 | *TBK1* | A/G | 0.1976 | 0.0337 | 4.66E-09 | 80610 |
| 19 | rs12973192 | 17,753,239 | *UNC13A* | C/G | -0.1205 | 0.0153 | 3.92E-15 | 80610 |
| 21 | rs75087725 | 45,753,117 | *AP001062.7* | A/C | 0.5145 | 0.0672 | 1.85E-14 | 80610 |

Chr: chromosome; SNP: the label of single-nucleotide polymorphism; Allele: effect allele and alternative allele; BETA: SNP effect size, SE: standard error of the SNP effect size; *P*, and *N* represent p value and sample size, respectively.

Table S9 Summary information for instrumental variables of type 2 diabetes in the East Asian population

| Chr | SNP | Position | Gene | ALLELE | BETA | SE | *P* | *N* | *PVE* | *F* |
| --- | --- | --- | --- | --- | --- | --- | --- | --- | --- | --- |
| 1 | rs12031188 | 51,103,268 | *FAF1* | C/T | -0.0802 | 0.0120 | 2.52E-11 | 191,764 | 2.33E-04 | 44.7 |
| 2 | rs10190313 | 647,577 | *TMEM18* | G/A | -0.0945 | 0.0150 | 2.77E-10 | 191,764 | 2.07E-04 | 39.7 |
| 2 | rs4972387 | 149,430,804 | *EPC2* | C/T | 0.0514 | 0.0088 | 5.09E-09 | 191,764 | 1.78E-04 | 34.1 |
| 2 | rs75536691 | 165,381,518 | *GRB14* | G/A | 0.1947 | 0.0318 | 9.46E-10 | 191,764 | 1.95E-04 | 37.5 |
| 3 | rs12630883 | 23,390,488 | *UBE2E2* | C/A | -0.0935 | 0.0094 | 3.41E-23 | 191,764 | 5.16E-04 | 98.9 |
| 3 | rs13092876 | 185,495,320 | *IGF2BP2* | G/A | 0.1298 | 0.0094 | 6.11E-43 | 191,764 | 9.93E-04 | 190.7 |
| 4 | rs79407053 | 1,244,218 | *CTBP1-AS1* | G/A | -0.1042 | 0.0102 | 1.20E-24 | 191,764 | 5.44E-04 | 104.4 |
| 4 | rs10011838 | 153,520,279 | *RP11-555K12.2* | G/A | -0.0745 | 0.0088 | 2.73E-17 | 191,764 | 3.74E-04 | 71.7 |
| 5 | rs2059202 | 52,097,183 | *PELO* | G/A | 0.0663 | 0.0118 | 1.84E-08 | 191,764 | 1.65E-04 | 31.6 |
| 5 | rs3135911 | 176,513,896 | *FGFR4* | C/A | 0.0552 | 0.0088 | 3.50E-10 | 191,764 | 2.05E-04 | 39.3 |
| 6 | rs2327777 | 137,293,227 | *RPL35AP3* | C/T | 0.0663 | 0.0088 | 5.34E-14 | 191,764 | 2.96E-04 | 56.8 |
| 7 | rs17168486 | 14,898,282 | *DGKB* | C/T | 0.0617 | 0.0088 | 2.34E-12 | 191,764 | 2.56E-04 | 49.2 |
| 7 | rs11514706 | 15,059,272 | *AC006045.3* | C/A | -0.0564 | 0.0088 | 1.47E-10 | 191,764 | 2.14E-04 | 41.1 |
| 7 | rs6947395 | 69,406,661 | *AUTS2* | T/A | -0.0900 | 0.0111 | 4.87E-16 | 191,764 | 3.43E-04 | 65.7 |
| 8 | rs6981607 | 41,508,168 | *NKX6-3* | C/A | -0.0859 | 0.0088 | 1.53E-22 | 191,764 | 4.97E-04 | 95.3 |
| 9 | rs2796441 | 84,308,948 | *RP11-154D17.1* | G/A | -0.0797 | 0.0089 | 2.67E-19 | 191,764 | 4.18E-04 | 80.2 |
| 10 | rs10762670 | 77,310,016 | *C10orf11* | C/T | 0.0527 | 0.0088 | 2.10E-09 | 191,764 | 1.87E-04 | 35.9 |
| 10 | rs703981 | 80,942,855 | *ZMIZ1* | G/C | -0.0667 | 0.0088 | 3.35E-14 | 191,764 | 2.99E-04 | 57.4 |
| 10 | rs78216286 | 94,420,817 | *EIF2S2P3* | C/T | 0.1036 | 0.0119 | 2.54E-18 | 191,764 | 3.95E-04 | 75.8 |
| 10 | rs12219514 | 94,466,439 | *Y_RNA* | G/A | 0.1660 | 0.0127 | 4.82E-39 | 191,764 | 8.90E-04 | 170.8 |
| 11 | rs2421897 | 35,437,863 | *SLC1A2* | G/C | -0.0788 | 0.0136 | 6.58E-09 | 191,764 | 1.75E-04 | 33.6 |
| 11 | rs11819995 | 128,389,391 | *ETS1* | C/T | 0.0599 | 0.0104 | 9.76E-09 | 191,764 | 1.73E-04 | 33.2 |
| 12 | rs11113776 | 108,613,151 | *WSCD2* | G/A | -0.0500 | 0.0088 | 1.28E-08 | 191,764 | 1.68E-04 | 32.3 |
| 13 | rs123378 | 51,088,809 | *DLEU1* | G/A | -0.0615 | 0.0105 | 4.93E-09 | 191,764 | 1.79E-04 | 34.3 |
| 13 | rs1327315 | 80,708,732 | *SPRY2* | C/T | -0.0929 | 0.0103 | 1.52E-19 | 191,764 | 4.24E-04 | 81.3 |
| 15 | rs746673 | 40,636,418 | *C15orf52* | G/A | -0.0722 | 0.0096 | 4.32E-14 | 191,764 | 2.95E-04 | 56.6 |
| 15 | rs965480 | 77,781,926 | *HMG20A* | G/A | -0.0596 | 0.0088 | 1.24E-11 | 191,764 | 2.39E-04 | 45.9 |
| 15 | rs2290203 | 91,512,067 | *AC068831.8* | G/A | 0.0557 | 0.0088 | 2.31E-10 | 191,764 | 2.09E-04 | 40.1 |
| 16 | rs1421085 | 53,800,954 | *FTO* | C/T | -0.1223 | 0.0110 | 1.06E-28 | 191,764 | 6.44E-04 | 123.6 |
| 17 | rs3094512 | 36,048,940 | *HNF1B* | G/A | -0.0634 | 0.0104 | 1.19E-09 | 191,764 | 1.94E-04 | 37.2 |
| 17 | rs11651052 | 36,102,381 | *HNF1B* | G/A | 0.1161 | 0.0095 | 2.24E-34 | 191,764 | 7.78E-04 | 149.4 |
| 18 | rs663129 | 57,838,401 | *U4* | G/A | 0.0785 | 0.0104 | 5.23E-14 | 191,764 | 2.97E-04 | 57.0 |
| 20 | rs113810779 | 42,993,328 | *HNF4A* | C/T | 0.0652 | 0.0112 | 6.09E-09 | 191,764 | 1.77E-04 | 33.9 |
| 20 | rs6021276 | 50,155,386 | *NFATC2* | C/T | 0.0538 | 0.0090 | 1.84E-09 | 191,764 | 1.86E-04 | 35.7 |

Chr: chromosome; SNP: the label of single-nucleotide polymorphism; Allele: effect allele and alternative allele; BETA: SNP effect size, SE: standard error of the SNP effect size; PVE: proportion of variance explained by the SNP; *P*, *N*, and *F* represent p value, sample size, and *F* statistic, respectively. The summary statistics results for those instrumental variables were publicly available at http://jenger.riken.jp/en/result. All the selected instruments together explain about 1.15% phenotypic variation of type 2 diabetes in the observed scale. For these instrumental variables, all the *F* statistics are above ten (ranging from 31.6 to 190.7) with an average *F* statistic of 65.1 and an overall *F* statistic of 65.6.

Table 10 Summary information for another set of instrumental variables of type 2 diabetes in the European population

| Chr | SNP | Position | Gene | ALLELE | BETA | SE | *P* | *N* | *PVE* | *F* |
| --- | --- | --- | --- | --- | --- | --- | --- | --- | --- | --- |
| 17 | rs10908278 | 36,099,952 | *HNF1B* | T/A | 0.0810 | 7.10E-03 | 6.40E-36 | 898,113 | 1.45E-04 | 130.2 |
| 7 | rs4279506 | 23,512,896 | *IGF2BP3* | G/C | 0.0580 | 9.60E-03 | 4.80E-08 | 898,113 | 4.06E-05 | 36.5 |
| 5 | rs4457053 | 76,424,949 | *ZBED3* | G/A | 0.0630 | 7.20E-03 | 8.40E-18 | 898,110 | 8.52E-05 | 76.6 |
| 6 | rs4709746 | 164,133,001 | *QKI* | C/T | 0.0580 | 9.60E-03 | 5.80E-09 | 898,114 | 4.06E-05 | 36.5 |
| 8 | rs13262861 | 41,508,577 | *ANK1* | C/A | 0.0670 | 9.50E-03 | 4.00E-12 | 898,114 | 5.54E-05 | 49.7 |
| 12 | rs77864822 | 97,848,775 | *RMST* | A/G | 0.0770 | 1.42E-02 | 1.10E-08 | 898,114 | 3.27E-05 | 29.4 |
| 4 | rs1296328 | 137,083,193 | *PABPC4L* | A/C | 0.0340 | 7.40E-03 | 3.50E-08 | 898,111 | 2.35E-05 | 21.1 |
| 11 | rs10830963 | 92,708,710 | *MTNR1B* | G/C | 0.1000 | 6.90E-03 | 4.80E-43 | 898,113 | 2.34E-04 | 210.0 |
| 4 | rs1903002 | 89,740,894 | *FAM13A* | G/C | 0.0340 | 7.40E-03 | 2.70E-08 | 898,110 | 2.35E-05 | 21.1 |
| 19 | rs7249758 | 4,948,862 | *UHRF1* | A/G | 0.0490 | 9.70E-03 | 3.40E-09 | 898,113 | 2.84E-05 | 25.5 |
| 16 | rs8046545 | 28,915,217 | *ATP2A1* | G/A | 0.0340 | 7.40E-03 | 1.90E-08 | 898,110 | 2.35E-05 | 21.1 |
| 18 | rs9957145 | 56,876,228 | *GRP* | G/A | 0.0490 | 9.70E-03 | 8.10E-09 | 898,114 | 2.84E-05 | 25.5 |
| 9 | rs2796441 | 84,308,948 | *TLE1* | G/A | 0.0630 | 7.20E-03 | 4.40E-24 | 898,113 | 8.52E-05 | 76.6 |
| 3 | rs2272163 | 77,671,721 | *ROBO2* | C/A | 0.0340 | 7.40E-03 | 9.60E-09 | 898,112 | 2.35E-05 | 21.1 |
| 18 | rs7240767 | 7,070,642 | *LAMA1* | C/T | 0.0340 | 7.40E-03 | 1.60E-08 | 898,112 | 2.35E-05 | 21.1 |
| 17 | rs61676547 | 65,892,507 | *BPTF* | C/G | 0.0530 | 7.30E-03 | 2.90E-11 | 898,114 | 5.87E-05 | 52.7 |
| 20 | rs11699802 | 48,832,135 | *CEBPB* | C/T | 0.0440 | 7.30E-03 | 1.80E-11 | 898,112 | 4.04E-05 | 36.3 |
| 3 | rs9828772 | 129,333,182 | *TMCC1* | C/G | 0.0580 | 9.60E-03 | 4.20E-08 | 898,114 | 4.06E-05 | 36.5 |
| 15 | rs13737 | 75,932,129 | *PTPN9* | G/T | 0.0440 | 7.30E-03 | 5.60E-10 | 898,114 | 4.04E-05 | 36.3 |
| 11 | rs67232546 | 128,398,938 | *ETS1* | T/C | 0.0530 | 7.30E-03 | 1.30E-11 | 898,114 | 5.87E-05 | 52.7 |
| 3 | rs3887925 | 186,665,645 | *ST6GAL1* | T/C | 0.0630 | 7.20E-03 | 3.10E-22 | 898,114 | 8.52E-05 | 76.6 |
| 15 | rs11070332 | 41,809,205 | *LTK* | A/G | 0.0490 | 4.90E-03 | 1.10E-13 | 898,112 | 1.11E-04 | 100.0 |
| 15 | rs2456530 | 53,091,553 | *ONECUT1* | T/C | 0.0580 | 9.60E-03 | 5.40E-09 | 898,114 | 4.06E-05 | 36.5 |
| 11 | rs1783541 | 65,294,799 | *MAP3K11* | T/C | 0.0630 | 7.20E-03 | 2.00E-14 | 898,112 | 8.52E-05 | 76.6 |
| 9 | rs55653563 | 97,001,682 | *ZNF169* | A/C | 0.0440 | 7.30E-03 | 2.20E-09 | 898,114 | 4.04E-05 | 36.3 |
| 9 | rs17791513 | 81,905,590 | *TLE4* | A/G | 0.1000 | 1.15E-02 | 3.10E-14 | 898,114 | 8.42E-05 | 75.6 |
| 4 | rs7669833 | 153,513,369 | *TMEM154* | T/A | 0.0530 | 7.30E-03 | 1.20E-14 | 898,113 | 5.87E-05 | 52.7 |
| 3 | rs649961 | 124,926,637 | *SLC12A8* | T/C | 0.0390 | 4.90E-03 | 9.90E-10 | 898,111 | 7.05E-05 | 63.3 |
| 9 | rs11137820 | 81,359,113 | *MTND2P8* | C/G | 0.0340 | 7.40E-03 | 2.90E-08 | 898,112 | 2.35E-05 | 21.1 |
| 5 | rs7719891 | 86,577,352 | *RASA1* | G/A | 0.0440 | 7.30E-03 | 2.40E-08 | 898,114 | 4.04E-05 | 36.3 |
| 10 | rs703972 | 80,952,826 | *ZMIZ1* | G/C | 0.0720 | 7.10E-03 | 1.70E-29 | 898,113 | 1.14E-04 | 102.8 |
| 18 | rs17684074 | 54,675,384 | *WDR7* | G/C | 0.0440 | 7.30E-03 | 2.90E-08 | 898,113 | 4.04E-05 | 36.3 |
| 15 | rs4932265 | 90,423,293 | *AP3S2* | T/C | 0.0630 | 7.20E-03 | 4.20E-20 | 898,110 | 8.52E-05 | 76.6 |
| 16 | rs2925979 | 81,534,790 | *CMIP* | T/C | 0.0530 | 7.30E-03 | 1.40E-14 | 898,114 | 5.87E-05 | 52.7 |
| 3 | rs9873618 | 170,733,076 | *SLC2A2* | G/A | 0.0630 | 7.20E-03 | 4.80E-21 | 898,113 | 8.52E-05 | 76.6 |
| 15 | rs12910825 | 91,511,260 | *PRC1* | G/A | 0.0530 | 7.30E-03 | 1.60E-15 | 898,111 | 5.87E-05 | 52.7 |
| 6 | rs9494624 | 137,300,960 | *SLC35D3* | A/G | 0.0440 | 7.30E-03 | 6.10E-09 | 898,111 | 4.04E-05 | 36.3 |
| 12 | rs2258238 | 66,221,060 | *HMGA2* | T/A | 0.1000 | 1.15E-02 | 4.50E-21 | 898,114 | 8.42E-05 | 75.6 |
| 8 | rs1561927 | 129,568,078 | *PVT1* | C/T | 0.0440 | 7.30E-03 | 1.50E-09 | 898,114 | 4.04E-05 | 36.3 |
| 1 | rs340874 | 214,159,256 | *PROX1* | C/T | 0.0630 | 7.20E-03 | 1.60E-22 | 898,114 | 8.52E-05 | 76.6 |
| 20 | rs34454109 | 51,223,594 | *TSHZ2* | A/T | 0.0440 | 7.30E-03 | 7.10E-09 | 898,112 | 4.04E-05 | 36.3 |
| 15 | rs1005752 | 77,818,128 | *HMG20A* | A/C | 0.0810 | 7.10E-03 | 2.50E-29 | 898,110 | 1.45E-04 | 130.2 |
| 5 | rs329122 | 133,864,599 | *PHF15* | A/G | 0.0390 | 4.90E-03 | 3.60E-09 | 898,114 | 7.05E-05 | 63.3 |
| 2 | rs10195252 | 165,513,091 | *GRB14/COBLL1* | T/C | 0.0680 | 4.80E-03 | 6.00E-25 | 898,114 | 2.23E-04 | 200.7 |
| 2 | rs13426680 | 158,339,550 | *CYTIP* | A/G | 0.0810 | 1.18E-02 | 6.70E-10 | 898,113 | 5.25E-05 | 47.1 |
| 7 | rs39328 | 103,444,978 | *RELN* | T/C | 0.0340 | 7.40E-03 | 3.70E-08 | 898,114 | 2.35E-05 | 21.1 |
| 7 | rs1708302 | 28,198,677 | *JAZF1* | C/T | 0.0910 | 7.00E-03 | 1.10E-48 | 898,112 | 1.88E-04 | 169.0 |
| 5 | rs1316776 | 78,430,607 | *DMGDH* | C/A | 0.0440 | 7.30E-03 | 2.60E-12 | 898,112 | 4.04E-05 | 36.3 |
| 13 | rs1359790 | 80,717,156 | *SPRY2* | G/A | 0.0810 | 7.10E-03 | 2.40E-31 | 898,113 | 1.45E-04 | 130.2 |
| 17 | rs34855406 | 40,731,411 | *MLX* | C/G | 0.0530 | 7.30E-03 | 2.30E-12 | 898,113 | 5.87E-05 | 52.7 |
| 4 | rs58730668 | 185,717,759 | *ACSL1* | T/C | 0.0670 | 9.50E-03 | 1.30E-13 | 898,114 | 5.54E-05 | 49.7 |
| 3 | rs4686471 | 187,740,899 | *LPP* | C/T | 0.0630 | 7.20E-03 | 1.70E-20 | 898,110 | 8.52E-05 | 76.6 |
| 3 | rs2872246 | 183,738,460 | *ABCC5* | A/C | 0.0340 | 7.40E-03 | 1.50E-08 | 898,113 | 2.35E-05 | 21.1 |
| 16 | rs6600191 | 295,795 | *ITFG3* | T/C | 0.0630 | 7.20E-03 | 9.30E-13 | 898,114 | 8.52E-05 | 76.6 |
| 15 | rs8037894 | 62,394,264 | *C2CD4A/B* | G/C | 0.0440 | 7.30E-03 | 2.60E-13 | 898,111 | 4.04E-05 | 36.3 |
| 22 | rs5758223 | 41,489,920 | *EP300* | A/G | 0.0390 | 4.90E-03 | 3.80E-08 | 898,112 | 7.05E-05 | 63.3 |
| 12 | rs2197973 | 95,928,560 | *USP44* | T/C | 0.0340 | 7.40E-03 | 3.60E-08 | 898,111 | 2.35E-05 | 21.1 |
| 3 | rs6780171 | 185,503,456 | *IGF2BP2* | A/T | 0.1310 | 9.00E-03 | 9.00E-56 | 898,111 | 2.36E-04 | 211.9 |
| 6 | rs3798519 | 50,788,778 | *TFAP2B* | C/A | 0.0580 | 9.60E-03 | 2.60E-12 | 898,114 | 4.06E-05 | 36.5 |
| 15 | rs7178762 | 63,871,292 | *USP3* | C/T | 0.0390 | 4.90E-03 | 5.40E-10 | 898,114 | 7.05E-05 | 63.3 |
| 18 | rs72926932 | 53,050,646 | *TCF4* | C/A | 0.0900 | 1.17E-02 | 1.00E-14 | 898,114 | 6.59E-05 | 59.2 |
| 14 | rs17836088 | 79,932,041 | *NRXN3* | C/G | 0.0580 | 9.60E-03 | 6.70E-14 | 898,113 | 4.06E-05 | 36.5 |
| 7 | rs11496066 | 102,486,254 | *FBXL13* | T/C | 0.0770 | 1.42E-02 | 1.10E-08 | 898,114 | 3.27E-05 | 29.4 |
| 2 | rs35999103 | 147,861,633 | *PABPC1P2* | T/C | 0.0490 | 9.70E-03 | 9.70E-09 | 898,113 | 2.84E-05 | 25.5 |
| 3 | rs2581787 | 53,127,677 | *RFT1* | T/G | 0.0340 | 7.40E-03 | 2.40E-08 | 898,112 | 2.35E-05 | 21.1 |
| 4 | rs6821438 | 95,091,911 | *SMARCAD1* | A/G | 0.0440 | 7.30E-03 | 4.00E-11 | 898,112 | 4.04E-05 | 36.3 |
| 16 | rs862320 | 69,651,866 | *NFAT5* | C/T | 0.0440 | 7.30E-03 | 3.90E-11 | 898,112 | 4.04E-05 | 36.3 |
| 3 | rs7629630 | 168,218,841 | *EGFEM1P* | A/T | 0.0490 | 9.70E-03 | 2.50E-08 | 898,114 | 2.84E-05 | 25.5 |
| 18 | rs62080313 | 36,278,709 | *COMMD9* | C/T | 0.0580 | 9.60E-03 | 1.00E-08 | 898,113 | 4.06E-05 | 36.5 |
| 4 | rs28819812 | 157,652,753 | *PDGFC* | C/A | 0.0440 | 7.30E-03 | 2.20E-08 | 608,289 | 5.97E-05 | 36.3 |
| 5 | rs702634 | 53,271,420 | *ARL15* | A/G | 0.0530 | 7.30E-03 | 7.70E-14 | 898,113 | 5.87E-05 | 52.7 |
| 1 | rs12048743 | 205,114,873 | *DSTYK* | G/C | 0.0390 | 4.90E-03 | 3.50E-09 | 898,113 | 7.05E-05 | 63.3 |
| 18 | rs523288 | 57,848,369 | *MC4R* | T/A | 0.0530 | 7.30E-03 | 7.60E-13 | 898,113 | 5.87E-05 | 52.7 |
| 10 | rs2280141 | 124,193,181 | *PLEKHA1* | T/G | 0.0440 | 7.30E-03 | 1.40E-13 | 898,112 | 4.04E-05 | 36.3 |
| 16 | rs1421085 | 53,800,954 | *FTO* | C/T | 0.1270 | 6.70E-03 | 3.10E-84 | 898,113 | 4.00E-04 | 359.3 |
| 5 | rs3811978 | 52,100,489 | *ITGA1* | G/A | 0.0530 | 7.30E-03 | 7.70E-11 | 898,112 | 5.87E-05 | 52.7 |
| 11 | rs2767036 | 34,982,148 | *PDHX* | C/A | 0.0340 | 7.40E-03 | 3.30E-08 | 898,111 | 2.35E-05 | 21.1 |
| 13 | rs9537803 | 58,366,634 | *PCDH17* | C/T | 0.0440 | 7.30E-03 | 4.60E-08 | 898,111 | 4.04E-05 | 36.3 |
| 7 | rs10228066 | 15,063,569 | *DGKB* | T/C | 0.0720 | 7.10E-03 | 1.10E-28 | 898,111 | 1.14E-04 | 102.8 |
| 10 | rs10882101 | 94,462,427 | *HHEX/IDE* | T/C | 0.0580 | 9.60E-03 | 1.40E-08 | 898,113 | 4.06E-05 | 36.5 |
| 9 | rs505922 | 136,149,229 | *ABO* | C/T | 0.0440 | 7.30E-03 | 3.90E-12 | 898,114 | 4.04E-05 | 36.3 |
| 13 | rs963740 | 51,096,095 | *DLEU1* | A/T | 0.0390 | 4.90E-03 | 2.10E-08 | 898,113 | 7.05E-05 | 63.3 |
| 8 | rs10097617 | 95,961,626 | *TP53INP1* | T/C | 0.0440 | 7.30E-03 | 3.30E-11 | 898,113 | 4.04E-05 | 36.3 |
| 14 | rs62007683 | 103,894,071 | *MARK3* | G/T | 0.0340 | 7.40E-03 | 3.10E-08 | 898,112 | 2.35E-05 | 21.1 |
| 1 | rs539515 | 177,889,025 | *SEC16B* | C/A | 0.0530 | 7.30E-03 | 1.60E-10 | 898,112 | 5.87E-05 | 52.7 |
| 2 | rs2972144 | 227,101,411 | *IRS1* | G/A | 0.0910 | 7.00E-03 | 2.10E-46 | 898,111 | 1.88E-04 | 169.0 |
| 1 | rs2820446 | 219,748,818 | *LYPLAL1* | C/G | 0.0530 | 7.30E-03 | 3.30E-16 | 898,114 | 5.87E-05 | 52.7 |
| 2 | rs2249105 | 65,287,896 | *CEP68* | A/G | 0.1000 | 1.15E-02 | 2.20E-14 | 898,114 | 8.42E-05 | 75.6 |
| 3 | rs35352848 | 23,455,582 | *UBE2E2* | T/C | 0.0670 | 9.50E-03 | 1.30E-17 | 898,114 | 5.54E-05 | 49.7 |
| 15 | rs4776970 | 68,080,886 | *MAP2K5* | A/T | 0.0390 | 4.90E-03 | 5.00E-09 | 898,114 | 7.05E-05 | 63.3 |

Chr: chromosome; SNP: the label of single-nucleotide polymorphism; Allele: effect allele and alternative allele; BETA: SNP effect size, SE: standard error of the SNP effect size; PVE: proportion of variance explained by the SNP; *P*, *N*, and *F* represent p value, sample size, and *F* statistic, respectively. Using the same selection procedure described in Fig 1, after removing potential instrumental outliers, we obtained a total of 90 instruments with high imputation score (>0.95). All the selected instruments together explain about 0.62% phenotypic variation of type 2 diabetes in the observed scale. For these instrumental variables, all the *F* statistics are above ten (ranging from 21.1 to 359.3) with an average *F* statistic of 61.5 and an overall *F* statistic of 62.2. We had to use the MR-Egger regression instead of standard IVW methods for this analysis, because the intercept in the MR-Egger regression is estimated to be statistically significantly different from zero (estimate = 0.010; 95% CI 0.001 - 0.020, *p* = 0.035). The estimated causal effect of T2D on ALS in the MR-Egger regression is 0.86 (95% CI 0.73 - 1.01, *p* = 0.068), which is marginally significant at the level of 0.05 and consistent with our main results. Using weighted median-based method we till gained a negative but nonsignificant causal effect of T2D on ALS (odd ratio = 0.97, 95% CI 0.89 - 1.06, *p* = 0.494). Note that due to the lower statistical power of MR-Egger regression and weighted median-based method compared to standard IVW methods, the significance level obtained using this new set of instruments is not as strong as the main results in our study.

Table 11 Estimated causal effects and 95% confidence intervals for type 2 diabetes on amyotrophic lateral sclerosis in the leave-one-out analysis by removing one instruments each time using the fixed-effects inverse-variance weighted method

| Instruments | OR | SE |  | 95% CI | |  | *P* |
| --- | --- | --- | --- | --- | --- | --- | --- |
|  |  |  |  | Lower | Upper |  |  |
| rs12088739 | 0.935 | 0.030 |  | 0.881 | 0.991 |  | 0.024 |
| rs340874 | 0.931 | 0.030 |  | 0.878 | 0.987 |  | 0.017 |
| rs2820426 | 0.932 | 0.030 |  | 0.879 | 0.989 |  | 0.019 |
| rs348330 | 0.932 | 0.030 |  | 0.879 | 0.988 |  | 0.018 |
| rs2867125 | 0.940 | 0.030 |  | 0.886 | 0.996 |  | 0.038 |
| rs1009358 | 0.939 | 0.030 |  | 0.886 | 0.996 |  | 0.037 |
| rs13389219 | 0.935 | 0.030 |  | 0.882 | 0.992 |  | 0.026 |
| rs2972144 | 0.943 | 0.030 |  | 0.889 | 1.001 |  | 0.052 |
| rs7561798 | 0.936 | 0.030 |  | 0.883 | 0.992 |  | 0.026 |
| rs1496653 | 0.930 | 0.030 |  | 0.877 | 0.986 |  | 0.016 |
| rs4472028 | 0.932 | 0.030 |  | 0.879 | 0.989 |  | 0.019 |
| rs11925227 | 0.935 | 0.030 |  | 0.882 | 0.991 |  | 0.025 |
| rs7651090 | 0.934 | 0.031 |  | 0.880 | 0.992 |  | 0.027 |
| rs3887925 | 0.934 | 0.030 |  | 0.881 | 0.990 |  | 0.022 |
| rs6808574 | 0.937 | 0.030 |  | 0.883 | 0.993 |  | 0.029 |
| rs17086692 | 0.936 | 0.030 |  | 0.883 | 0.993 |  | 0.027 |
| rs993380 | 0.928 | 0.030 |  | 0.875 | 0.984 |  | 0.013 |
| rs7674212 | 0.932 | 0.030 |  | 0.879 | 0.988 |  | 0.019 |
| rs7685296 | 0.930 | 0.030 |  | 0.877 | 0.986 |  | 0.015 |
| rs735949 | 0.932 | 0.030 |  | 0.879 | 0.988 |  | 0.018 |
| rs1061813 | 0.936 | 0.030 |  | 0.883 | 0.992 |  | 0.027 |
| rs4865796 | 0.929 | 0.030 |  | 0.876 | 0.985 |  | 0.014 |
| rs6878122 | 0.937 | 0.030 |  | 0.883 | 0.993 |  | 0.029 |
| rs7729395 | 0.940 | 0.030 |  | 0.887 | 0.997 |  | 0.039 |
| rs72892910 | 0.936 | 0.030 |  | 0.883 | 0.993 |  | 0.027 |
| rs17168486 | 0.937 | 0.030 |  | 0.883 | 0.994 |  | 0.030 |
| rs2191348 | 0.928 | 0.030 |  | 0.875 | 0.984 |  | 0.013 |
| rs849135 | 0.933 | 0.030 |  | 0.878 | 0.990 |  | 0.022 |
| rs2299383 | 0.935 | 0.030 |  | 0.882 | 0.991 |  | 0.024 |
| rs13234269 | 0.938 | 0.030 |  | 0.885 | 0.995 |  | 0.033 |
| rs7841082 | 0.930 | 0.030 |  | 0.877 | 0.986 |  | 0.015 |
| rs12681990 | 0.940 | 0.030 |  | 0.886 | 0.997 |  | 0.038 |
| rs516946 | 0.926 | 0.030 |  | 0.873 | 0.982 |  | 0.011 |
| rs7845219 | 0.936 | 0.030 |  | 0.883 | 0.992 |  | 0.026 |
| rs1758632 | 0.945 | 0.030 |  | 0.892 | 1.002 |  | 0.060 |
| rs17791483 | 0.936 | 0.030 |  | 0.882 | 0.992 |  | 0.026 |
| rs2796441 | 0.934 | 0.030 |  | 0.881 | 0.991 |  | 0.024 |
| rs10114341 | 0.933 | 0.030 |  | 0.880 | 0.990 |  | 0.021 |
| rs687621 | 0.935 | 0.030 |  | 0.882 | 0.991 |  | 0.024 |
| rs753270 | 0.932 | 0.030 |  | 0.879 | 0.989 |  | 0.019 |
| rs7923866 | 0.937 | 0.030 |  | 0.883 | 0.995 |  | 0.033 |
| rs11591741 | 0.937 | 0.030 |  | 0.883 | 0.993 |  | 0.028 |
| rs2421016 | 0.930 | 0.030 |  | 0.877 | 0.986 |  | 0.015 |
| rs7929543 | 0.938 | 0.030 |  | 0.885 | 0.995 |  | 0.032 |
| rs10830963 | 0.931 | 0.030 |  | 0.877 | 0.987 |  | 0.017 |
| rs7931302 | 0.937 | 0.030 |  | 0.884 | 0.994 |  | 0.031 |
| rs67232546 | 0.932 | 0.030 |  | 0.879 | 0.988 |  | 0.018 |
| rs2261181 | 0.930 | 0.030 |  | 0.877 | 0.987 |  | 0.016 |
| rs1480474 | 0.940 | 0.030 |  | 0.886 | 0.996 |  | 0.037 |
| rs11107116 | 0.938 | 0.030 |  | 0.885 | 0.994 |  | 0.031 |
| rs825476 | 0.932 | 0.030 |  | 0.879 | 0.988 |  | 0.018 |
| rs963740 | 0.938 | 0.030 |  | 0.884 | 0.994 |  | 0.032 |
| rs1359790 | 0.928 | 0.030 |  | 0.875 | 0.985 |  | 0.013 |
| rs7144011 | 0.931 | 0.030 |  | 0.878 | 0.987 |  | 0.017 |
| rs4502156 | 0.933 | 0.030 |  | 0.880 | 0.989 |  | 0.020 |
| rs982077 | 0.937 | 0.030 |  | 0.884 | 0.994 |  | 0.029 |
| rs7177055 | 0.933 | 0.030 |  | 0.880 | 0.989 |  | 0.020 |
| rs4932143 | 0.934 | 0.030 |  | 0.881 | 0.991 |  | 0.023 |
| rs12910825 | 0.928 | 0.030 |  | 0.875 | 0.984 |  | 0.012 |
| rs9940149 | 0.934 | 0.030 |  | 0.881 | 0.990 |  | 0.023 |
| rs7185735 | 0.944 | 0.031 |  | 0.889 | 1.002 |  | 0.057 |
| rs244415 | 0.938 | 0.030 |  | 0.885 | 0.995 |  | 0.032 |
| rs2925979 | 0.933 | 0.030 |  | 0.880 | 0.989 |  | 0.021 |
| rs11651755 | 0.934 | 0.030 |  | 0.881 | 0.991 |  | 0.024 |
| rs7240767 | 0.932 | 0.030 |  | 0.879 | 0.989 |  | 0.019 |
| rs12970134 | 0.934 | 0.030 |  | 0.881 | 0.990 |  | 0.022 |
| rs4810426 | 0.934 | 0.030 |  | 0.881 | 0.990 |  | 0.022 |

OR: odds ratio; SE: standard error; CI: confidence interval.

# References

1. Sun Y, Lu C-J, Chen R-C, Hou W-H, Li C-Y: **Risk of Amyotrophic Lateral Sclerosis in Patients With Diabetes: A Nationwide Population-Based Cohort Study**. *J Epidemiol* 2015, **25**(6):445-451.

2. D'Ovidio F, d'Errico A, Carna P, Calvo A, Costa G, Chio A: **The role of pre-morbid diabetes on developing amyotrophic lateral sclerosis**. *Eur J Neurol* 2018, **25**(1):164-170.

3. Moglia C, Calvo A, Canosa A, Bertuzzo D, Cugnasco P, Solero L, Grassano M, Bersano E, Cammarosano S, Manera U: **Influence of arterial hypertension, type 2 diabetes and cardiovascular risk factors on ALS outcome: a population-based study**. *Amyotrophic Lateral Sclerosis and Frontotemporal Degeneration* 2017, **18**(7-8):590-597.

4. Visser AE, Seelen M, Hulsbergen A, de Graaf J, van der Kooi AJ, Raaphorst J, Veldink JH, van den Berg LH: **Exploring the fitness hypothesis in ALS: a population-based case-control study of parental cause of death and lifespan**. *J Neurol Neurosurg Psychiatry* 2017, **88**(7):550-556.

5. Hollinger SK, Okosun IS, Mitchell CS: **Antecedent Disease and Amyotrophic Lateral Sclerosis: What Is Protecting Whom?** *Frontiers in Neurology* 2016, **7**.

6. Mitchell CS, Hollinger SK, Goswami SD, Polak MA, Lee RH, Glass JD: **Antecedent Disease Is Less Prevalent in Amyotrophic Lateral Sclerosis**. *Neurodegenerative Diseases* 2015, **15**(2):109-113.

7. Kioumourtzoglou M-A, Rotem RS, Seals RM, Gredal O, Hansen J, Weisskopf MG: **Diabetes Mellitus, Obesity, and Diagnosis of Amyotrophic Lateral Sclerosis: A Population-Based Study**. *JAMA Neurology* 2015, **72**(8):905-911.

8. Mariosa D, Fang F: **Response to the letter ‘Type 2 diabetes and amyotrophic lateral sclerosis’**. *Eur J Neurol* 2016, **23**(4):e26-e26.

9. Mariosa D, Kamel F, Bellocco R, Ye W, Fang F: **Association between diabetes and amyotrophic lateral sclerosis in Sweden**. *Eur J Neurol* 2015, **22**(11):1436-1442.

10. Seelen M, van Doormaal PTC, Visser AE, Huisman MHB, Roozekrans MHJ, de Jong SW, van der Kooi AJ, de Visser M, Voermans NC, Veldink JH *et al*: **Prior medical conditions and the risk of amyotrophic lateral sclerosis**. *J Neurol* 2014, **261**(10):1949-1956.

11. Turner MR, Goldacre R, Ramagopalan S, Talbot K, Goldacre MJ: **Autoimmune disease preceding amyotrophic lateral sclerosis An epidemiologic study**. *Neurology* 2013, **81**(14):1222-1225.

12. Körner S, Kollewe K, Ilsemann J, Müller-Heine A, Dengler R, Krampfl K, Petri S: **Prevalence and prognostic impact of comorbidities in amyotrophic lateral sclerosis**. *Eur J Neurol* 2012, **20**(4):647-654.

13. Armon C, Kurland LT, O'Brien PC, Mulder DW: **Antecedent medical diseases in patients with amyotrophic lateral sclerosis. A population-based case-controlled study in Rochester, Minn, 1925 through 1987**. *Arch Neurol* 1991, **48**.

14. Xue A, Wu Y, Zhu Z, Zhang F, Kemper KE, Zheng Z, Yengo L, Lloyd-Jones LR, Sidorenko J, Wu Y *et al*: **Genome-wide association analyses identify 143 risk variants and putative regulatory mechanisms for type 2 diabetes**. *Nat Commun* 2018, **9**(1):2941.

15. Locke AE, Kahali B, Berndt SI, Justice AE, Pers TH, Day FR, Powell C, Vedantam S, Buchkovich ML, Yang J: **Genetic studies of body mass index yield new insights for obesity biology**. *Nature* 2015, **518**(7538):197-206.

16. Scott RA, Lagou V, Welch RP, Wheeler E, Montasser ME, Luan Ja, Magi R, Strawbridge RJ, Rehnberg E, Gustafsson S *et al*: **Large-scale association analyses identify new loci influencing glycemic traits and provide insight into the underlying biological pathways**. *Nat Genet* 2012, **44**(9):991-1005.

17. Wheeler E, Leong A, Liu C-T, Hivert M-F, Strawbridge RJ, Podmore C, Li M, Yao J, Sim X, Hong J *et al*: **Impact of common genetic determinants of Hemoglobin A1c on type 2 diabetes risk and diagnosis in ancestrally diverse populations: A transethnic genome-wide meta-analysis**. *PLoS Med* 2017, **14**(9):e1002383.

18. Prokopenko I, Poon W, Mägi R, Prasad B R, Salehi SA, Almgren P, Osmark P, Bouatia-Naji N, Wierup N, Fall T *et al*: **A Central Role for GRB10 in Regulation of Islet Function in Man**. *PLoS Genet* 2014, **10**(4):e1004235.

19. Manning AK, Hivert M-F, Scott RA, Grimsby JL, Bouatia-Naji N, Chen H, Rybin D, Liu C-T, Bielak LF, Prokopenko I *et al*: **A genome-wide approach accounting for body mass index identifies genetic variants influencing fasting glycemic traits and insulin resistance**. *Nat Genet* 2012, **44**(6):659-669.

20. Wood AR, Jonsson A, Jackson AU, Wang N, van Leewen N, Palmer ND, Kobes S, Deelen J, Boquete-Vilarino L, Paananen J: **A genome-wide association study of IVGTT-based measures of first phase insulin secretion refines the underlying physiology of type 2 diabetes variants**. *Diabetes* 2017, **66**(8):2296-2309.

21. Choquet H, Thai KK, Yin J, Hoffmann TJ, Kvale MN, Banda Y, Schaefer C, Risch N, Nair KS, Melles R: **A large multi-ethnic genome-wide association study identifies novel genetic loci for intraocular pressure**. *Nat Commun* 2017, **8**(1):2108.

22. Wu Y, Gao H, Li H, Tabara Y, Nakatochi M, Chiu Y-F, Park EJ, Wen W, Adair LS, Borja JB *et al*: **A meta-analysis of genome-wide association studies for adiponectin levels in East Asians identifies a novel locus near WDR11-FGFR2**. *Hum Mol Genet* 2014, **23**(4):1108-1119.

23. Speliotes EK, Willer CJ, Berndt SI, Monda KL, Thorleifsson G, Jackson AU, Allen HL, Lindgren CM, Luan Ja, Mägi R: **Association analyses of 249,796 individuals reveal 18 new loci associated with body mass index**. *Nat Genet* 2010, **42**(11):937-948.

24. Spracklen CN, Chen P, Kim YJ, Wang X, Cai H, Li S, Long J, Wu Y, Wang YX, Takeuchi F *et al*: **Association analyses of East Asian individuals and trans-ancestry analyses with European individuals reveal new loci associated with cholesterol and triglyceride levels**. *Hum Mol Genet* 2017, **26**(9):1770-1784.

25. Teslovich TM, Musunuru K, Smith AV, Edmondson AC, Stylianou IM, Koseki M, Pirruccello JP, Ripatti S, Chasman DI, Willer CJ *et al*: **Biological, clinical and population relevance of 95 loci for blood lipids**. *Nature* 2010, **466**(7307):707-713.

26. JC C, P E, D Z, W Z, Y L: **Common genetic variation near MC4R is associated with waist circumference and insulin resistance**. *Nat Genet* 2008, **40**:716.

27. Global Lipids Genetics C, Willer CJ, Schmidt EM, Sengupta S, Peloso GM, Gustafsson S, Kanoni S, Ganna A, Chen J, Buchkovich ML *et al*: **Discovery and refinement of loci associated with lipid levels**. *Nat Genet* 2013, **45**(11):1274-1283.

28. Wu B, Pankow JS: **Fast and Accurate Genome-Wide Association Test of Multiple Quantitative Traits**. *Comput Math Methods Med* 2018.

29. Rasmussen-Torvik LJ, Guo X, Bowden DW, Bertoni AG, Sale MM, Yao J, Bluemke DA, Goodarzi MO, Chen YI, Vaidya D *et al*: **Fasting Glucose GWAS Candidate Region Analysis Across Ethnic Groups in the Multiethnic Study of Atherosclerosis (MESA)**. *Genet Epidemiol* 2012, **36**(4):384-391.

30. Evangelou E, Warren HR, Mosen-Ansorena D, Mifsu B, Pazoki R, Gao H, Ntritsos G, Dimou N, Cabrer CP, Karaman I *et al*: **Genetic analysis of over 1 million people identifies 535 new loci associated with blood pressure traits**. *Nat Genet* 2018, **50**(10):1412-1425.

31. Kanai M, Akiyama M, Takahashi A, Matoba N, Momozawa Y, Ikeda M, Iwata N, Ikegawa S, Hirata M, Matsuda K *et al*: **Genetic analysis of quantitative traits in the Japanese population links cell types to complex human diseases**. *Nat Genet* 2018, **50**(3):390-400.

32. Smith NL, Huffman JE, Strachan DP, Huang J, Dehghan A, Trompet S, Lopez LM, Shin S-Y, Baumert J, Vitart V *et al*: **Genetic predictors of fibrin D-dimer levels in healthy adults**. *Circulation* 2011, **123**(17):1864-1872.

33. Zhou H, Tian X, Tufro A, Moeckel G, Ishibe S, Goodwin J: **Loss of the podocyte glucocorticoid receptor exacerbates proteinuria after injury**. *Scientific Reports* 2017, **7**(1):9833.

34. Klarin D, Damrauer SM, Cho K, Sun YV, Teslovich TM, Honerlaw J, Gagnon DR, Du Vall SL, Li J, Peloso GM *et al*: **Genetics of blood lipids among similar to 300,000 multi-ethnic participants of the Million Veteran Program**. *Nat Genet* 2018, **50**(11):1514-1523.

35. Weedon MN, Lango H, Lindgren CM, Wallace C, Evans DM, Mangino M, Freathy RM, Perry JRB, Stevens S, Hall AS *et al*: **Genome-wide association analysis identifies 20 loci that influence adult height**. *Nat Genet* 2008, **40**(5):575-583.

36. Ratman D, Mylka V, Bougarne N, Pawlak M, Caron S, Hennuyer N, Paumelle R, De Cauwer L, Thommis J, Rider MH *et al*: **Chromatin recruitment of activated AMPK drives fasting response genes co-controlled by GR and PPARα**. *Nucleic Acids Res* 2016, **44**(22):10539-10553.

37. Kettunen J, Tukiainen T, Sarin AP, Ortega-Alonso A, Tikkanen E, Lyytikäinen LP: **Genome-wide association study identifies multiple loci influencing human serum metabolite levels**. *Nat Genet* 2012, **44**(3):269-276.

38. Annunziato AT, Hansen JC: **Role of histone acetylation in the assembly and modulation of chromatin structures**. *Gene Expr* 2018, **9**(1-2):37-61.

39. Goes FS, McGrath J, Avramopoulos D, Wolyniec P, Pirooznia M, Ruczinski I, Nestadt G, Kenny EE, Vacic V, Peters I *et al*: **Genome-wide association study of schizophrenia in Ashkenazi Jews**. *American Journal of Medical Genetics Part B-Neuropsychiatric Genetics* 2015, **168**(8):649-659.

40. Dupuis J, Langenberg C, Prokopenko I, Saxena R, Soranzo N, Jackson AU, Wheeler E, Glazer NL, Bouatia-Naji N, Gloyn AL: **New genetic loci implicated in fasting glucose homeostasis and their impact on type 2 diabetes risk**. *Nat Genet* 2010, **42**(2):105-116.

41. Thorleifsson G, Walters GB, Gudbjartsson DF, Steinthorsdottir V, Sulem P, Helgadottir A: **Genome-wide association yields new sequence variants at seven loci that associate with measures of obesity**. *Nat Genet* 2008, **41**.

42. Horikoshi M, Beaumont RN, Day FR, Warrington NM, Kooijman MN, Fernandez-Tajes J, Feenstra B, van Zuydam NR, Gaulton KJ, Grarup N *et al*: **Genome-wide associations for birth weight and correlations with adult disease**. *Nature* 2016, **538**(7624):248-252.

43. Berndt SI, Gustafsson S, Magi R, Ganna A, Wheeler E, Feitosa MF, Justice AE, Monda KL, Croteau-Chonka DC, Day FR *et al*: **Genome-wide meta-analysis identifies 11 new loci for anthropometric traits and provides insights into genetic architecture**. *Nat Genet* 2013, **45**(5):501-512.

44. Justice AE, Winkler TW, Feitosa MF, Graff M, Fisher VA, Young K, Barata L, Deng X, Czajkowski J, Hadley D *et al*: **Genome-wide meta-analysis of 241,258 adults accounting for smoking behaviour identifies novel loci for obesity traits**. *Nat Commun* 2017, **8**:14977.

45. Graff M, Scott RA, Justice AE, Young KL, Feitosa MF, Barata L, Winkler TW, Chu AY, Mahajan A, Hadley D *et al*: **Genome-wide physical activity interactions in adiposity ― A meta-analysis of 200,452 adults**. *PLoS Genet* 2017, **13**(4):e1006528.

46. Sun BB, Maranville JC, Peters JE, Stacey D, Staley JR, Blackshaw J, Burgess S, Jiang T, Paige E, Surendran P *et al*: **Genomic atlas of the human plasma proteome**. *Nature* 2018, **558**(7708):73-79.

47. Lango Allen H, Estrada K, Lettre G, Berndt SI, Weedon MN, Rivadeneira F, Willer CJ, Jackson AU, Vedantam S, Raychaudhuri S *et al*: **Hundreds of variants clustered in genomic loci and biological pathways affect human height**. *Nature* 2010, **467**.

48. Phelan CM, Kuchenbaecker KB, Tyrer JP, Kar SP, Lawrenson K, Winham SJ, Dennis J, Pirie A, Riggan MJ, Chornokur G *et al*: **Identification of 12 new susceptibility loci for different histotypes of epithelial ovarian cancer**. *Nat Genet* 2017, **49**(5):680-+.

49. He M, Xu M, Zhang B, Liang J, Chen P, Lee J-Y, Johnson TA, Li H, Yang X, Dai J *et al*: **Meta-analysis of genome-wide association studies of adult height in East Asians identifies 17 novel loci**. *Hum Mol Genet* 2015, **24**(6):1791-1800.

50. Chu AY, Deng X, Fisher VA, Drong A, Zhang Y, Feitosa MF, Liu C-T, Weeks O, Choh AC, Duan Q *et al*: **Multiethnic genome-wide meta-analysis of ectopic fat depots identifies loci associated with adipocyte development and differentiation**. *Nat Genet* 2017, **49**(1):125-130.

51. Shungin D, Winkler TW, Croteau-Chonka DC, Ferreira T, Lockes AE, Maegi R, Strawbridge RJ, Pers TH, Fischer K, Justice AE *et al*: **New genetic loci link adipose and insulin biology to body fat distribution**. *Nature* 2015, **518**(7538):187-U378.

52. Comuzzie AG, Cole SA, Laston SL, Voruganti VS, Haack K, Gibbs RA, Butte NF: **Novel genetic loci identified for the pathophysiology of childhood obesity in the Hispanic population**. *PLoS ONE* 2012, **7**(12):e51954.

53. Dastani Z, Hivert M-F, Timpson N, Perry JRB, Yuan X, Scott RA, Henneman P, Heid IM, Kizer JR, Lyytikäinen L-P *et al*: **Novel Loci for Adiponectin Levels and Their Influence on Type 2 Diabetes and Metabolic Traits: A Multi-Ethnic Meta-Analysis of 45,891 Individuals**. *PLoS Genet* 2012, **8**(3):e1002607.

54. Astle WJ, Elding H, Jiang T, Allen D, Ruklisa D, Mann AL, Mead D, Bouman H, Riveros-Mckay F, Kostadima MA *et al*: **The Allelic Landscape of Human Blood Cell Trait Variation and Links to Common Complex Disease**. *Cell* 2016, **167**(5):1415-1429.e1419.

55. Winkler TW, Justice AE, Graff M, Barata L, Feitosa MF, Chu S, Czajkowski J, Esko T, Fall T, Kilpeläinen TO *et al*: **The Influence of Age and Sex on Genetic Associations with Adult Body Size and Shape: A Large-Scale Genome-Wide Interaction Study**. *PLoS Genet* 2015, **11**(10):e1005378.

56. Surendran P, Drenos F, Young R, Warren H, Cook JP, Manning AK, Grarup N, Sim X, Barnes DR, Witkowska K: **Trans-ancestry meta-analyses identify rare and common variants associated with blood pressure and hypertension**. *Nat Genet* 2016, **48**.

57. Prokopenko I, Langenberg C, Florez JC, Saxena R, Soranzo N, Thorleifsson G, Loos RJF, Manning AK, Jackson AU, Aulchenko Y *et al*: **Variants in MTNR1B influence fasting glucose levels**. *Nat Genet* 2009, **41**(1):77-81.

58. Staley JR, Blackshaw J, Kamat MA, Ellis S, Surendran P, Sun BB, Paul DS, Freitag D, Burgess S, Danesh J *et al*: **PhenoScanner: a database of human genotype-phenotype associations**. *Bioinformatics* 2016, **32**(20):3207-3209.

59. Zeng P, Zhou X: **Causal effects of blood lipids on amyotrophic lateral sclerosis: a Mendelian randomization study**. *Hum Mol Genet* 2019, **28**(4):688-697.

60. Bandres-Ciga S, Noyce AJ, Hemani G, Nicolas A, Calvo A, Mora G, The IC, The International ALSGC, Tienari PJ, Stone DJ *et al*: **Shared polygenic risk and causal inferences in amyotrophic lateral sclerosis**. *Ann Neurol* 2019, **85**(4):470-481.

61. Nicolas A, Kenna KP, Renton AE, Ticozzi N, Faghri F, Chia R, Dominov JA, Kenna BJ, Nalls MA, Keagle P *et al*: **Genome-wide Analyses Identify KIF5A as a Novel ALS Gene**. *Neuron* 2018, **97**(6):1268-1283.e1266.

62. Suzuki K, Akiyama M, Ishigaki K, Kanai M, Hosoe J, Shojima N, Hozawa A, Kadota A, Kuriki K, Naito M *et al*: **Identification of 28 new susceptibility loci for type 2 diabetes in the Japanese population**. *Nat Genet* 2019, **51**(3):379-386.

63. Mahajan A, Taliun D, Thurner M, Robertson NR, Torres JM, Rayner NW, Payne AJ, Steinthorsdottir V, Scott RA, Grarup N *et al*: **Fine-mapping type 2 diabetes loci to single-variant resolution using high-density imputation and islet-specific epigenome maps**. *Nat Genet* 2018, **50**(11):1505-1513.
