# Supplementary material for: Causal association of type 2 diabetes with amyotrophic lateral sclerosis: new evidence from Mendelian randomization using GWAS summary statistics
Source: BMC Med. 2019 Dec 4;17:225. doi: 10.1186/s12916-019-1448-9 (PMC6892209; doi:10.1186/s12916-019-1448-9)
Supplement: Supplementary file 2 — Additional file 2. An Overview of the Mendelian Randomization Method. [file 12916_2019_1448_MOESM2_ESM.doc]

**Additional file 2**

# An Overview of the Mendelian Randomization Method

## Assumptions of Mendelian randomization

In the era of genome-wide association studies (GWAS), Mendelian randomization (MR) employs single nucleotide polymorphism (SNP) as instrumental variable to investigate the causal relation between an exposure and an outcome in observational studies . The MR approach borrows an intuitive idea that a genetic variant associated with the exposure can influence the outcome via the pathway of the exposure if the exposure is causally associated with the outcome.

To perform a valid causal inference in the MR analysis, the SNP that is used to be instrumental variable needs to satisfy three important assumptions (Additional file 1: Figure S2A):

The first assumption requires that the selected SNP is strongly associated with the exposure of interest. It is referred to as ***the relevance assumption***. Typically, to guarantee the validity of this assumption, we choose the independent index SNP at the genome-wide significance level (*p* < 5.00E-8) to be instrument and further employ the *F* statistic (also known as the Cragg-Donald statistic ) to quantitatively measure its strength. Note that the relevance assumption, however, does not require the SNP to be necessarily causally associated with the exposure (it is in fact rather difficult to determine the causality for an associated SNP with the outcome under consideration in GWAS). It can be a proxy for the true causal genetic variant. Therefore, any SNP that is in high linkage disequilibrium with the causal one which satisfies the assumptions of MR can be applied as an instrumental variable.

The second assumption is referred to as ***the independence assumption***, which states that the selected SNP cannot be associated with any other confounders that are associated with both the exposure and outcome.

The third assumption requires that the selected SNP is only associated with the outcome by the path of the exposure and thus does not have a pleiotropic effect. It is referred to as ***the exclusion restriction assumption***. Here pleiotropy means that one SNP may be associated with multiple phenotypes. More specifically, pleiotropy in MR means that the used instrument is directly associated with the outcome.

Obviously, the validity of MR relies on the validity of the three assumptions above. The relevance assumption (i.e. the first one) can be directly validated based on the observed data using the index SNP information as well as the *F* statistic, while the independence and exclusion restriction assumptions are difficult to examine in practice. In the present study following previous work we validate the last two assumptions through a range of sensitivity analyses (e.g. MR-Egger regression for the pleiotropic effect ).

A SNP that satisfies these assumptions can serve as a valid instrumental variable in MR. Such a SNP potentially divides the general population into various subgroups (Additional file 1: Figure S2B). Moreover, because the two alleles of a SNP are randomly segregated during gamete formation and conception under the law of Mendel and such segregation is independent of many known/unknown confounding factors, MR is thus less susceptible to reverse causation and confounders compared with other study designs . The division by this way is very similar to the setting in a randomized controlled trial where a set of enrolled subjects/individuals are classified into multiple groups and different levels of exposure are assigned (Additional file 1: Figure S2B); and the substantial change of outcome among groups is an indicator of the putative causal relationship between the exposure and the outcome. Therefore, in this sense MR has been often called the nature’s randomized trial and increasingly becomes a powerful statistical tool for causal inference in observational studies .

## Estimation of the causal effect

## Two-stage least squares based on individual-level data set

We now describe the details of MR for estimating the causal effect of an exposure on an outcome using the two-stage least squares method (2SLS) when individual-level data (i.e. exposure, instruments and outcome) are given.

Let *X* be the exposure, *G* be the selected *k* instrumental variables (satisfying the assumptions of MR), and *Y* be the continuous outcome. The causal effect of *X* on *Y* can be easily estimated by 2SLS using the following procedure. Briefly, in the first stage regression, *X* is regressed on the instrumental variables *G* to obtain predicted values of *X* (say $\hat{X}$). In the second stage regression, *Y* is regressed on the predicted values of the exposure$\hat{X}$. Then, the causal effect is the regression coefficient estimated in the second stage regression.

Specifically, we have

$X = \beta_{0}^{x} + \sum_{j = 1}^{k} G_{j}\beta_{j}^{x} + \varepsilon^{x}$,

where $\beta_{0}^{x}$ and $\beta_{j}^{x}$ (*j* = 1, 2, …, *k*) are the intercept term of the exposure and the effect of instrumental variables on the exposure, respectively; and $\varepsilon^{x}$ is the normally distributed residual of the exposure. The predicted values of the exposure $\hat{X}$ can be expressed as

$\hat{X} = \hat{\beta}_{0}^{x} + \sum_{j = 1}^{k} G_{j}\hat{\beta}_{j}^{x}$.

Then, in the second stage regression we have

$Y = \beta_{0}^{y} + \hat{X}\theta+ \varepsilon^{y}$,

where $\beta_{0}^{y}$ and *θ* are the intercept term of the outcome and the causal effect of the exposure on the outcome, respectively; and $\varepsilon^{y}$ is the normally distributed residual of the outcome.

Alternatively, the causal effect *θ* of the exposure on the outcome can be estimated as

$\hat{\theta} = (X^{T}P_{G}X)^{-1}X^{T}P_{G}Y$,

where *P_G_* = $G(G^{T}G)^{-1}G^{T}$ is the projection matrix. Note that the standard error obtained from the second stage regression needs to be corrected to take into account the uncertainty in the first stage regression .

## Wald ratio method

The 2SLS method above is simple to implement but requires the individual-level data, which are not often available. When only summary-level data (e.g. the association statistics for both the exposure and outcome in GWASs) are given, the Wald ratio method (also known as the ratio of coefficients method) is employed to estimate the causal effect. Here we fist suppose that all the variables are continuous and the relationships between variables are linear. Assume the effect size and the variance for instrumental variable *G_j_* on the continuous exposure *X* are $\hat{\beta}_{j}^{x}$ and $\text{var}(\hat{\beta}_{j}^{x})$ (*j* = 1, 2, …, *k*), respectively; and assume that the effect size and the variance for the same instrumental variable *G_j_* on *Y* as $\hat{\beta}_{j}^{y}$ and $\text{var}(\hat{\beta}_{j}^{y})$, respectively. If any of these variables is binary, then these estimates can be replaced with summary association estimates (in the log scale) created from a logistic model.

Then, in terms of the Wald ratio method the causal effect (*θ_j_*) of *X* on *Y* conditional on *G_j_* can be calculated by

$\hat{\theta}_{j} = \frac{\hat{\beta}_{j}^{y}}{\hat{\beta}_{j}^{x}}$.

The asymptotic variance of $\hat{\theta}_{j}$ is yielded with the Delta method based on the normal approximation .

To see the derivation of the Wald ratio estimate in , we return to the 2SLS method in which we now assume only one instrumental variable *G_j_* is involved. We plug in the predicted exposure

$\hat{X} = \hat{\beta}_{0}^{x} + \sum_{j = 1}^{k} G_{j}\hat{\beta}_{j}^{x}$.

into the second-stage regression

$$\begin{matrix} Y = \beta_{0}^{y} + (\hat{\beta}_{0}^{x} + G_{j}\hat{\beta}_{j}^{x})\theta+ \varepsilon^{y}, \\ = \tilde{\beta}_{0}^{y} + G_{j}\hat{\beta}_{j}^{x}\theta+ \varepsilon^{y}, \end{matrix}$$

where $\tilde{\beta}_{0}^{y}$ is the new intercept term. If no pleiotropic effect exists, we approximately have

$\hat{\beta}_{j}^{y} = \hat{\beta}_{j}^{x}\theta$.

Thus, the Wald ratio estimate is obtained.

## Inverse-variance weighted method

When there are multiple instrumental variables, we can combine the single Wald ratio estimate in together by using the fixed-effects or random-effects inverse-variance weighted (IVW) methods . The former assumes the causal effects across instrumental variables are homogeneous, while the latter assumes they are heterogeneous. The two versions of IVW methods produce the estimate of the causal effect $\theta$ through a weighted average manner and have the same estimate

$\hat{\theta} = \frac{1}{\sum_{j=1}^{k} \text{var}(\hat{\beta}_{j}^{y})^{-1}(\hat{\beta}_{j}^{x})^{2}}\sum_{j=1}^{k} \text{var}(\hat{\beta}_{j}^{y})^{-1}\hat{\beta}_{j}^{y}\hat{\beta}_{j}^{x}$.

The estimate in is equivalent to the following weighted linear regression

$\hat{\beta}_{j}^{y} = \hat{\beta}_{j}^{x}\theta+ e_{j}, e_{j} \sim N(0, \text{σ}^{2}\times\text{var}(\hat{\beta}_{j}^{y}))$.

where $\text{σ}^{2}$ is the residual error variance. Note that the intercept disappears in to satisfy the assumption that the outcome is zero when the exposure is zero. When $\text{σ}^{2}$ = 1 model is in fact the fixed-effects IVW method; while when $\text{σ}^{2}$ is larger than 1, it becomes the random-effects IVW method to allow for heterogeneity . The causal effect size heterogeneity across instrumental variables can be examined by the Cochran's Q test and I^2^ statistics . Note that, model is very similar to the multiplicative model which is widely used in the meta-analysis literature .

Note that in the IVW method the false positive rate should be well controlled (i.e. less than 5%, the commonly used significance level as used in the present study) if the causal association between the exposure (e.g. T2D) and the outcome (e.g. ALS) really exists. This is due to the fact that the conclusion was made based on the combination of all the instruments using the IVW method rather than based on a range of multiple tests for each instrument.

## Other methods for causal effect estimation in MR

First, in the MR analysis the leave-one-out (LOO) analysis is widely used to examine potential instrument outliers. In the LOO analysis, we remove an instrumental variable at a time, and re-calculate the causal effect estimate using the remaining instruments through the IVW methods. The discrepancy between the LOO estimate with one instrument left out and the original estimate obtained using all instruments reflects the influence of the instrument that is excluded.

In addition, the weighted median-based method is also commonly employed as a supplementary approach to the IVW method. Compared with the IVW method, the weighted median-based method is more robust against outlying instrumental variables and can provide a consistent estimate for the causal effect when at least 50% of the weight information comes from valid instrumental variables . In the sensitivity analyses of MR, the MR-Egger regression is used to test the assumption of pleiotropic effects . The MR-Egger regression can be considered a modification of the random-effects IVW method

$\hat{\beta}_{j}^{y} =\beta_{0} + \hat{\beta}_{j}^{x}\theta_{\text{Egger}} + e_{j}, e_{j} \sim N(0, \text{σ}^{2}\times\text{var}(\hat{\beta}_{j}^{y}))$,

where $\beta_{0}$ is the intercept, $\theta_{\text{Egger}}$ is the causal effect. When $\beta_{0}$ is exactly zero, then the MR-Egger estimate $\hat{\theta}_{\text{Egger}}$ is equal to the random-effects IVW estimate. Under the assumption of InSIDE (Instrument Strength Independent of Direction Effect) , the intercept term $\beta_{0}$ can be interpreted as the average pleiotropic effect of all the instrumental variables. If the assumption of balanced pleiotropy holds, then on average $\beta_{0}$ is equal to zero, and the MR-Egger estimate $\hat{\theta}_{\text{Egger}}$ is consistent with regards to $\theta$. In contrast, the deviation of $\beta_{0}$ from zero indicates either the presence of pleiotropy or the violation of InSIDE assumption. Therefore, a significantly non-zero intercept term in the MR-Egger regression can lead to bias in the causal effect estimation.

The Mendelian Randomization Pleiotropy RESidual Sum and Outlier (MR-PRESSO) analysis is employed to identify pleiotropic outliers. Like the MR-Egger regression, the MR-PRESSO outlier test also relies on the InSIDE assumption and requires at least 50% of the instruments to be valid . The MR-PRESSO was developed under the framework of residuals in the setting of linear regression. More details of MR-PRESSO can be found in .

## Weak instrument, the *F* statistic and PVE

In our Mendelian randomization analysis, we employ *F* statistic (also known as the Cragg-Donald statistic) ) to evaluate the issue of weak instrument. Specifically, we want to examine whether the selected instrumental variables have relatively strong impacts on exposures used in our analysis as strong instruments guard the asymptotic properties of instrumental variable methods and ensure the unbiased causal effect estimation . The general form of the *F* statistic is

$F_{j} = \frac{\text{PVE}_{j} \times\text{(}N_{j} -1 -k\text{)}}{(1 - \text{PVE}_{j}) \times k}$,

where *k* is the number of instrumental variables (in the case of a single instrumental variable, *k* = 1) and *N_j_* is the effective sample size for instrumental variable *j*. We apply to compute an *F_j_* statistic for each instrumental variable in turn. Here, PVE*_j_* is the proportion of variance explained (PVE) by the examined instrumental variable *j* and is calculated by following the approximation method shown in

$\text{PVE}_{j} \approx\frac{(\hat{\beta}_{j}^{x})^{2}}{(\hat{\beta}_{j}^{x})^{2} + \text{var(}\hat{\beta}_{j}^{x}) \times N_{j}}$,

where $\hat{\beta}_{j}^{x}$ and $\text{var(}\hat{\beta}_{j}^{x})$ are the estimated effect size and variance for instrumental variable *j*. We also generate an overall *F* statistic for all the used instrumental variables jointly

$F = \frac{\text{PVE} \times\text{(}\bar{N} -1 -k\text{)}}{(1 - \text{PVE}) \times k}$,

where $\bar{N}$ is the average sample size across the selected instrumental variables and PVE is the total proportion of variance explained by all the instrumental variables collectively. Because the selected instrumental variables are independent with each other, the total PVE in is a simple summation of individual PVE*_j_*. In the literature a rule of thumb is that an *F* statistic above ten is deemed as an indicator against weak instrument .

Note that, the *F* statistic is traditionally applicable to continuous exposures (e.g. fasting glucose, fasting insulin, HbA1c, blood lipids or body mass index ). For a binary exposure (i.e. T2D in the present study), to measure the strength of the instrumental variable *j*, we make a simple modification by transforming the PVE estimate on the observed scale on the liability scale with a correction factor

$\frac{p_{0}^{2} \times\text{(}1 -p_{0}\text{)}^{2}}{p_{1} \times\text{(}1 -p_{1}\text{)} \times z_{0}^{2}}$,

where *p*_0_ is the overall prevalence of T2D, *p*_1_ is the proportion of cases in the T2D GWAS, *z*_0_ = *ϕ*(*μ*_0_) and *μ*_0_ = Ф^-1^(*p*_0_), with *ϕ* and Ф the normal probability density function and cumulative distribution function, respectively. In our study, *p*_1_ = 0.095 and assume *p*_0_ = 0.085 ; then the correction factor is calculated to be 2.91, meaning that the PVE estimate on the liability scale will be approximately three times larger than the PVE estimate on the observed scale. However, we do note that, because the per-SNP PVE estimates are small, such a transformation often does not affect much the final *F* statistics calculation. In addition, a slightly larger *F* statistic might be obtained after transformation, suggesting that using the *F* statistics computed based on the linear model without transformation can be a conservative strategy to select strong instruments.

In the present study, for T2D we employ the more conservative calculation of the *F* statistic with the PVE estimate on the observed scale in our Mendelian randomization analysis . Therefore, when evaluating whether the instrumental variables have significant and strong association effects with T2D, we directly calculate the *F* statistic shown in and . Doing this is based on the following two reasons: (i) a binary trait can be approximately treated as continuous values (as done in ) as the linear model can be viewed as the first order Taylor approximation to the generalized linear model. Such an approximation is accurate when the effect size of SNP is small — a finding which is frequently observed in GWASs as most complex traits (i.e. T2D) are polygenic and are influenced by a lot of genetic variants with small effects ; (ii) although the lack of rigorously theoretical justification, the same *F* statistic formula has also been applied to binary traits (or to nonlinear instrumental variable models) in the framework of generalized method of moments (e.g. ). Here, for binary exposures, $\hat{\beta}_{j}^{x}$ is the effect size in the log scale and the estimated PVE of T2D for instrumental variable *j* is interpreted at the observed scale within the framework of the liability threshold model .

Alternatively, to assess the strength of the instrument for a binary exposure, some studies suggest to employ the quantity of $(\hat{\beta}_{j}^{x})^{2}/\text{var(}\hat{\beta}_{j}^{x})$, which is an approximation of and follows a distribution of$F_{1, \infty}$ . The same rule of thumb for the *F* statistic in can be applicable. Based on this measurement, the *F* statistic of the instruments of T2D ranges from 29.6 to 1578.3, offering little evidence of weak instruments.

Finally, we acknowledge the direct application of the *F* statistic to binary exposures may be suboptimal and more investigations are warranted.

# References

1. Sleiman PM, Grant SF: **Mendelian randomization in the era of genomewide association studies**. *Clin Chem* 2010, **56**(5):723-728.

2. Lawlor DA, Harbord RM, Sterne JA, Timpson N, Davey Smith G: **Mendelian randomization: using genes as instruments for making causal inferences in epidemiology**. *Stat Med* 2008, **27**(8):1133-1163.

3. Sheehan NA, Didelez V, Burton PR, Tobin MD: **Mendelian randomisation and causal inference in observational epidemiology**. *PLoS Med* 2008, **5**(8):e177.

4. Cragg JG, Donald SG: **Testing Identifiability and Specification in Instrumental Variable Models**. *Econometric Theory* 1993, **9**(2):222-240.

5. Burgess S, Small DS, Thompson SG: **A review of instrumental variable estimators for Mendelian randomization**. *Stat Methods Med Res* 2017, **26**(5):2333-2355.

6. Bowden J, Del Greco M F, Minelli C, Davey Smith G, Sheehan NA, Thompson JR: **Assessing the suitability of summary data for two-sample Mendelian randomization analyses using MR-Egger regression: the role of the I^2^ statistic**. *Int J Epidemiol* 2016, **45**(6):1961-1974.

7. Burgess S, Thompson SG: **Interpreting findings from Mendelian randomization using the MR-Egger method**. *Eur J Epidemiol* 2017, **32**(5):377-389.

8. Davey Smith G, Ebrahim S: **‘Mendelian randomization’: can genetic epidemiology contribute to understanding environmental determinants of disease?** *Int J Epidemiol* 2003, **32**(1):1-22.

9. Hingorani A, Humphries S: **Nature's randomised trials**. *Lancet* 2005, **366**(9501):1906-1908.

10. Thanassoulis G, O'Donnell CJ: **Mendelian randomization: nature's randomized trial in the post-genome era**. *JAMA* 2009, **301**(22):2386-2388.

11. Davies NM, Holmes MV, Davey Smith G: **Reading Mendelian randomisation studies: a guide, glossary, and checklist for clinicians**. *Br Med J* 2018, **362**.

12. Au Yeung SL, Luo S, Schooling CM: **The Impact of Glycated Hemoglobin (HbA1c) on Cardiovascular Disease Risk: A Mendelian Randomization Study Using UK Biobank**. *Diabetes Care* 2018, **41**(9):1991.

13. Au Yeung SL, Lin SL, Lam HSHS, Schooling CM: **Effect of l-arginine, asymmetric dimethylarginine, and symmetric dimethylarginine on ischemic heart disease risk: A Mendelian randomization study**. *Am Heart J* 2016, **182**:54-61.

14. Hartwig FP, Davey Smith G, Bowden J: **Robust inference in summary data Mendelian randomization via the zero modal pleiotropy assumption**. *Int J Epidemiol* 2017, **46**(6):1985-1998.

15. Yavorska OO, Burgess S: **MendelianRandomization: an R package for performing Mendelian randomization analyses using summarized data**. *Int J Epidemiol* 2017:dyx034.

16. Brockwell SE, Gordon IR: **A comparison of statistical methods for meta-analysis**. *Stat Med* 2001, **20**(6):825-840.

17. DerSimonian R, Laird N: **Meta-analysis in clinical trials**. *Control Clin Trials* 1986, **7**(3):177-188.

18. Thompson SG, Sharp SJ: **Explaining heterogeneity in meta-analysis: A comparison of methods**. *Stat Med* 1999, **18**(20):2693-2708.

19. Higgins JPT, Thompson SG, Spiegelhalter DJ: **A re-evaluation of random-effects meta-analysis**. *Journal of the Royal Statistical Society Series A, (Statistics in Society)* 2009, **172**(1):137-159.

20. Noyce AJ, Kia DA, Hemani G, Nicolas A, Price TR, De Pablo-Fernandez E, Haycock PC, Lewis PA, Foltynie T, Davey Smith G *et al*: **Estimating the causal influence of body mass index on risk of Parkinson disease: A Mendelian randomisation study**. *PLoS Med* 2017, **14**(6):e1002314.

21. Bowden J, Davey Smith G, Haycock PC, Burgess S: **Consistent estimation in Mendelian randomization with some invalid instruments using a weighted median estimator**. *Genet Epidemiol* 2016, **40**(4):304-314.

22. Verbanck M, Chen C-Y, Neale B, Do R: **Detection of widespread horizontal pleiotropy in causal relationships inferred from Mendelian randomization between complex traits and diseases**. *Nat Genet* 2018, **50**(5):693-698.

23. Burgess S, Thompson SG: **Avoiding bias from weak instruments in Mendelian randomization studies**. *Int J Epidemiol* 2011, **40**(3):755-764.

24. Keele L, Morgan JW: **How strong is strong enough? Strengthening instruments through matching and weak instrument tests**. *Ann Appl Stat* 2016(2):1086-1106.

25. Burgess S, Thompson SG: **Improving bias and coverage in instrumental variable analysis with weak instruments for continuous and binary outcomes**. *Stat Med* 2012, **31**(15):1582-1600.

26. Bound J, Jaeger DA, Baker RM: **Problems with instrumental variables estimation when the correlation between the instruments and the endogenous explanatory variable is weak**. *J Am Stat Assoc* 1995, **90**(430):443-450.

27. Shim H, Chasman DI, Smith JD, Mora S, Ridker PM, Nickerson DA, Krauss RM, Stephens M: **A Multivariate Genome-Wide Association Analysis of 10 LDL Subfractions, and Their Response to Statin Treatment, in 1868 Caucasians**. *PLoS ONE* 2015, **10**(4):e0120758.

28. Zeng P, Zhou X: **Causal Association Between Birth Weight and Adult Diseases: Evidence From a Mendelian Randomization Analysis**. *Frontiers in Genetics* 2019, **10**(618).

29. Staiger D, Stock JH: **Instrumental variables regression with weak instruments**. *Econometrica* 1997, **65**(3):557-586.

30. Stock J, Yogo M, Wright J: **A Survey of Weak Instruments and Weak Identification in Generalized Method of Moments**. *Journal of Business and Economic Statistics* 2002, **20**(4):518-529.

31. Pierce BL, Ahsan H, VanderWeele TJ: **Power and instrument strength requirements for Mendelian randomization studies using multiple genetic variants**. *Int J Epidemiol* 2011, **40**(3):740-752.

32. Zeng P, Zhou X: **Causal effects of blood lipids on amyotrophic lateral sclerosis: a Mendelian randomization study**. *Hum Mol Genet* 2019, **28**(4):688-697.

33. Lyall DM, Celis-Morales C, Ward J, et al.: **Association of body mass index with cardiometabolic disease in the uk biobank: A mendelian randomization study**. *JAMA Cardiology* 2017, **2**(8):882-889.

34. Zhou X, Carbonetto P, Stephens M: **Polygenic modeling with Bayesian sparse linear mixed models**. *PLoS Genet* 2013, **9**(2):e1003264.

35. Lee SH, Wray NR, Goddard ME, Visscher PM: **Estimating missing heritability for disease from genome-wide association studies**. *Am J Hum Genet* 2011, **88**(3):294-305.

36. The Emerging Risk Factors Collaboration: **Diabetes mellitus, fasting blood glucose concentration, and risk of vascular disease: a collaborative meta-analysis of 102 prospective studies**. *Lancet* 2010, **375**(9733):2215-2222.

37. Speed D, Balding DJ: **MultiBLUP: improved SNP-based prediction for complex traits**. *Genome Res* 2014, **24**(9):1550-1557.

38. Moser G, Lee SH, Hayes BJ, Goddard ME, Wray NR, Visscher PM: **Simultaneous Discovery, Estimation and Prediction Analysis of Complex Traits Using a Bayesian Mixture Model**. *PLoS Genet* 2015, **11**(4):e1004969.

39. Weissbrod O, Geiger D, Rosset S: **Multikernel: linear mixed models for complex phenotype prediction**. *Genome Res* 2016, **26**(7):969-979.

40. Zeng P, Hao X, Zhou X: **Pleiotropic mapping and annotation selection in genome-wide association studies with penalized Gaussian mixture models**. *Bioinformatics* 2018, **34**(16):2797-2807.

41. Visscher PM, Wray NR, Zhang Q, Sklar P, McCarthy MI, Brown MA, Yang J: **10 Years of GWAS Discovery: Biology, Function, and Translation**. *Am J Hum Genet* 2017, **101**(1):5-22.

42. Bulik-Sullivan B, Finucane HK, Anttila V, Gusev A, Day FR, Loh P-R, ReproGen C, Psychiatric Genomics C, Genetic Consortium for Anorexia Nervosa of the Wellcome Trust Case Control C, Duncan L *et al*: **An atlas of genetic correlations across human diseases and traits**. *Nat Genet* 2015, **47**(11):1236-1241.

43. Bulik-Sullivan BK, Loh P-R, Finucane HK, Ripke S, Yang J, Patterson N, Daly MJ, Price AL, Neale BM, Consortium SWGotPG: **LD Score regression distinguishes confounding from polygenicity in genome-wide association studies**. *Nat Genet* 2015, **47**(3):291-295.

44. Finucane HK, Bulik-Sullivan B, Gusev A, Trynka G, Reshef Y, Loh P-R, Anttila V, Xu H, Zang C, Farh K: **Partitioning heritability by functional annotation using genome-wide association summary statistics**. *Nat Genet* 2015, **47**(11):1228-1235.

45. Weissbrod O, Flint J, Rosset S: **Estimating Heritability and Genetic Correlation in Case Control Studies Directly and with Summary Statistics**. *bioRxiv* 2018.

46. Speed D, Balding DJ: **SumHer better estimates the SNP heritability of complex traits from summary statistics**. *Nat Genet* 2019, **51**(2):277-284.
