# Supplementary material for: Causal association of type 2 diabetes with amyotrophic lateral sclerosis: new evidence from Mendelian randomization using GWAS summary statistics
Source: BMC Med. 2019 Dec 4;17:225. doi: 10.1186/s12916-019-1448-9 (PMC6892209; doi:10.1186/s12916-019-1448-9)
Supplement: Supplementary file 3 — Additional file 3. Description of GWAS Data Sets. [file 12916_2019_1448_MOESM3_ESM.doc]

**Additional file 3**

# Description of GWAS Data Sets

## Type 2 diabetes

For the European population, we obtained the summary association result of type 2 diabetes (T2D) from one of the largest GWASs to date , which was a genome-wide meta-analysis on three previous T2D studies:

**DIAGRAM** (DIAbetes Genetics Replication and Meta-analysis, DIAGRAM) : this data included two stages of summary statistics. In the first stage, it included 12 GWAS cohorts of European descent up to a total of 12,171 T2D cases and 56,862 controls. Sample and single nucleotide polymorphism (SNP) quality control were performed within each cohort. For each the sub-GWAS, ~2.5 million SNPs were imputed based on the CEU reference panel of the International HapMap Project Phase II . Then each SNP (with minor allele frequency > 1%) was analyzed using an additive logistic model while adjusting for study-specific covariates (e.g. age and sex) and indicators of population structure. In the second stage, a total of 22,669 cases and 58,119 controls were analyzed. The summary association statistics in the two stages were combined with the fixed-effects meta-analysis. The Summary statistics generated in DIAGRAM were further imputed based on the 1000 Genomes Project Phase I using a summary data based imputation method called ImpG .

**GERA** (Genetic Epidemiology Research on Aging, GERA) : a total of 6,905 cases and 46,983 controls were included after removing related individuals based on the genetic relationship matrix at a genetic relatedness threshold of 0.05. Next, after quality control (SNPs with missing rate > 0.02, Hardy Weinberg equilibrium test p value < 1.00E-6 or minor allele count < 1 and individuals with missing rate > 0.02), the SNP genotypes were imputed based on the 1000 Genomes Projects reference panel using IMPUTE . The association analysis was performed using PLINK with sex, age and the first 20 principal components as covariates.

**UK Biobank cohort** : after cleaning and imputation based on the reference panel of the Haplotype Reference Consortium (HPC) (those data processes were implemented by the UK Biobank cohort team) , there were 21,147 cases and 434,460 controls as well as 18,138,214 SNPs in the UK Biobank T2D data set. The association analysis was conducted using BOLT-LMM with sex and age as covariates. In the BOLT-LMM analysis, the relatedness, population stratification and polygenic effects were adjusted based on a set of linkage equilibrium pruned genetic variants (i.e. 711,933 SNPs with low correlation). Then, the effect size estimated from BOLT-LMM was transformed on the observed 0-1 scale to odds ratio using LMOR (http://cnsgenomics.com/shiny/LMOR/).

Those three summary association statistics data sets were pooled with the fixed-effects inverse variance-weighted meta-analysis. Finally, the T2D GWAS contained a total of ~5 million genotyped and imputed SNPs for 659,316 (62,892 cases and 596,424 controls) individuals of European ancestry . After the PLINK clumping procedure (*r*^2^ threshold = 0.01 and window size = 1Mb) with linkage equilibrium estimated from the UK Biobank cohort genotypes, there were 139 independent SNPs at the genome-wide significance level of 5.00E-8 . After removing potential index SNPs with pleiotropic effects, 67 genetic variants were finally selected to serve as instrumental variables for T2D in the main analysis in our study.

We also repeated our analysis using another set of instruments obtained from another T2D GWAS data, which combined 32 sub-studies up to 74,124 cases and 824,006 controls of European ancestry . The SNP genotypes were imputed to the HPC reference panel using minimac3 or IMPUTE . After qualify control (minor allele count < 5 combined in cases and controls, imputation accuracy < 0.30 if minimac3 or proper information < 0.40 if IMPUTE, standard error of the allelic log odds ratio > 10), there included ~27 million SNPs. Within each study, each SNP was tested for association using an additive logistic regression with adjustment for study-specific covariates and principal components for population structure control. Afterwards, the fixed-effects meta-analysis was employed to combine all the studies. Using the same selection procedure described in Fig 1, after removing potential instrumental outliers, we obtained a total of 90 instruments with high imputation accuracy (>0.95). We had to use the MR-Egger regression instead of standard IVW methods for this analysis, because the intercept in the MR-Egger regression is estimated to be statistically significantly different from zero (estimated intercept = 0.010; 95% CI 0.001 - 0.020, *p* = 0.035). The estimated causal effect of T2D on ALS with the MR-Egger regression is 0.86 (95% CI 0.73 - 1.01, *p* = 0.068), which is marginally significant at the level of 0.05 and consistent with our main results. Note that due to the lower statistical power of MR-Egger regression compared to the standard IVW methods, the significance level obtained using this new set of instruments is not as strong as the main results in our study (e.g. Table 3).

For the East Asian population, we obtained summary association results of T2D from the consortium of Japanese ENcyclopedia of GEnetic associations by Riken (JENGER) . Specifically, 36,614 cases and 155,150 controls from four GWASs of the BioBank Japan project were included. The SNP genotypes imputation was run with minimac3 using 1000 Genomes Project Phase III as a reference panel . After quality control (heterozygosity count < 5 or minor allele frequency < 0.01, Hardy Weinberg equilibrium p value < 1.00E-6, concordance rate < 0.99 with in-house whole-genome-sequence data using overlapping samples, SNP genotype call rate < 0.99 and differential missingness p value < 1.00E-6) , a total of ~12 million genotyped and imputed SNPs were kept. The association analysis was performed independently in each GWAS using an additive logistic model while controlling for sex, age and the first ten principal components. Finally, the association was combined with the fixed-effect meta-analysis. There were 34 index SNPs employed to serve as instrumental variables by using the similar PLINK clumping procedure above .

## Amyotrophic lateral sclerosis

The amyotrophic lateral sclerosis (ALS) GWAS data set used in the present study was the largest ALS GWAS to date in the European population up on 80,610 individuals (20,806 cases and 59,804 controls) . The SNP genotypes imputation was conducted with minimac3 using 1000 Genomes Project Phase III as a reference panel . After quantify control (SNP genotype call rates < 0.975, non-European ancestry, abnormal *F* inbreeding coefficient, mismatch between phenotypic and genotypic gender, cryptic relatedness defined as identity-by-descent proportion of inheritance > 0.125 and imputation accuracy < 0.30), 10,031,417 genotyped and imputed SNPs were left. Logistic regression was performed in each sub-study with a set of principal components, age and gender as covariates. The association analysis was finally implemented using the fixed-effect weighted method across sub-studies.

The East Asian ALS GWAS was performed on 4,084 Chinese individuals (1,234 cases and 2,850 controls). The SNP genotypes imputation was performed with IMPUTE using 1000 Genomes Project Phase I as a reference panel . Finally, 6,613,544 genotyped and imputed SNP were kept after quality control (SNP genotype call rate < 0.99, minor allele frequency < 0.01, Hardy-Weinberg equilibrium p value < 1.00E-6, differential missingness in genotypes between cases and controls with p value < 1.00E-6) . Similarly, logistic regression was conducted for each SNP with the first five principal components as covariates.

## Body mass index

The study of body mass index (BMI) included 339,224 individuals of European ancestry from 125 sub-studies. The SNP genotypes imputation was performed based on the International HapMap Project II CEU reference panel , with quality control followed by the procedure as described in . Then, 2,554,637 genotyped and imputed SNPs were retained and meta-analyzed. BMI was first adjusted for age, age^2^ and study-specific covariates in a linear regression model and genetic relatedness was also adjusted for in a study-specific manner. The resulting residuals were then quintile normalized to a standard normal distribution before association analysis. Finally, the association was conducted with a linear model for each SNP and combined with the fixed-effects meta-analysis.

## Fasting insulin, fasting glucose and hemoglobin A1c

We obtained summary association statistics of fasting insulin and glucose from the Meta-Analyses of Glucose and Insulin-related traits Consortium (MAGIC) . Before association analysis, the SNP genotypes imputation was implemented based on the HapMap Phase II CEU reference panel . Finally, this data set included 2,470,476 SNPs for fasting glucose up to 133,010 non-diabetic European individuals, and 2,461,105 SNPs for fasting insulin (log-transformed) up to 108,557 European individuals after quality control (SNP genotyping call rate < 0.95, sex discrepancies, ancestry outliers, heterozygosity, minor allele frequency < 0.01, Hardy Weinberg equilibrium p value < 1.00E-4, SNP effect estimate standard error ≥ 10 and minor allele count < 10). The association was performed in each sub-study with a linear regression while adjusting for age, gender, study site and geographic covariates and then was combined via the fixed-effects meta-analysis.

We obtained the summary association statistics of hemoglobin A1c from , in which 123,665 European individuals were included. The SNP genotypes imputation was carried out using the 1000 Genomes Project Phase II as a reference panel . After quality control (Hardy Weinberg equilibrium p value < 1.00E-6, SNP genotyping call rate < 0.95, minor allele frequency < 0.01 and imputation accuracy < 0.40), 2,586,698 SNPs were retained. The association was performed with a linear additive genetic regression with a 1 d.f. trend test while adjusting for age, age^2^, sex, cohort and population structure when available.

Finally, we interpreted the causal effect of a continuous exposure on ALS with odds ratio (OR) on the scale of one-unit change of the exposure of interest (i.e. mmol/L for fasting glucose, pmol/L for log-transformed fasting insulin and % for HbA1c). Note that in the original BMI GWAS paper , BMI was standardized to have mean zero and standard deviation (SD) of one before association analysis and the effect sizes of the instruments were computed on the standardized scale, meaning that one-unit change represented one SD change of BMI. Therefore, for BMI the OR estimate was also measured in terms of OR per one SD change. We thus referred to the one SD of BMI as one unit in the present study. In addition, since only summary-level data can be available for us, it was impossible to compute the SD of BMI exactly. We had to compute the mean of the SD approximately across all the sub-studies, resulting in the mean of the SD being ~4.8 kg/m^2^ in terms of all 157 BMI sub-studies . Thus, the one-unit change of BMI was about 4.8 kg/m^2^. For a binary exposure (i.e. T2D), the OR was interpreted by comparing the risk of the case with that of the control.

# References

1. Xue A, Wu Y, Zhu Z, Zhang F, Kemper KE, Zheng Z, Yengo L, Lloyd-Jones LR, Sidorenko J, Wu Y *et al*: **Genome-wide association analyses identify 143 risk variants and putative regulatory mechanisms for type 2 diabetes**. *Nat Commun* 2018, **9**(1):2941.

2. Morris AP, Voight BF, Teslovich TM, Ferreira T, Segrè AV, Steinthorsdottir V, Strawbridge RJ, Khan H, Grallert H, Mahajan A *et al*: **Large-scale association analysis provides insights into the genetic architecture and pathophysiology of type 2 diabetes**. *Nat Genet* 2012, **44**(9):981-990.

3. International HapMap Consortium.: **A second generation human haplotype map of over 3.1 million SNPs**. *Nature* 2007, **449**(7164):851 - 861.

4. 1000 Genomes Project Consortium: **An integrated map of genetic variation from 1,092 human genomes**. *Nature* 2012, **491**(7422):56-65.

5. Pasaniuc B, Zaitlen N, Shi H, Bhatia G, Gusev A, Pickrell J, Hirschhorn J, Strachan DP, Patterson N, Price AL: **Fast and accurate imputation of summary statistics enhances evidence of functional enrichment**. *Bioinformatics* 2014, **30**(20):2906-2914.

6. Banda Y, Kvale MN, Hoffmann TJ, Hesselson SE, Ranatunga D, Tang H, Sabatti C, Croen LA, Dispensa BP, Henderson M: **Characterizing race/ethnicity and genetic ancestry for 100,000 subjects in the Genetic Epidemiology Research on Adult Health and Aging (GERA) cohort**. *Genetics* 2015, **200**(4):1285-1295.

7. Howie BN, Donnelly P, Marchini J: **A Flexible and Accurate Genotype Imputation Method for the Next Generation of Genome-Wide Association Studies**. *PLoS Genet* 2009, **5**(6):e1000529.

8. Purcell S, Neale B, Todd-Brown K, Thomas L, Ferreira MAR, Bender D: **PLINK: a toolset for whole-genome association and population-based linkage analysis**. *Am J Hum Genet* 2007, **81**:559-575.

9. Bycroft C, Freeman C, Petkova D, Band G, Elliott LT, Sharp K, Motyer A, Vukcevic D, Delaneau O, Connell J *et al*: **Genome-wide genetic data on ~500,000 UK Biobank participants**. *bioRxiv* 2017, **https://doi.org/10.1101/166298**.

10. McCarthy S, Das S, Kretzschmar W, Delaneau O, Wood AR, Teumer A, Kang HM, Fuchsberger C, Danecek P, Sharp K *et al*: **A reference panel of 64,976 haplotypes for genotype imputation**. *Nat Genet* 2016, **48**(10):1279-1283.

11. Loh P-R, Tucker G, Bulik-Sullivan BK, Vilhjálmsson BJ, Finucane HK, Salem RM, Chasman DI, Ridker PM, Neale BM, Berger B *et al*: **Efficient Bayesian mixed-model analysis increases association power in large cohorts**. *Nat Genet* 2015, **47**(3):284-290.

12. Mahajan A, Taliun D, Thurner M, Robertson NR, Torres JM, Rayner NW, Payne AJ, Steinthorsdottir V, Scott RA, Grarup N *et al*: **Fine-mapping type 2 diabetes loci to single-variant resolution using high-density imputation and islet-specific epigenome maps**. *Nat Genet* 2018, **50**(11):1505-1513.

13. Das S, Forer L, Schonherr S, Sidore C, Locke AE, Kwong A, Vrieze SI, Chew EY, Levy S, McGue M *et al*: **Next-generation genotype imputation service and methods**. *Nat Genet* 2016, **48**(10):1284-1287.

14. Marchini J: **A new multipoint method for genome-wide association studies by imputation of genotypes**. *Nat Genet* 2007, **39**(7):906-913.

15. Suzuki K, Akiyama M, Ishigaki K, Kanai M, Hosoe J, Shojima N, Hozawa A, Kadota A, Kuriki K, Naito M *et al*: **Identification of 28 new susceptibility loci for type 2 diabetes in the Japanese population**. *Nat Genet* 2019, **51**(3):379-386.

16. Nicolas A, Kenna KP, Renton AE, Ticozzi N, Faghri F, Chia R, Dominov JA, Kenna BJ, Nalls MA, Keagle P *et al*: **Genome-wide Analyses Identify KIF5A as a Novel ALS Gene**. *Neuron* 2018, **97**(6):1268-1283.e1266.

17. Benyamin B, He J, Zhao Q, Gratten J, Garton F, Leo PJ, Liu Z, Mangelsdorf M, Al-Chalabi A, Anderson L: **Cross-ethnic meta-analysis identifies association of the GPX3-TNIP1 locus with amyotrophic lateral sclerosis**. *Nat Commun* 2017, **8**(1):611.

18. Locke AE, Kahali B, Berndt SI, Justice AE, Pers TH, Day FR, Powell C, Vedantam S, Buchkovich ML, Yang J: **Genetic studies of body mass index yield new insights for obesity biology**. *Nature* 2015, **518**(7538):197-206.

19. Winkler TW, Day FR, Croteau-Chonka DC, Wood AR, Locke AE, Mägi R, Ferreira T, Fall T, Graff M, Justice AE *et al*: **Quality control and conduct of genome-wide association meta-analyses**. *Nat Protocols* 2014, **9**(5):1192-1212.

20. Scott RA, Lagou V, Welch RP, Wheeler E, Montasser ME, Luan Ja, Magi R, Strawbridge RJ, Rehnberg E, Gustafsson S *et al*: **Large-scale association analyses identify new loci influencing glycemic traits and provide insight into the underlying biological pathways**. *Nat Genet* 2012, **44**(9):991-1005.

21. Wheeler E, Leong A, Liu C-T, Hivert M-F, Strawbridge RJ, Podmore C, Li M, Yao J, Sim X, Hong J *et al*: **Impact of common genetic determinants of Hemoglobin A1c on type 2 diabetes risk and diagnosis in ancestrally diverse populations: A transethnic genome-wide meta-analysis**. *PLoS Med* 2017, **14**(9):e1002383.
